# CeCl_3_/*n*‐BuLi: Unraveling Imamoto's Organocerium Reagent

**DOI:** 10.1002/anie.202103889

**Published:** 2021-06-08

**Authors:** Tassilo Berger, Jakob Lebon, Cäcilia Maichle‐Mössmer, Reiner Anwander

**Affiliations:** ^1^ Institut für Anorganische Chemie Eberhard Karls Universität Tübingen Auf der Morgenstelle 18 72076 Tübingen Germany

**Keywords:** ^7^Li NMR spectroscopy, carbonyl alkylation, cerium, lithium, *n*-butyl

## Abstract

CeCl_3_(thf) reacts at low temperatures with MeLi, t‐BuLi, and n‐BuLi to isolable organocerium complexes. Solvent‐dependent extensive n‐BuLi dissociation is revealed by ^7^Li NMR spectroscopy, suggesting “Ce(*n‐*Bu)_3_(thf)_x_” or solvent‐separated ion pairs like “[Li(thf)_4_][Ce(*n‐*Bu)_4_(thf)_y_]” as the dominant species of the Imamoto reagent. The stability of complexes Li_3_Ln(n‐Bu)_6_(thf)_4_ increases markedly with decreasing Ln^III^ size. Closer inspection of the solution behavior of crystalline Li_3_Lu(n‐Bu)_6_(thf)_4_ and mixtures of LuCl_3_(thf)_2_/n‐BuLi in THF indicates occurring n‐BuLi dissociation only at molar ratios of <1:3. n‐BuLi‐depleted complex LiLu(n‐Bu)_3_Cl(tmeda)_2_ was obtained by treatment of Li_2_Lu(n‐Bu)_5_(tmeda)_2_ with ClSiMe_3_, at the expense of LiCl incorporation. Imamoto's ketone/tertiary alcohol transformation was examined with 1,3‐diphenylpropan‐2‐one, affording 99 % of alcohol.

## Graphical abstract

Seamless low‐temperature syntheses, crystallization and spectroscopies provide structural details of the heterobimetallic reagent routinely used for regioselective carbon−carbon bond forming reactions with carbonyl compounds. ^7^Li NMR spectroscopy takes on an important role here, tracking down *n*‐BuLi dissociation.

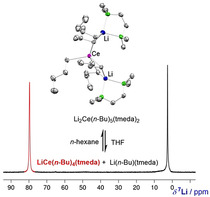

## Introduction

The redox reagents *C*eric *A*mmonium *N*itrate (CAN=(NH_4_)_2_Ce(NO_3_)_6_) and SmI_2_(thf)_2_ as well as the binary alkylating agents CeCl_3_/LiR (R=alkyl like CH_3_ or *n*‐C_4_H_9_) constitute the most commonly employed rare‐earth‐metal reagents in organic transformations (including natural product synthesis).[^1^, ^2^, ^3^, ^4^] The cause of reactivity of the redox‐active compounds is well understood,[^2^, ^3^] and their crystal structures were revealed by X‐ray diffraction (XRD) analyses.[^5^, ^6^] In the solid state, CAN exhibits a three‐dimensional network of 12‐coordinate hexanitratocerate anions and ammonium cations interconnected by hydrogen bonding.^[5]^ On the other hand, samarium diiodide crystallizes as a monomeric pentasolvate, SmI_2_(thf)_5_, from THF solution.^[6]^ Due to the extreme air and moisture sensitivity and combined thermal instability, a detailed structural investigation of organocerium reagents has remained elusive.

**Figure 1:**
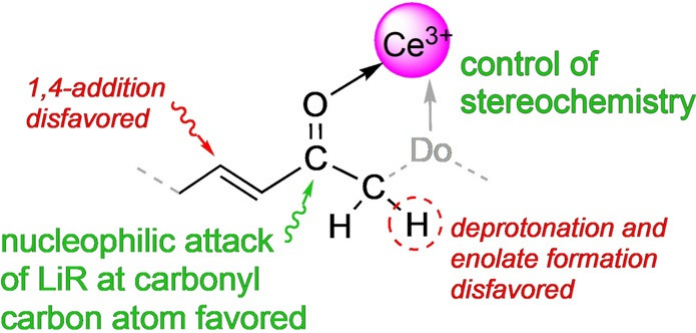
Ce^III^−carbonyl coordination directs highly selective nucleophilic addition reactions of binary CeX_3_/RLi (X=halogenido; R=alkyl).

In 1984, Tsuneo Imamoto et al. described the use of binary mixtures CeI_3_/RLi (R=Me, Et, *n*‐Bu, *sec*Bu, Ph) and CeCl_3_/RLi (R=*n*‐Bu, *t*Bu) as effective reagents for regioselective carbon−carbon‐bond forming with various carbonyl compounds.^[7]^ The best results were obtained when employing equimolar mixtures at −78 °C to −65 °C. Like for the Luche reagent (CeCl_3_(H_2_O)_7_/NaBH_4_),^[8]^ cerium was launched as the least expensive rare‐earth metal while a greater part of transformations was performed with *n*‐BuLi as an easy‐to‐handle (as well as the cheapest) organolithium derivative.^[9]^ Figure 1 depicts characteristic features of the nucleophilic addition of such organocerium reagents to carbonyl compounds, including smooth and selective 1,2‐addition in case of α,β‐unsaturated or easily enolizable substrate molecules,^[7]^ functional group tolerance^[10]^ as well as diastereocontrol via chelate coordination.^[11]^

The much improved selectivity compared to organolithium or Grignard reagents was assigned to a changed basicity of the organocerium reagent and enhanced hardness of the carbonyl carbon atom.^[4]^ The latter originates from the strong oxophilicity of the trivalent cerium. Although Imamoto's seminal organocerium reagents embarked on a new and prosperous branch of organolanthanide chemistry, very few studies exist that target the structural elucidation of such bimetallic mixtures.^[12]^ In sharp contrast, heterobimetallic main‐group organometallic reagents have been given much greater attention, and entitled modern ate chemistry.^[13]^

Imamoto‐type rare‐earth‐metal reagents, more recently employed for halogen‐rare‐earth‐metal exchange reactions^[14]^ or Zweifel olefinations,^[15]^ are as a rule generated in situ and have been designated “*n*‐Bu_2_LaCl⋅4 LiCl”,^[16]^ “*n*‐Bu_3_Sm⋅5 LiCl”,^[17]^ or simply “*n*‐Bu_3_Ce”.^[15]^ The formulas were derived from X‐ray absorption fine structure (EXAFS) studies^[18]^ or Raman spectroscopy.^[15]^ Previous enlightening studies on the CeCl_3_/RLi binary system focused mainly on the composition and activation of the cerous chloride precursor[^12^, ^19^, ^20^] as well as the effect/effectiveness of reagent stoichiometry.^[21]^ It was revealed that the generally applied thermal activation of the commercially available heptahydrate CeCl_3_(H_2_O)_7_ not only generates a material of composition [CeCl_3_(H_2_O)]_*n*_ ^[12c]^ but also benefits from sonication.^[20]^ Importantly, organocerium addition to hydrazones was found most effective and selective for a 1:1 stoichiometry of CeCl_3_/MeLi, but the active reagent formed at different stoichiometries was proposed to be a trimethylcerium species (supported by unreacted CeCl_3_).^[21]^ Although this latter investigation “precluded firm conclusions”, the results “do point out the fallacy of ascribing reagent composition on the basis of mixing stoichiometry especially at low loadings of alkyllithium”.^[21]^

Herein we describe the successful isolation and structural characterization of rare‐earth‐metal *n*‐butyl complexes formed in LnCl_3_/*n*‐BuLi systems devoid of ancillary ligands. NMR spectroscopic studies involving the ^7^Li nucleus provide valuable insights into the solution behavior of such binary mixtures, pointing to the true organocerium species of the Imamoto reagent.

## Results and Discussion

**Figure 2:**
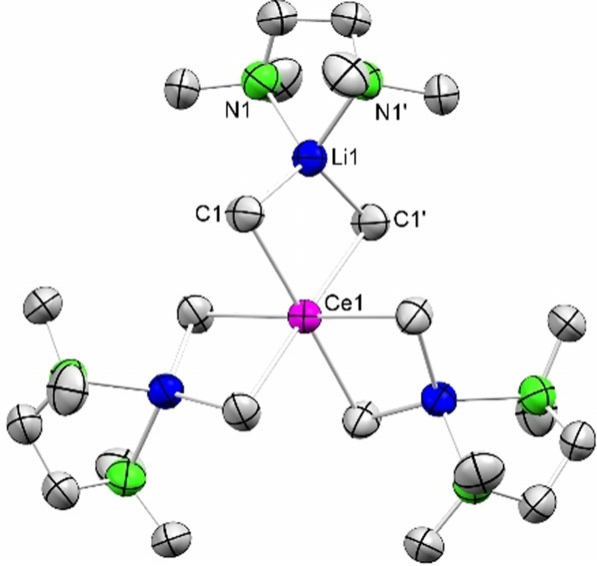
Crystal structure of Li_3_Ce(CH_3_)_6_(tmeda)_3_ (**1**).^[59]^ Atomic displacement ellipsoids set at 50 % probability. Hydrogen atoms omitted for clarity. Selected interatomic distances [Å] and angles [°]: Ce1–C1 2.6795(19), Li1–C1 2.205(3), Li1–N1 2.106(3); C1‐Ce1‐C1′ 88.05(9).

**Synthesis and Solid‐State Structure of the Organocerium Derivatives Li_3_Ce(CH_3_)_6_(tmeda)_3_ and [Li(thf)_4_][Ce*t*Bu_4_]**. For assessing the CeCl_3_/LiR salt‐metathesis protocol we initially probed the methyl derivative, since this would rule out β‐H elimination as a potential decomposition pathway. Complexes of the type Li_3_LnMe_6_(tmeda)_3_ featuring the entire lanthanide series except for promethium and europium were accessed by Schumann et al. as early as 1978, via mixtures LnCl_3_/LiMe/OEt_2_/TMEDA (TMEDA=tetramethylethylenediamine).^[22]^ Solid‐state structures applying XRD analysis were described for the rare‐earth metals erbium^[22b]^ and holmium.^[22c]^ Moreover, both the stabilizing effect of chelating tmeda, teeda (=tetraethylethylenediamine), and dme (=dimethoxyethylene) coligands and the enhanced instability of derivatives of the “lighter” and larger‐sized rare‐earth metals have been emphasized.^[23]^ This is in accord with more recent findings by Okuda et al. on the stability of Li_3_LnMe_6_(thf)_*x*_ (isolable for Ln smaller than Eu), which form the pentametallic ate complexes Li_3_Sc_2_Me_9_(thf)_2_(OEt_2_)_3_ and Li_3_Ln_2_Me_9_(thf)_3_(OEt_2_)_2_ (Ln=Y, Tb), when crystallized from diethyl ether solutions.^[24]^ Such a Ln^III^‐size dependency on thermal stability is commonly observed in rare‐earth‐metal alkyl chemistry^[25]^ and showcased for derivatives Ln(CH_2_SiMe_3_)_3_(thf)_*x*_,^[26]^ [Li(dme)_3_][Ln*t*Bu_4_] (see below),^[27]^ and solvent‐free [LnMe_3_]_*n*_.^[28]^ Since the cerium derivative Li_3_Ce(CH_3_)_6_(tmeda)_3_ (**1**) was only mentioned briefly as an impure product (*δ*_CH3_=−6.4 ppm),[^22^, ^23^] we re‐visited its synthesis applying a slightly modified version of the Schumann protocol. Accordingly, cerous methyl complex **1** could be synthesized at −10 °C in good yield (83 %), and was obtained in analytically pure, single‐crystalline form. The solid‐state structure of complex **1** turned out to be isostructural to the derivatives of the considerably smaller‐sized erbium and holmium (Figure 2).^[22]^

The ^1^H NMR spectrum revealed the methyl signal at *δ*=−4.08 ppm, reflecting a significant paramagnetic shift induced by Ce^III^ (Supporting Information, Figure S4). As expected, the Ce−C distance of 2.6795(19) Å in **1** is considerably longer than those in the holmium (2.563(18) Å)^[22b]^ and erbium congeners (2.57(2) Å).^[22c]^ For further comparison, the Ce−C distances in 6‐coordinate Ce(CH_2_Ph)_3_(thf)_3_ and Ce(AlMe_4_)_3_ fall in the range 2.600(2)–2.614(2) Å^[29]^ and 2.620(7)–2.646(8) Å,^[30]^ respectively, while those in formally 3‐coordinate complexes Ce[CH(SiMe_3_)_2_]_3_ and Ce[C(SiHMe_2_)_3_]_3_ were detected at 2.475(7) Å^[31]^ and 2.651(2)/2.659(2)/2.672(2) Å, respectively.^[32]^

**Figure 3:**
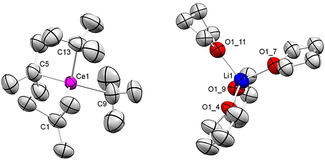
Crystal structure of [Li(thf)_4_][Ce(*t*‐Bu)_4_] (**2**).^[59]^ Atomic displacement ellipsoids set at 30 % probability. Hydrogen atoms omitted for clarity. Selected interatomic distances [Å] and angles [°]: Ce1–C1 2.524(9), Ce1–C5 2.513(8), Ce1–C9 2.50(2), Ce1–C13 2.54(2); C‐Ce1‐C (range) 105.9(10)–111.8(8).

Homoleptic anionic *tert*‐butyl complexes were previously reported for [Li(thf)_*x*_][Ln(*t*‐Bu)_4_] (Ln=Sm, Er: *x=*4; Y: *x=*3),^[27a]^ [Li(OEt_2_)_4_][Er(*t*‐Bu)_4_],^[22c]^ [Li(tmeda)_2_][Ln(*t*‐Bu)_4_] (Ln=Tb, Lu),[^22c^, ^27b^] and [Li(dme)_3_][Ln(*t*‐Bu)_4_] (Ln=Tb, Er).^[27c]^ Crystal structures were obtained for [Li(tmeda)_2_][Lu(*t*‐Bu)_4_]^[27b]^ and [Li(dme)_3_][Er(*t*‐Bu)_4_],^[27c]^ whereas the enhanced thermal instability of derivatives of the “lighter” rare‐earth metals was pointed out. In order to test our low‐temperature set‐up for organocerium derivatives prone to β‐H elimination,^[33]^ we targeted the anionic fragment [Ce(*t*‐Bu)_4_]. Although the mixture CeCl_3_(thf)/*t*‐BuLi/THF gave access to complex [Li(thf)_4_][Ce(*t*‐Bu)_4_] (**2**) at −40 °C, single crystals could be obtained only from very concentrated, oily residues, not allowing for decent elemental analysis (^1^H NMR spectrum: *δ*_*t*Bu_=2.39 ppm, Figure S7). Notwithstanding an XRD analysis revealed a 4‐coordinate cerium center (Figure 3), being isostructural to the previously reported erbium^[27c]^ and lutetium derivatives.^[27b]^ The Ce−C distances range from 2.501(11) to 2.544(11) Å and are considerably shorter than those in 6‐coordinate **1** (2.6795(19) Å), but match those of [Li(tmeda)_2_][Lu(*t*‐Bu)_4_] (2.32(2)–2.43(2) Å)^[27b]^ and [Li(dme)_3_][Er(*t*‐Bu)_4_] (2.352(6)–2.395(6) Å),^[27c]^ when taking into account the Ln^III^ ion size.

**Figure 4:**
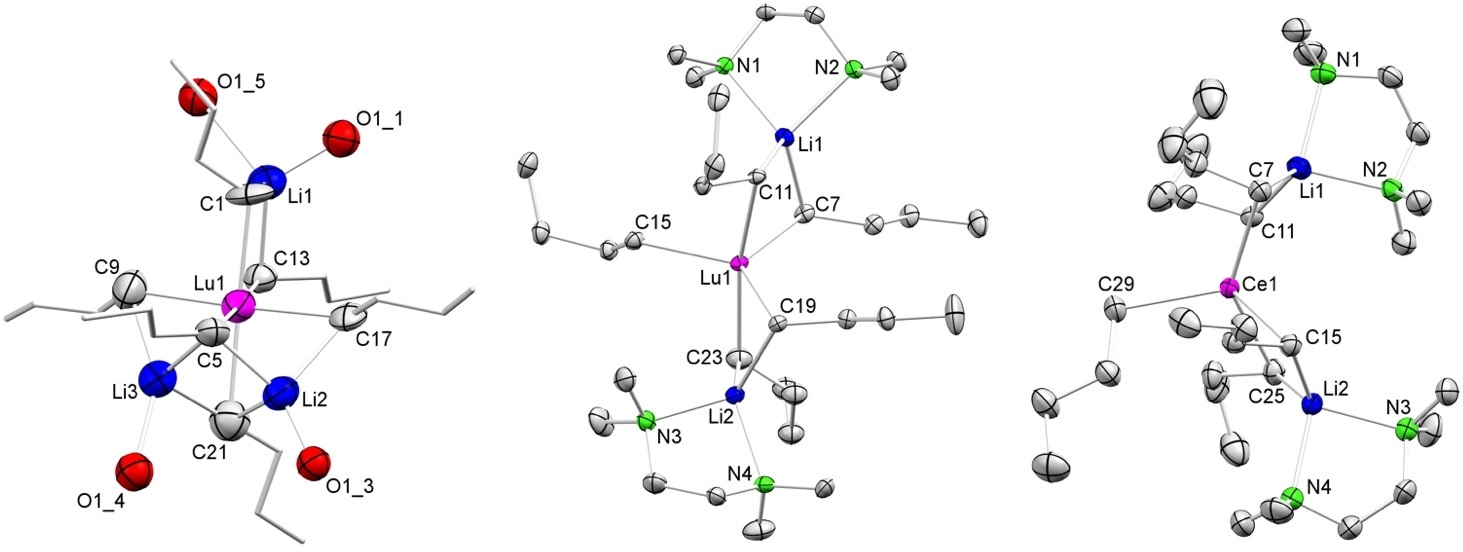
Crystal structures of Li_3_Lu(*n*‐Bu)_6_(thf)_4_ (**3^Lu^**, left) Li_2_Lu(*n*‐Bu)_5_(tmeda)_2_ (**4^Lu^**, middle), and Li_2_Ce(*n*‐Bu)_5_(tmeda)_2_ (**4^Ce^**, right)^[59]^ with atomic displacement ellipsoids set at 30 % probability. Hydrogen atoms, disorders and CH atoms of the THF molecules (**3^Lu^**), and disorders of the *n*‐butyl groups (**4^Lu^**, **4^Ce^**) are omitted for clarity. Selected interatomic distances [Å] and angles [°] for **3^Lu^**: Lu1–C1 2.553(17), Lu1–C5 2.559(18), Lu1–C9 2.46(2), Lu1–C13 2.550(19), Lu1–C17 2.44(2), Lu1–C21 2.58(3), Li1–C1 2.1555(3), Li1–C13 2.3324(3), Li2–C5 2.1928(3), Li2–C17 2.4854(3), Li2–C21 2.4623(3), Li3–C5 2.1307(3), Li3–C9 2.5339(4), Li3–C21 2.3886(3); C1‐Lu1‐C21 176.68(1), C5‐Lu1‐C13 177.55(1), C9‐Lu1‐C17 176.32(1), C1‐Lu1‐C5 88.63(1). **4^Lu^**: Lu1–C7 2.468(2), Lu1–C11 2.5293(18), Lu1–C15 2.3797(19), Lu1–C19 2.4620(18), Lu1–C23 2.522(2), Li1–C7 2.217(4), Li1–C11 2.195(4), Li2–C19 2.224(4), Li2–C23 2.191(4); C7‐Li1‐C11 108.74(1), C19‐Li2‐C23 105.61(1). **4^Ce^**: Ce1–C7 2.657(3), Ce1–C11 2.700(5), Ce1–C15 2.674(3), Ce1–C25 2.664(4), Ce1–C29 2.549(3), Li1–C7 2.185(6), Li1–C11 2.222(8), Li2–C15 2.183(6), Li2–C25 2.205(6); C7‐Li1‐C11 112.1(3), C15‐Li2‐C25 114.3(3).

**Scheme 1:**
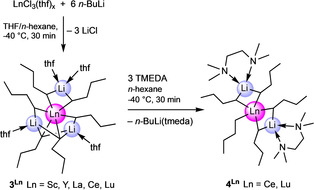
Synthesis of *n*‐butyl complexes **3^Ln^** and **4^Ln^**.

**Synthesis and Solid‐State Structures of*****n*****‐Butyl Derivatives Li_3_Ln(*n*‐Bu)_6_(thf)_4_ and Li_2_Ln(*n*‐Bu)_5_(tmeda)_2_**. Having proven the tamable thermal instability of organocerium complexes **1** and **2** we next tackled the feasibility of the respective *n*‐butyl derivatives. Initially, we attempted to isolate a crystalline cerium‐containing compound from reactions of cerium chloride (thf adduct) with various amounts of *n*‐butyllithium in tetrahydrofuran at low temperatures (−40 °C). Not quite unexpectedly, these endeavors proved to be unsuccessful in the first place. Having in mind the presumably enhanced stability of derivatives of the smaller‐sized rare‐earth metals and to better follow the metathesis reactions via NMR spectroscopy we quickly began to focus on lutetium. Indeed, ate complex Li_3_Lu(*n*‐Bu)_6_(thf)_4_ (**3^Lu^**, Figure 4) could be isolated from the reaction of LuCl_3_(thf)_2_ with 3.3 equivalents of *n*‐BuLi in *n*‐hexane. However, in order to accomplish complex **3^Lu^** six equivalents of *n*‐BuLi must have reacted and associated per LuCl_3_(thf)_2_. It is also notable that four thf molecules have been accommodated in the complex despite the presence of only two in the lutetium chloride precursor. Therefore, THF appeared to be the limiting factor for this reaction. Optimization of the reaction conditions gave the so far best results when anhydrous rare‐earth‐metal chlorides LnCl_3_(thf)_*x*_ (Ln=Sc, Y, La, Ce and Lu; covering the entire Ln^III^ size range) were suspended in a mixture of *n*‐hexane and THF and cooled to −40 °C prior to the addition of *n*‐BuLi. Removal of the volatiles after 30 minutes under reduced pressure, extracting the remaining solid with *n*‐hexane, and concentrating the obtained solution gave crystalline Li_3_Ln(*n*‐Bu)_6_(thf)_4_ (**3^Ln^**, Scheme 1).

Despite our best efforts, we could obtain crystals suitable for XRD analysis only for the lutetium derivative **3^Lu^**. The crystalline material formed for the other “larger” rare‐earth‐metal centers displayed only very poor diffraction behavior. Complex **3^Lu^** features a lutetium center surrounded by six *n*‐butyl ligands, which show distinct linkages to the lithium atoms. Two hydrocarbyl ligands are unsymmetrically bridged by one alkali metal, while the two remaining lithium atoms are unsymmetrically linked to three *n*‐butyl ligands each. The coordination sphere of the three lithium atoms is completed by four THF molecules.

The Lu−C distances range from 2.44(2) to 2.58(3) Å, matching those in Li_3_Lu(CH_3_)_6_(dme)_3_ (2.48(4)–2.57(4) Å),^[23a]^ Lu(AlMe_4_)_3_ (2.455(2)–2.471(2) Å),^[34]^ and Lu(GaMe_4_)_3_ (2.465(2)–2.493(2) Å).^[35]^ All complexes **3^Ln^** were characterized by NMR spectroscopy and elemental analysis. A ^45^Sc NMR experiment for **3^Sc^** showed a broad signal pattern with a main peak centered at +502 ppm, clearly indicating σ‐bonded alkyl species in solution (Figure S17).[^36^, ^37^] For comparison, the ^45^Sc chemical shift of ScMe_3_(thf)_*x*_ was detected at +601.7 ppm.^[36]^ Similarly, the ^1^H‐^89^Y HSQC NMR spectrum of **3^Y^** revealed one low‐field‐shifted ^89^Y resonance at +771 ppm (Figure S22), in accordance with a single organoyttrium species in solution (Y(CH_2_SiMe_3_)_3_(thf)_2_: *δ*(^89^Y)=882.7 ppm).^[38]^ To further corroborate a similar composition of complexes **3^Ln^**, we probed the stabilizing effect of TMEDA for the metal centers cerium and lutetium. Much to our delight, addition of stoichiometric amounts of three equivalents TMEDA prior to the crystallization of the reaction mixtures containing complexes **3^Ln^** afforded complexes Li_2_Ln(*n*‐Bu)_5_(tmeda)_2_ (**4^Ln^**) (Ln=Ce and Lu, Figure 4).

Surprisingly, besides the expected thf/tmeda donor ligand exchange, displacement of one *n*‐BuLi(tmeda) entity took place. Consequently, the rare‐earth‐metal centers in isostructural complexes **4^Ce^** and **4^Lu^** feature only five *n*‐butyl ligands. One of these *n*‐butyl ligands is terminal while of the remaining ones two are bridged by a lithium atom each, which are stabilized by one tmeda donor each. Such *n*‐BuLi(tmeda) displacement is in contrast to the Schumann methyl variants Li_3_Ln(CH_3_)_6_(tmeda)_3_ including complex **1**. A plausible explanation for this is the increased steric bulk/basicity of the *n*‐butyl versus methyl ligands. Complexes **4^Ce^** and **4^Lu^** exhibit distinct Ln−C distances for the terminal and bridging *n*‐butyl ligands (Ce: 2.549(3) Å and 2.657(3)–2.700(5) Å; Lu: 2.3797(19) Å and 2.4620(18)–2.5293(18) Å). Overall, only very limited structural data are available on rare‐earth‐metal *n*‐butyl complexes likely due to the propensity for β‐H elimination. Cyclopentadienyl‐supported derivatives include constrained‐geometry complexes [(η^5^:η^1^‐C_5_Me_4_SiMe_2_N*t*Bu)Y(μ‐*n*‐Bu)]_2_ (Y−C: 2.542(2)/2.544(2) Å) and terminal (η^5^:η^1^‐C_5_Me_4_SiMe_2_N*t*Bu)Y(*n*‐Bu)(dme) (Y−C: 2.435(5) Å)^[39]^ as well as metallocenes [(C_5_H_4_Me)_2_Ln(*n*‐Bu)]_2_ (Ln=Y: 2.551(8)/2.556(11)/2.587(13) Å, Dy: 2.536(18)/2.591(18) Å).^[40]^ Because cerium and lutetium form the same type of complex for **4^Ln^**, and based on other analytical data, monolanthanide derivatives of general formula Li_3_Ln(*n*‐Bu)_6_(thf)_4_ are also proposed for the remaining complexes **3^Ln^**.

At this point we were once more challenged by the question why these reactions would lead to the isolation of ate complexes Li_3_Ln(*n*‐Bu)_6_(thf)_4_ rather than the envisaged “Ln(*n*‐Bu)_*x*_Cl_3−*x*_(thf)_*y*_” (*x=*1–3). When starting from LnCl_3_(thf)_*x*_, which is very poorly soluble in THF, the simplest explanation would be that treatment of Lewis‐acidic rare‐earth‐metal chloride species with increasing amounts of the strong nucleophile *n*‐BuLi enhances its solubility, and is therefore more likely to react faster in consecutive reactions with *n*‐BuLi. The formation of isolable ate complexes Li_3_Ln(*n*‐Bu)_6_(thf)_4_ is further driven by the relatively small size of the *n*‐butyl ligand and by switching the solvent from coordinating (THF) to non‐coordinating (*n*‐hexane). Predominant ate complexation was also observed for Schumann's methyl complexes Li_3_LnMe_6_(thf)_*x*_ ^[22]^ or diisopropylamido derivatives LiLn(N*i*Pr_2_)_4_(thf)_*x*_.^[41]^ Crucially, independent of the applied LuCl_3_(thf)_*x*_/*n*‐BuLi stoichiometry, complex **3^Lu^** could be crystallized as the exclusive Lu^III^‐containing species, upon separation of the solution from unreacted rare‐earth‐metal halide, its evaporation to dryness, and extraction of the residue with *n*‐hexane (XRD unit‐cell check and ^1^H NMR spectroscopy indicated repeatedly formation of **3^Lu^**). This finding suggested that the typical reaction (preformation) conditions for a lutetium‐derived Imamoto reagent, LuCl_3_/*n*‐BuLi/THF/−78 °C/30 min should form Li_3_Lu(*n*‐Bu)_6_(thf)_*x*_ as the dominant initial lutetium species in solution. Since any persisting equilibria in solution would impact the reactivity of the Imamoto alkylation reagent we next took a closer look at the solution behavior of bimetallic Li_3_Ln(*n*‐Bu)_6_(thf)_4_ and the binary system LnCl_3_(thf)_*x*_/*n*‐BuLi (Ln=Ce, Lu).

**Solution Behavior of*****n*****‐Butyl Complexes 3^Ln^ and 4^Ln^ Probed by NMR spectroscopy**. All crystallized *n*‐butyl complexes are stable when stored as solids at −40 °C. However, when dissolved in any solvent, complexes **3^La^**, **3^Ce^** and **4^Ce^** had fully decomposed after 24 h. In contrast, but not unexpectedly, the complexes of the smaller‐sized rare‐earth metals are stable in solution at −40 °C for up to one week. Warming complexes **3^Ln^** and **4^Ln^** to ambient temperature, decomposition was perceived visually within one hour (in both solution and solid state). To further determine the thermal stability of our complexes, variable‐temperature (VT) NMR spectra were measured (Figures S18, S23, S26, S36 and S42). Amazingly, β‐H elimination and 1‐butene formation was observed only from +30 °C onwards, being considerably more pronounced for **3^La^** and **3^Ce^** than for the respective complexes of the smaller‐sized rare‐earth metals. Because the heating was performed in 10 degree increments starting at −40 °C and held at each temperature for 15 minutes before measurement, it can be concluded that these complexes are relatively stable for a short amount of time even at ambient temperature.^[33]^

**Figure 5:**
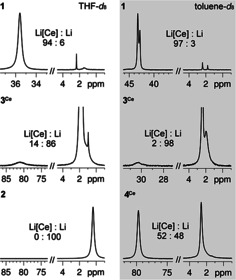
^7^Li NMR spectra (194.37 MHz, 233 K) of complexes **1**, **2**, **3^Ce^**, and **4^Ce^**, recorded in [D_8_]THF or [D_8_]toluene.

Crucially, both the ^1^H and ^7^Li NMR spectra of complexes **3^Ln^** and **4^Ln^** revealed that in solution a considerable portion of *n*‐BuLi gets displaced from the rare‐earth‐metal center. The degree of dissociation is highly dependent on the solvent and the rare‐earth metal. In general, *n*‐BuLi dissociation is more pronounced in THF than in toluene. Ate complexes **3^Ln^** of the smaller‐sized rare‐earth metals yttrium, lutetium, and scandium are quite stable in toluene solution displaying minor *n*‐BuLi dissociation of ca. 1 %, 4 %, and 8 % respectively (Figures S14/S19/S37). On the other hand, *n*‐BuLi dissociation prevails for **3^Ce^** and **3^La^** (ca. 90 %). Interestingly, the presence of tmeda as a donor ligand in complexes **4^Ln^** can either counteract or enforce *n*‐BuLi separation (**3^Ce^**/**4^Ce^**: >98 %/46 % versus **3^Lu^**/**4^Lu^**: 4 %/20 %). For the organocerium(III) complexes, *n*‐BuLi ate complexation is easily detectable by paramagnetically shifted ^7^Li resonances. Figure 5 depicts the ^7^Li NMR spectra of complexes **1**, **2**, **3^Ce^**, and **4^Ce^** both in [D_8_]THF and [D_8_]toluene, clearly revealing a) the great stability of the hexamethylate complex **1**, b) the persistence of ion‐separated *tert*‐butyl complex **2** also in solution, and c) the beneficial effect of tmeda (versus thf) donor ligands for intramolecular ate‐complex stabilization. The ^7^Li NMR spectrum of **4^Ce^** suggests a clean separation into [LiCe(*n*‐Bu)_4_(tmeda)] and *n*‐BuLi (signal ratio 1:1).

These findings have important implications for the composition of the “active” *n*‐BuLi‐derived Imamoto reagent. When used as a 1:1 mixture of CeCl_3_(thf) and *n*‐BuLi in THF at temperatures of −35 °C, the formation of ate complex Li_3_Ce(*n*‐Bu)_6_(thf)_4_ (**3^Ce^**) seems highly unfavored. This can be concluded from the ^7^Li NMR spectrum of **3^Ce^**, which does indicate only a small portion of lithium and paramagnetic cerium(III) in close proximity (*δ*_Li_=81.5 ppm). An equimolar mixture of CeCl_3_(thf) and *n*‐BuLi formed in situ in [D_8_]THF at −45 °C did not reveal any paramagnetically shifted ^7^Li NMR resonance (Figure S33). The latter seems to appear only at ratios <1:3. For comparison, the intramolecular ate complex LiCe[N(SiHMe_2_)_2_]_4_(thf) (in C_6_D_6_/1,2‐difluorobenzene)^[42]^ and tmeda‐adduct **4^Ce^** (in [D_8_]THF) display ^7^Li chemical shifts of 84.4 and 81.3 ppm. The ^1^H NMR spectrum in [D_8_]THF of **3^La^** featuring the similarly sized lanthanum center lends further support to this assumption, as the *n*‐butyl resonances appear in an approximate 1:1 ratio (Figure S24). Moreover, 1:1 mixtures of CeCl_3_(thf) and *n*‐BuLi, obtained in THF at −45 °C contain a substantial amount (40–50 %) of unreacted CeCl_3_(thf) upon preformation for 30 minutes. Incomplete transmetalation in CeCl_3_/RLi mixtures has been pointed out previously at various occasions.[^12a^, ^21^] Therefore, unlike the smaller rare‐earth metals, which favor intramolecular ate complexation even in solution, the organocerium species prevailing under Imamoto conditions are most likely “Ce(*n*‐Bu)_3_(thf)_*x*_” or solvent‐separated ion pairs like “[Ce(*n*‐Bu)_4_(thf)_*y*_][Li(thf)_4_]”, similar to *tert*‐butyl complex **2**. The formation of heteroleptic species “Ce(*n*‐Bu)_*x*_Cl_*y*_(thf)_*z*_” (*x=*1,2; *x*+*y=*3) seems very unlikely due to the persistence of ligand redistribution forming homoleptic complexes and/or the favorable occurrence of β‐H elimination. Non‐ate mixed hydrocarbyl/halide Ln^III^ complexes have been structurally authenticated for phenyl and benzyl derivatives but have remained elusive for alkyl ligands capable of β‐H elimination. Representative examples include (C_6_H_5_)GdCl_2_(THF)_4_,^[43]^ Ln(CH_2_Ph)_2_I(thf)_3_ (Ln=Y, Er),^[44]^ and ion‐separated [YMeI(py)_5_][I].^[24]^

**Figure 6:**
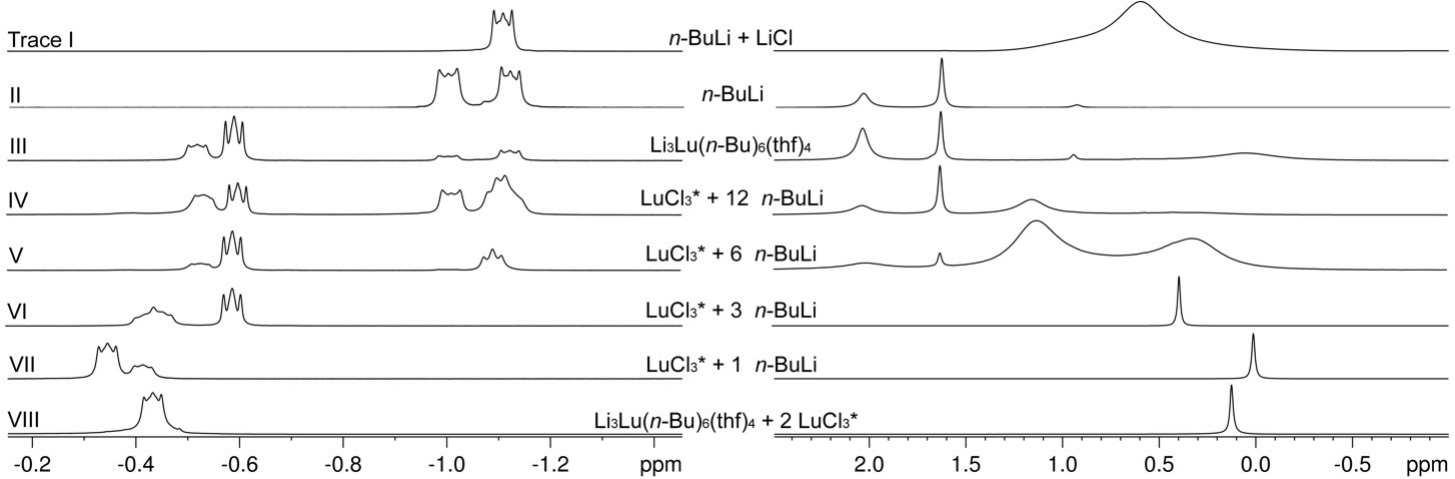
^1^H NMR (500.13 MHz, [D_8_]THF, 193 K) (left) and ^7^Li NMR spectra (194.37 MHz, [D_8_]THF, 193 K) (right) of LuCl_3_(thf)_2_ with *x* *n*‐BuLi (*x=*1, 3, 6, 12) compared to **3^Lu^**, **3^Lu^** +2 LuCl_3_(thf)_2_, *n*‐BuLi, and *n*‐BuLi+LiCl.

The dissociation behavior was further investigated in a series of NMR experiments (Figure 6), comparing complex **3^Lu^** to the reactions of LuCl_3_(thf)_2_ with various amounts of *n*‐BuLi, and *n*‐BuLi itself. The ^1^H NMR spectrum of crystalline ate complex Li_3_Lu(*n*‐Bu)_6_(thf)_4_ (**3^Lu^**) shows two signal sets for metal‐bonded CH_2_ groups (Figure 6, trace III/left). The Lu−CH_2_ moieties resonate at about −0.5 ppm, and hence are significantly shifted to lower field compared to the characteristic pattern of *n*‐BuLi at −1.0 to −1.5 ppm (cf. trace II/left).[^45^, ^46^] The two signal sets are clearly indicative of *n*‐BuLi dissociation in THF solution (Imamoto conditions). As in case of *n*‐BuLi, the two distinct signals for the lutetium‐bounded *n*‐Bu ligands might represent lutetium complexes of distinct aggregation “Li_*x*_Lu(*n*‐Bu)_3+*x*_(thf)_*y*_”(*x=*0–3). The dissociation of *n*‐BuLi in **3^Lu^** is also corroborated by the ^7^Li NMR spectrum (trace III/right) showing the characteristic pattern of *n*‐BuLi[^47^, ^48^] and a very broad signal at 0 ppm, indicative of Lu‐*n*‐Bu‐Li moieties and rapid *n*‐BuLi exchange. Interestingly, when examining in situ formed solutions of LuCl_3_(thf)_2_/*n*‐BuLi (traces IV‐VII), free *n*‐BuLi was observed only when more than three equivalents of *n*‐BuLi were used per lutetium. This is supported by the respective ^7^Li NMR spectra, which also suggest the formation of free LiCl in case of <3 equivalents of *n*‐BuLi. For better comparison the ^1^H and ^7^Li spectra of *n*‐BuLi‐LiCl mixtures are shown (trace I). Moreover, addition of LuCl_3_(thf)_2_ to ate complex Li_3_Lu(*n*‐Bu)_6_(thf)_4_ (**3^Lu^**) results in complete consumption of free *n*‐BuLi and formation of LiCl (trace VIII). This implies that dissociated *n*‐BuLi engages in “normal” ligand exchange with added LuCl_3_(thf)_2_. However, when the solvent is removed the in situ formed complexes scramble to the complex **3^Lu^**, LuCl_3_ and LiCl (upon crystallization from *n*‐hexane).

Based on these observations, we can hypothesize about the following scenario. The initial intermediate/transient product of the LuCl_3_(thf)_2_/*x* *n*‐BuLi reaction is certainly “Lu(*n*‐Bu)Cl_2_”. However, as soon as this heteroleptic complex is formed, its better solubility in THF (compared to LuCl_3_) will imply a more rapid reaction (compared to LuCl_3_) with the remaining *n*‐BuLi in solution. Thus, ate complexes of the type “Li_*x*_Lu(*n*‐Bu)_3+*x*_(thf)_*y*_”, and ultimately but not exclusively Li_3_Lu(*n*‐Bu)_6_(thf)_4_ (**3^Lu^**), represent the dominant rare‐earth‐metal species in solution. As revealed by NMR spectroscopies, **3^Lu^** is labile in solution and engages in a dissociation equilibrium with *n*‐BuLi; the displaced *n*‐BuLi should react further with LuCl_3_. Therefore, likely reaction products depending on the LuCl_3_(thf)_2_/*n*‐BuLi ratio are “Lu(*n*‐Bu)_3_” and “Li_*x*_Lu(*n*‐Bu)_3+*x*_(thf)_*y*_” with *x*≤2 for a ratio of 1:3 and smaller, and species “Lu(*n*‐Bu)_3_” and “Li_*x*_Lu(*n*‐Bu)_3+*x*_(thf)_*y*_” with *x* ranging from 1 to 3, for a ratio of larger than 1:3. A shortage of THF solvent via extraction of the reaction products into *n*‐hexane leads to ate complex Li_3_Lu(*n*‐Bu)_6_(thf)_4_ (**3^Lu^**) as the only isolable (crystalline) species. Performing the reactions for longer time periods under otherwise identical conditions resulted in extensive decomposition.

**Figure 7:**
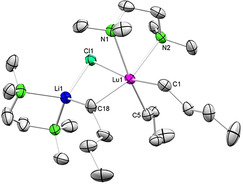
Crystal structure of LiLu(*n*‐Bu)_3_Cl(tmeda)_2_ (**5**) with atomic displacement ellipsoids set at 50 % probability.^[59]^ Hydrogen atoms and disorders of the *n*‐butyl groups are omitted for clarity. Selected interatomic distances [Å] and angles [°] for **5**: Lu1–C1 2.421(3), Lu1–C5 2.367(3), Lu1–C18 2.447(3), Lu1–Cl1 2.7191(9), Lu1–N1 2.635(3), Lu1–N2 2.540(2), Li1–C18 2.287(6), Li1–Cl1 2.308(5); C18‐Lu1‐Cl1 89.26(9), C18‐Li1‐Cl1 104.6(2).

**Scheme 2:**
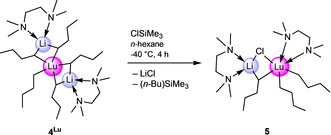
Reactivity of lutetium complex **4^Lu^** with ClSiMe_3_: “lithium depletion”.

**Solution Behavior of*****n*****‐Butyl Complexes 3^Ln^ and 4^Ln^ Probed by Derivatization Reactions**. The preferred dissociation of *n*‐BuLi from ate complexes Li_3_Ln(*n*‐Bu)_6_(thf)_4_ (**3^Ln^**) of the larger‐sized rare‐earth metals was further revealed by the reaction of crystalline **3^Ce^** with LuCl_3_(thf)_2_ in THF, affording **3^Lu^** in low crystalline yields of 23 % (XRD unit‐cell check). Interestingly, the dissociated *n*‐BuLi seems to exert a stabilizing effect on the organocerium species, since the solution turned brown rather quickly, upon addition of LuCl_3_(thf)_2_. In order to enforce the formation of *n*‐BuLi‐depleted “Ln(*n*‐Bu)_3_” we searched for reactions which would possibly convert any dissociated *n*‐BuLi selectively and ideally into products not affecting the isolation of putative “Ln(*n*‐Bu)_3_”. Luckily, such a reaction path could be observed for the treatment of **4^Lu^** with trimethylsilyl chloride. The reaction was slow at −40 °C, but it produced a minor amount of crystalline LiLu(*n*‐Bu)_3_Cl(tmeda)_2_ (**5**, Scheme 2, Figure 7). On various other occasions, when trying to precipitate LiCl or extract “Ln(*n*‐Bu)_3_” from the LiCl‐containing residue, only progressive decomposition could be observed. Again, depending on the size of the rare‐earth‐metal center, complete decomposition took place in a few minutes (“Ce(*n*‐Bu)_3_”) or several hours (“Y(*n*‐Bu)_3_”). This behavior clearly shows the stabilizing effect of LiCl in these reactions making THF an ideal solvent.

In the solid state, the lutetium atom of complex **5** adopts a distorted octahedral coordination geometry, involving three *n*‐butyl ligands, one chlorido ligand and a chelating tmeda molecule. The chlorido and one *n*‐butyl ligand bridge to the lithium atom forming a four‐membered ring. The coordination of the lithium atom is completed by the second tmeda molecule. Noteworthy, in the course of this reaction one tmeda ligand was transferred from lithium to lutetium. Striking are the distinct Lu−C distances of the terminal *n*‐butyl ligands of 2.367(3) and 2.421(3) Å. While the shorter distance approximately matches the terminal one of complex **4^Lu^** (2.3797(19) Å), the longer distance is almost as long as that of the bridging *n*‐Bu ligand (2.447(3) Å). This might indicate a *trans* influence of the weakly coordinated chlorido ligand. In accordance with the crystal structure, the ^1^H NMR spectrum of **5** displays two distinct signal sets for the *n*‐butyl ligands in a 2:1 ratio (Figure S48).

**Scheme 3:**
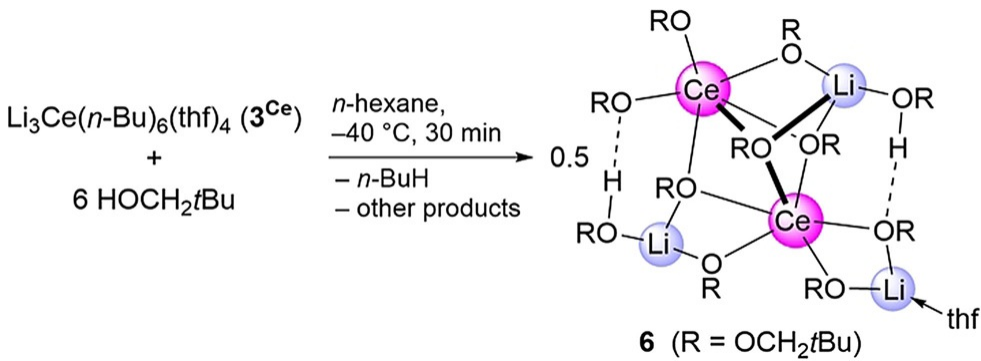
Alcoholysis of cerium complex **3^Ce^** with neopentanol.

Unsurprisingly, the *n*‐butyl ligands of complexes **3^Ln^** get easily protonated in the presence of alcoholic substrates. As an example, treatment of Li_3_Ce(*n*‐Bu)_6_(thf)_4_ (**3^Ce^**) with six equiv of neopentanol resulted in the crystallization of the heterobimetallic cluster Li_3_Ce_2_(OCH_2_ *t*Bu)_9_(HOCH_2_ *t*Bu)_2_(thf) (**6**, Scheme 3, Figure S1). On the basis of the crystal structure and Ce−O distances, the connectivity of **6** can be assigned as Li_3_Ce_2_(μ_3_‐OCH_2_ *t*Bu)_3_(μ_2_‐OCH_2_ *t*Bu)_4_(OCH_2_ *t*Bu)_2_(HOCH_2_ *t*Bu)_2_(thf). The μ_2_‐bridging neopentoxy ligands involve one lithium and cerium each, the μ_3_‐bridging ones connect two cerium atoms with one lithium, and the terminal ones are coordinated to one cerium center (Ce1). The coordination sphere of the lithium atoms is saturated with one thf and two alcohol donor molecules. The alcohol donors of **6** engage in hydrogen bonding with one terminal (O3) and one μ_2_‐bridging neopentoxy ligand (O10). Due to the distinct coordination modes, the Ce−O distances span a wide range of 2.196(2) to 2.608(2) Å, but match those of other cerous alkoxides.^[49]^ Overall, the solid‐state structure of **6** features a completely asymmetric complex with all five metal centers displaying different coordination environments. A similar structure was reported for the yttrium neopentoxide Li_3_Y_2_(μ_3_‐OCH_2_ *t*Bu)(μ_3_‐HOCH_2_ *t*Bu)(μ_2_‐OCH_2_ *t*Bu)_5_(OCH_2_ *t*Bu)_3_(HOCH_2_ *t*Bu)_2_.^[50]^ The formation and structural characterization of Li_3_Ce_2_ complex **6** clearly reflects lithium depletion compared to the Li_3_Ce precursor **3^Ce^**, and pictures the intricacy of **3^Ce^** in solution. Complex **6** shows intricate solution behavior itself as evidenced by at least six signals in the ^7^Li NMR spectrum, including paramagnetically shifted ones (Figure S52).

**Reactivity of*****n*****‐Butyl Complexes 3^Ln^ toward an Enolizable Ketone**. In this study, 1,3‐diphenylpropan‐2‐one was employed as a test molecule (Table 1), since its selective conversion into the respective tertiary alcohol has been already shown in the original work by Imamoto.^[7]^ Back then the following protocols were applied: [Ce, I_2_, THF, 0 °C (CeI_3_ formed in situ)/*n*‐BuLi (1 equiv), −65 °C, 30 min/ketone, −65 °C, 3 h] and [“CeCl_3_” (later determined as [CeCl_3_(H_2_O)]_*n*_), *n*‐BuLi (1 equiv), THF, −78 °C, 1 h/ketone, −65 °C, 3 h], yielding the alcohol in 98 % and 96 %, respectively.

**Table 1:** Overview of the ketone reduction. 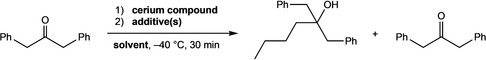

Entry^[a]^	Compound	Equiv ketone	Solvent	Additives	Yield alcohol [%]	Recovered ketone [%]
1	CeCl_3_(thf)	0.77	THF	1 *n*‐BuLi	99	0
2	CeCl_3_(thf)	6	THF	6 *n*‐BuLi	77	20
3	**3^Ce^**	6	THF	–	70	13
4	**3^Ce^**	1	THF	–	88	3
5	**3^Ce^**	6	Et_2_O	–	54^[b]^	46^[b]^
6	**3^Ce^**	3	Et_2_O	–	78	15
7	**3^Ce^**	1	Et_2_O	–	89	10
8	**3^Ce^**	6	toluene	–	76	11
9	**3^Ce^**	6	THF	3 LiCl	74	16
10	**3^Ce^**	6	THF	5 CeCl_3_(thf)	89	8
11	**3^Ce^**	6	THF	5 “Ce turbo chloride” to ketone	80	14
12	**3^Ce^**	6	THF	5 “Ce turbo chloride” after ketone	90	1
13	**3^Ce^**	6	THF	5 Sc(OTf)_3_ after ketone	62^[b]^	38^[b]^
14	**3^Ce^**	6	THF	5 AlCl_3_ after ketone	79	21
15	**3^Ce^**	6	THF	3 TMEDA	73	16
16	**3^Lu^**	6	THF	–	74	23
17	**3^Lu^**	6	THF	3 TMEDA	44	56
18	**3^Y^**	6	THF	–	75	12
19^[c]^	**3^Lu^**	6	THF	–	62	38
20	–	1	THF	1 *n*‐BuLi	50	50

[a] 0 °C. [b] Ratios determined by NMR spectroscopy. [c] Ambient temperature.

In our hands, the slightly modified version [CeCl_3_(thf), *n*‐BuLi (1 equivalent), THF, −35 °C, 30 min/ketone, −35 °C, 30 min] gave 99 % of alcohol (entry 1), clearly documenting the efficiency of the Imamoto transformation. The importance of using CeCl_3_/*n*‐BuLi in a 1:1 stoichiometry is revealed by the reactivity of crystalline or in situ formed complex **3^Ce^** (entries 2 and 3). The yield of alcohol was significantly decreased at the expense of ketone enolization caused by dissociated *n*‐BuLi (for the exclusive reaction behavior of *n*‐BuLi, see entry 20). Conducting the reaction in diethyl ether also gave the best results when CeCl_3_(thf)/*n*‐BuLi was used in a 1:1 stoichiometry (entries 5–7). Surprisingly, complex **3^Ce^** performed similarly in THF and toluene (entry 8).

**Figure 8:**
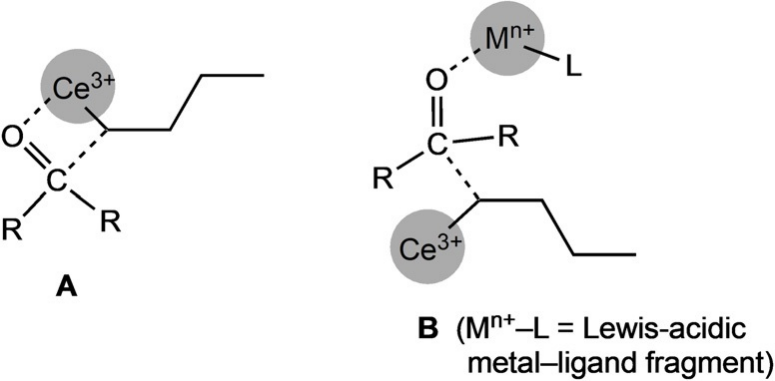
Proposed “intramolecular” reaction involving a four‐membered transition state of the ketone reduction (**A**) rather than a multimolecular reaction path like **B**.

To comprehend why the alcohol yield is lower in case of **3^Ce^** than for CeCl_3_(thf)/*n*‐BuLi (ratio 1:1, entries 3 and 4 versus 1), various additives were tested. While the addition of LiCl did not affect the reaction outcome (entry 9), additional CeCl_3_(thf) did markedly increase the alcohol yield (entry 10). This effect was even significantly enhanced if “cerium turbo chloride” was added to the organocerium compound subsequently to the ketone (entry 12). “Cerium turbo chloride” is the combination of CeCl_3_ with two equivalents of LiCl and completely soluble in THF. It was prepared according to the method reported by the Knochel group.^[51]^ As revealed by NMR spectroscopy in case of lutetium (Figure 6, trace VIII), dissociated *n*‐BuLi will react with added LnCl_3_ along with ligand scrambling and provide for the further supply of cerium‐bonded *n*‐butyl. Therefore the limited performance of complex **3^Ce^** with 6 equivalents of ketone results from progressing *n*‐BuLi dissociation and its changed (reduced/non‐selective) reactivity toward the ketone. For ate complexes **3^Ln^** of the smaller‐sized yttrium and lutetium, which are more stable in solution, the mediocre performance in the 6‐equivalent reaction might be attributable to the formation of sterically demanding alkoxy ligands in putative “Li_3_Ln[OC(CH_2_Ph)_2_ *n*‐Bu]_6_”. Overall, any effect of the rare‐earth‐metal size is not apparent since ate complexes **3^Y^** and **3^Lu^** showed a performance very similar to that of the cerium congener **3^Ce^** (entries 16 and 18). The importance of performing the transformation in the presence of rare‐earth‐metal chlorides was also revealed when employing scandium triflate Sc(OTf)_3_ or AlCl_3_ as Lewis acids instead of additional CeCl_3_ (entry 13 and 14). Both reactions led to decreased alcohol yields and increased ketone recovery (via enolization). This and the distinct outcome of the ketone transformation when changing the order of ketone and “cerium turbo chloride” (entries 11 and 12) suggest an intramolecular ketone activation/alkylation scenario akin to a four‐membered transition state (1,2‐addition), rather than the participation of several metal complexes (Figure 8, **A** and **B**). Moreover, favorable ketone coordination/adduct formation[^52^, ^53^] and alkoxide formation^[54]^ have been shown previously.

The impact of TMEDA was examined for in situ formed **4^Ce^** and **4^Lu^** (entries 15 and 17), indicating a pronounced *n*‐BuLi(tmeda) dissociation only in case of the lutetium reaction. Finally, performing the ketone transformation with **3^Lu^** at ambient temperature resulted not unexpectedly in a significant drop of alcohol formation, but also documents the relative Ln^III^‐size‐dependent stability of the complexes at ambient temperature for a short time.

Finally, the isolation and crystallization of lithium alkoxide co‐products gave further insights into the Imamoto alkylation scenario. Prolonging the reaction time of the “incomplete” transformation of six equivalents of 1,3‐diphenylpropan‐2‐one with **3^Ce^** (Table 1, entry 3) to three weeks led to the crystallization of the enolization product lithium 1,3‐diphenylprop‐1‐en‐2‐ate Li_4_[OC(=CHPh)CH_2_Ph]_4_(thf)_4_ (**7**, Figure S2). The dissociated, significantly more stable *n*‐BuLi (compared to **3^Ce^**) acts as a base, deprotonating unreacted ketone at the α‐position of the carbonyl moiety. This competitive reaction path reflects the main part of the recovered ketone, as listed in Table 1, since aqueous work‐up will involve keto–enol tautomerism. Complex **7** features a common structural motif in lithium alkoxide complexes[^55^, ^56^] with the lithium and alkoxy oxygen atoms occupying alternating positions of a cube. The lithium atoms are saturated with one THF molecule each.

Preliminary tests with CeCl_3_(thf)/*n*‐BuLi mixtures in the molar ratio 1:3 and acetone as the substrate led to the crystallization of the lithium alkoxide Li_8_[OCMe_2_(*n*‐Bu)]_6_Cl_2_(thf)_6_ (**8**, Figure S3). The incorporation of LiCl into the cluster core unambiguously documents that it is an integral part of the reagent solution. This is also revealed by the ^7^Li NMR spectra depicted in Figure 6 (right, traces VI‐VIII). Dissolved LiCl might also associate with the organocerium species (as detected for complex **5**) thus exerting a stabilizing effect.

**Scheme 4:**
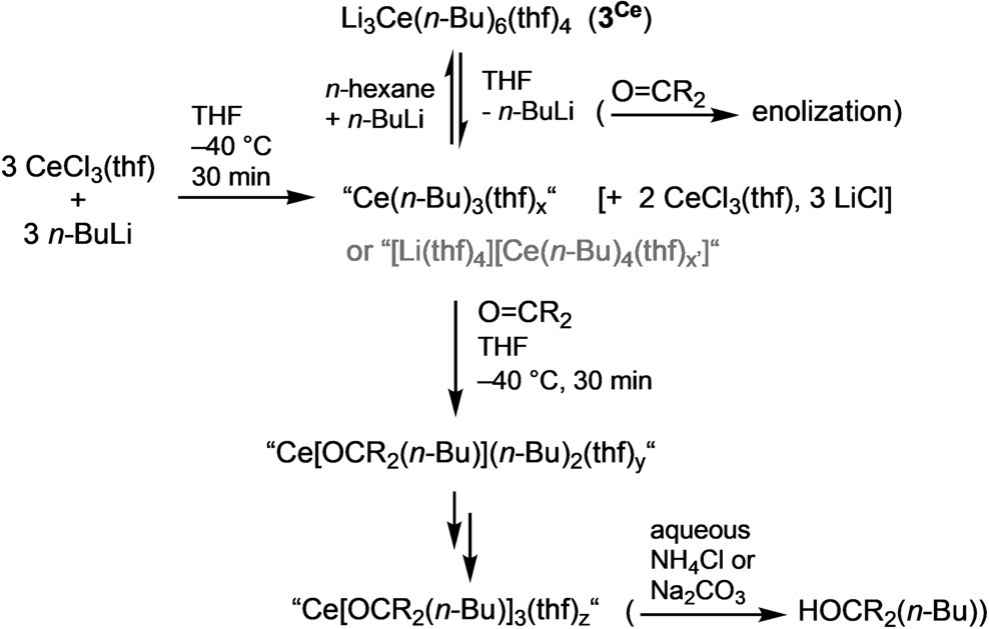
The Imamoto organocerium reagent in action.

**Proposed Formation and Reactivity of the Imamoto Organocerium Reagent (Scheme **4**)**. Equimolar amounts of CeCl_3_ and *n*‐BuLi, when combined in THF at low temperatures (−40 °C), afford the cerous *n*‐butyl complex “Ce(*n*‐Bu)_3_(thf)_*x*_” or solvent‐separated ion pairs like “[Ce(*n*‐Bu)_4_(thf)_*y*_][Li(thf)_4_]”, as suggested by a) ^1^H and ^7^Li NMR spectroscopies and b) considerable amounts of unreacted cerium chloride. In particular, ^7^Li NMR spectroscopy indicates complete transmetalation (complete consumption of *n*‐BuLi) and the absence of adjacent cerium and lithium centers.

Unfortunately, single‐crystalline products were not accessible. The formation of such species is further corroborated by the solution behavior of pre‐isolated crystalline ate complex Li_3_Ce(*n*‐Bu)_6_(thf)_4_ (**3^Ce^**), displaying extensive *n*‐BuLi dissociation in THF solution. Note that the product obtained from the reaction of CeCl_3_⋅2 LiCl (cerium turbo chloride) with three equivalents of *n*‐BuLi in THF at −30 °C was previously analyzed as “*n*‐Bu_3_Ce” by Raman spectroscopy (absence of significant CeCl_3_ and *n*‐BuLi Raman lines).^[15]^ Activation of the carbonylic substrate and its transformation (here reduction to the alkoxy moiety) takes place at the same cerium center, and can proceed three times. Such 1,2‐addition reactions have been proven for yttrium methyls [YMe_3_]_*n*_,^[57]^ [YMe_2_(thf)_5_][BPh_4_], and [YMe(thf)_6_][BPh_4_]_2_,^[24]^ as well as neosilyls [Y(CH_2_SiMe_3_)_2_(thf)_4_][A] (A=BPh_4_, Al(CH_2_SiMe_3_)_4_) and [Y(CH_2_SiMe_3_)(thf)_5_][BPh_4_]_2_ ^[58]^ employing fluorenone and benzophenone. Aqueous work‐up will lead to the alcoholic product and a recyclable inorganic cerium compound.

## Conclusion

Seamless low‐temperature synthesis and crystallization techniques give access to isolable organocerium complexes Li_3_Ce(CH_3_)_6_(tmeda)_3_, [Li(thf)_4_][Ce(*t*‐Bu]_4_], Li_3_Ce(*n*‐Bu)_6_(thf)_4_, and Li_2_Ce(*n*‐Bu)_5_(tmeda)_2_. ^1^H/^7^Li NMR spectroscopic studies on in situ formed solutions of equimolar mixtures of CeCl_3_(thf) and *n*‐BuLi in THF at −35 °C suggest non‐isolable “Ce(*n*‐Bu)_3_(thf)_*x*_” or solvent‐separated ion pairs like “[Li(thf)_4_][Ce(*n*‐Bu)_4_(thf)_*y*_]” as effective organocerium species in respective Imamoto‐type alkylation reactions of carbonylic substrates. This hypothesis is corroborated by the solution behavior of ate complex Li_3_Ce(*n*‐Bu)_6_(thf)_4_, which displays extensive displacement of *n*‐BuLi. As revealed for the benchmark substrate molecule 1,3‐diphenylpropan‐2‐one, the prevailing dissociation of *n*‐BuLi results in decreased regioselectivity of the ketone/alcohol transformation. In contrast, ate complexes of the type Li_3_Ce(CH_3_)_6_(thf)_*x*_ could be likely species in methyllithium derived Imamoto‐type reagents as revealed by the high stability of Li_3_Ce(CH_3_)_6_(tmeda)_3_ in THF solution. Not unsurprisingly, the effective composition of such CeCl_3_/RLi reagents is highly dependent on the rare‐earth metal, the hydrocarbyl group, and the solvent. Finally, the presence of THF‐soluble co‐product LiCl in in situ formed reagents is proposed to adopt an active role by exerting a stabilizing effect on the organocerium species. Compound LiLu(*n*‐Bu)_3_Cl(tmeda)_2_ features a structural snapshot of the likely involvement of LiCl in the soluble part of Imamoto‐type transformations.

It is a fact that a large number of organometallics‐promoted organic transformations (including the in situ formation of the multi‐component organometallic reagent) is routinely performed at low temperatures (−78→0 °C). It can be safely assumed that the application of state‐of‐the‐art cold‐chain techniques will continue to promote a better understanding of the reagent's formation, composition, and effectiveness, ultimately leading to both optimized reagents and conditions for substrate conversion.

## Conflict of interest

The authors declare no conflict of interest.

## Supporting information

As a service to our authors and readers, this journal provides supporting information supplied by the authors. Such materials are peer reviewed and may be re‐organized for online delivery, but are not copy‐edited or typeset. Technical support issues arising from supporting information (other than missing files) should be addressed to the authors.

Supplementary
