# Supplementary material for: CeCl3/n‐BuLi: Unraveling Imamoto's Organocerium Reagent
Source: Angew Chem Int Ed Engl. 2021 Jun 8;60(28):15622–31. doi: 10.1002/anie.202103889 (PMC8362106; doi:10.1002/anie.202103889)
Supplement: Supplementary file 1 — Supplementary [file ANIE-60-15622-s001.pdf]

Angewandte Chemie
Eine Zeitschrift der Gesellschaft Deutscher Chemiker

Supporting Information

$CeCl_3$/*n*-BuLi: Unraveling Imamoto's Organocerium Reagent

*Tassilo Berger, Jakob Lebon, Cäcilia Maichle-Mössmer, and Reiner Anwander**

anie_202103889_sm_miscellaneous_information.pdf
anie_202103889_sm_cif.zip

Table of Contents

Experimental Section S6

General Considerations. S6

[$Li_3CeMe_6(tmeda)_3$] (**1**). S6

[$Li(thf)_4$][$Ce(t\text{-}Bu)_4$] (**2**). S7

General Procedure for the Synthesis of $Li_3Ln(n\text{-}Bu)_6(thf)_4$ (**3**$^{\mathbf{Ln}}$, Ln = Sc, Y, La, Ce, Lu). S7

$Li_3Sc(n\text{-}Bu)_6(thf)_4$ (**3**$^{\mathbf{Sc}}$). S7

$Li_3Y(n\text{-}Bu)_6(thf)_4$ (**3**$^{\mathbf{Y}}$). S8

$Li_3La(n\text{-}Bu)_6(thf)_4$ (**3**$^{\mathbf{La}}$). S8

$Li_3Ce(n\text{-}Bu)_6(thf)_4$ (**3**$^{\mathbf{Ce}}$). S8

$Li_3Lu(n\text{-}Bu)_6(thf)_4$ (3^{Lu}). S8

Equimolar reaction of $CeCl_3(thf)$ with *n*-BuLi. S8

Synthesis of **3**$^{\mathbf{Lu}}$ for XRD analysis. S9

Synthesis of **3**$^{\mathbf{Lu}}$ from "lutetium turbo chloride" S9

Synthesis of **3**$^{\mathbf{Lu}}$ from **3**$^{\mathbf{Ce}}$. S9

General Procedure for the Synthesis of $Li_2Ln(n\text{-}Bu)_5(tmeda)_2$ (**4**$^{\mathbf{Ln}}$, Ln = Ce, Lu). S9

$Li_2Ce(n\text{-}Bu)_5(tmeda)_2$ (**4**$^{\mathbf{Ce}}$). S9

$Li_2Lu(n\text{-}Bu)_5(tmeda)_2$ (**4**$^{\mathbf{Lu}}$). S9

$LiLu(n\text{-}Bu)_3Cl(tmeda)_2$ (**5**). S10

Synthesis of $Li_3Ce_2(ONep)_9(HONep)_2(thf)$ (**6**). S10

Synthesis of $Li_4[OC(=CHPh)(CH_2Ph)]_4(thf)_4$ (**7**). S10

Synthesis of $Li_8[OCMe_2(n\text{-}Bu)]_6Cl_2(thf)_6$ (**8**). S11

NMR-scale reactions of $LuCl_3(thf)_2$ with x *n*-BuLi (x = 1, 3, 6, 12). S11

NMR-scale reactions of *n*-BuLi with 1,3-diphenylpropan-2-one. S11

Ketone/alcohol transformations employing 1,3-diphenylpropan-2-one S11

Ketone/alcohol transformations employing 1,3-diphenylpropan-2-one S12

$CeCl_3$ and 6 *n*-BuLi and ketone (Table 1, entry 2). S12

$Li_3Ce(n\text{-}Bu)_6(thf)_4$ (**3**$^{\mathbf{Ce}}$) and ketone/THF (Table 1, entries 3 and 4). S12

$Li_3Ce(n\text{-}Bu)_6(thf)_4$ (**3**$^{\mathbf{Ce}}$) and ketone/OEt_2 (Table 1, entries 5, 6, and 7). S12

$Li_3Ce(n\text{-}Bu)_6(thf)_4$ ($\mathbf{3^{Ce}}$) and ketone/toluene (Table 1, entry 8)........ S13

$Li_3Ce(n\text{-}Bu)_6(thf)_4$ ($\mathbf{3^{Ce}}$) and ketone/THF/LiCl (Table 1, entry 9). S13

$Li_3Ce(n\text{-}Bu)_6(thf)_4$ ($\mathbf{3^{Ce}}$) and ketone/THF/$CeCl_3$(thf) (Table 1, entry 10). S13

$Li_3Ce(n\text{-}Bu)_6(thf)_4$ ($\mathbf{3^{Ce}}$) and ketone/THF/"cerium turbo chloride" (Table 1, entry 11). S13

$Li_3Ce(n\text{-}Bu)_6(thf)_4$ ($\mathbf{3^{Ce}}$) and ketone/THF, then "cerium turbo chloride" (Table 1, entry 12)........ S14

$Li_3Ce(n\text{-}Bu)_6(thf)_4$ ($\mathbf{3^{Ce}}$) and ketone/THF, then $Sc(OTf)_3$ (Table 1, entry 13). S14

$Li_3Ce(n\text{-}Bu)_6(thf)_4$ ($\mathbf{3^{Ce}}$) and ketone/THF, then $AlCl_3$ (Table 1, entry 14)........ S14

$Li_3Ce(n\text{-}Bu)_6(thf)_4$ ($\mathbf{3^{Ce}}$) and ketone/THF/TMEDA (Table 1, entry 15). S14

$Li_3Lu(n\text{-}Bu)_6(thf)_4$ ($\mathbf{3^{Lu}}$) and ketone/THF (Table 1, entry 16)........ S15

$Li_3Lu(n\text{-}Bu)_6(thf)_4$ ($\mathbf{3^{Lu}}$) and ketone/THF/TMEDA (Table 1, entry 17). S15

$Li_3Y(n\text{-}Bu)_6(thf)_4$ ($\mathbf{3^{Y}}$) and ketone/THF (Table 1, entry 18). S15

$Li_3Y(n\text{-}Bu)_6(thf)_4$ ($\mathbf{3^{Y}}$) and ketone/THF @ ambient temperature (Table 1, entry 19)........ S15

n-BuLi and ketone/THF (Table 1, entry 20). S16

X-Ray Crystallography........ S16

Table S1. X-ray crystallographic parameters for complexes **1**, **2**, and $\mathbf{3^{Lu}}$ S17

Table S2. X-ray crystallographic parameters for complexes $\mathbf{4^{Ce}}$, $\mathbf{4^{Lu}}$, and **5**........ S18

Table S3. X-ray crystallographic parameters for complexes **6**, **7**, and **8**........ S19

Figure S1. Crystal structure of $Li_3Ce_2(ONep)_9(HONep)_2(thf)$ (**6**) S20

Figure S2. Crystal structure of $Li_4[OC(=CHPh)(CH_2Ph)]_4(thf)_4$ (**7**)........ S21

Figure S3. Crystal structure of $Li_8[OCMe_2(n\text{-}Bu)]_6Cl_2(thf)_6$ (**8**)........ S21

NMR Spectra S22

Figure S4. ^1H NMR spectrum of $Li_3CeMe_6(tmeda)_3$ (**1**)........ S22

Figure S5. ^7Li NMR spectrum of $Li_3CeMe_6(tmeda)_3$ (**1**) S22

Figure S6. ^7Li NMR spectrum of $Li_3CeMe_6(tmeda)_3$ (**1**) S23

Figure S7. ^1H NMR spectrum of $[Li(thf)_4][Ce(t\text{-}Bu)_4]$ (**2**)........ S23

Figure S8. ^7Li NMR spectrum of $[Li(thf)_4][Ce(t\text{-}Bu)_4]$ (**2**) S24

Figure S9. ^7Li NMR spectrum of *n*-BuLi S24

Figure S10. ^7Li NMR spectrum of *n*-BuLi + 1 equiv. of THF........ S25

Figure S11. ^7Li NMR spectrum of *n*-BuLi........ S25

Figure S12. ^7Li NMR spectrum of *n*-BuLi + 1 equiv. of TMEDA S26

Figure S13. ^{7}Li NMR spectrum of *n*-BuLi + 1 equiv. of TMEDA S26

Figure S14. ^{1}H NMR spectrum of $Li_3Sc(n\text{-}Bu)_6(thf)_4$ ($\mathbf{3^{Sc}}$) S27

Figure S15. $^{13}C\{^{1}H\}$ NMR spectrum of $Li_3Sc(n\text{-}Bu)_6(thf)_4$ ($\mathbf{3^{Sc}}$) S27

Figure S16. ^{7}Li NMR spectrum of $Li_3Sc(n\text{-}Bu)_6(thf)_4$ ($\mathbf{3^{Sc}}$) S28

Figure S17. ^{45}Sc NMR spectrum of $Li_3Sc(n\text{-}Bu)_6(thf)_4$ ($\mathbf{3^{Sc}}$) S28

Figure S18. VT ^{1}H NMR spectra of $Li_3Sc(n\text{-}Bu)_6(thf)_4$ ($\mathbf{3^{Sc}}$) S29

Figure S19. ^{1}H NMR spectrum of $Li_3Y(n\text{-}Bu)_6(thf)_4$ ($\mathbf{3^{Y}}$) S29

Figure S20. $^{13}C\{^{1}H\}$ NMR spectrum of $Li_3Y(n\text{-}Bu)_6(thf)_4$ ($\mathbf{3^{Y}}$) S30

Figure S21. ^{7}Li NMR spectrum of $Li_3Y(n\text{-}Bu)_6(thf)_4$ ($\mathbf{3^{Y}}$) S30

Figure S22. ^{1}H-^{89}Y HSQC NMR spectrum of $Li_3Y(n\text{-}Bu)_6(thf)_4$ ($\mathbf{3^{Y}}$) S31

Figure S23. VT ^{1}H NMR spectra of $Li_3Y(n\text{-}Bu)_6(thf)_4$ ($\mathbf{3^{Y}}$) S31

Figure S24. ^{1}H NMR spectrum of $Li_3La(n\text{-}Bu)_6(thf)_4$ ($\mathbf{3^{La}}$) S32

Figure S25. ^{7}Li NMR spectrum of $Li_3La(n\text{-}Bu)_6(thf)_4$ ($\mathbf{3^{La}}$) S32

Figure S26. VT ^{1}H NMR spectra of $Li_3La(n\text{-}Bu)_6(thf)_4$ ($\mathbf{3^{La}}$) S33

Figure S27. Wide ^{1}H NMR spectrum of $Li_3Ce(n\text{-}Bu)_6(thf)_4$ ($\mathbf{3^{Ce}}$) S33

Figure S28. Narrow ^{1}H NMR spectrum of $Li_3Ce(n\text{-}Bu)_6(thf)_4$ ($\mathbf{3^{Ce}}$) S34

Figure S29. ^{1}H NMR spectrum of $Li_3Ce(n\text{-}Bu)_6(thf)_4$ ($\mathbf{3^{Ce}}$) S34

Figure S30. ^{7}Li NMR spectrum of $Li_3Ce(n\text{-}Bu)_6(thf)_4$ ($\mathbf{3^{Ce}}$) S35

Figure S31. ^{7}Li NMR spectrum of $Li_3Ce(n\text{-}Bu)_6(thf)_4$ ($\mathbf{3^{Ce}}$) S35

Figure S32. ^{1}H NMR spectrum of $CeCl_3(thf)$ + 1 equiv. of *n*-BuLi S36

Figure S33. ^{7}Li NMR spectrum of $CeCl_3(thf)$ + 1 equiv. of *n*-BuLi S36

Figure S34. ^{1}H NMR spectrum of $CeCl_3(thf)$ + 3 equivs. of *n*-BuLi S37

Figure S35. ^{7}Li NMR spectrum of $CeCl_3(thf)$ + 3 equivs. of *n*-BuLi S37

Figure S36. VT ^{1}H NMR spectra of $Li_3Ce(n\text{-}Bu)_6(thf)_4$ ($\mathbf{3^{Ce}}$) S38

Figure S37. ^{1}H NMR spectrum of $Li_3Lu(n\text{-}Bu)_6(thf)_4$ ($\mathbf{3^{Lu}}$) S38

Figure S38. ^{1}H NMR spectrum of $Li_3Lu(n\text{-}Bu)_6(thf)_4$ ($\mathbf{3^{Lu}}$) S39

Figure S39. $^{13}C\{^{1}H\}$ NMR spectrum of $Li_3Lu(n\text{-}Bu)_6(thf)_4$ ($\mathbf{3^{Lu}}$) S39

Figure S40. ^{7}Li NMR spectrum of $Li_3Lu(n\text{-}Bu)_6(thf)_4$ ($\mathbf{3^{Lu}}$) S40

Figure S41. ^{7}Li NMR spectrum of $Li_3Lu(n\text{-}Bu)_6(thf)_4$ ($\mathbf{3^{Lu}}$) S40

Figure S42. VT ^{1}H NMR spectra of $Li_3Lu(n\text{-}Bu)_6(thf)_4$ ($\mathbf{3^{Lu}}$) S41

Figure S43. ^{1}H NMR spectrum of $Li_2Ce(n\text{-}Bu)_5(tmeda)_2$ (**4**^{Ce}) S41

Figure S44. ^{7}Li NMR spectrum of $Li_2Ce(n\text{-}Bu)_5(tmeda)_2$ (**4**^{Ce}) S42

Figure S45. ^{1}H NMR spectrum of $Li_2Lu(n\text{-}Bu)_5(tmeda)_2$ (**4**^{Lu}) S42

Figure S46. ^{13}C{^{1}H} NMR spectrum of $Li_2Lu(n\text{-}Bu)_5(tmeda)_2$ (**4**^{Lu}) S43

Figure S47. ^{7}Li NMR spectrum of $Li_2Lu(n\text{-}Bu)_5(tmeda)_2$ (**4**^{Lu}) S43

Figure S48. ^{1}H NMR spectrum of $LiLu(n\text{-}Bu)_3Cl(tmeda)_2$ (**5**) S44

Figure S49. ^{13}C{^{1}H} NMR spectrum of $LiLu(n\text{-}Bu)_3Cl(tmeda)_2$ (**5**) S44

Figure S50. ^{7}Li NMR spectrum of $LiLu(n\text{-}Bu)_3Cl(tmeda)_2$ (**5**) S45

Figure S51. ^{1}H NMR spectrum of $Li_3Ce_2(ONep)_9(HONep)_2(thf)$ (**6**) S45

Figure S52. ^{7}Li NMR spectrum of $Li_3Ce_2(ONep)_9(HONep)_2(thf)$ (**6**) S46

Figure S53. ^{1}H NMR spectrum of $Li_4[OC(=CHPh)(CH_2Ph)]_4(thf)_4$ (**7**) S46

Figure S54. ^{13}C{^{1}H} NMR spectrum of $Li_4[OC(=CHPh)(CH_2Ph)]_4(thf)_4$ (**7**) S47

Figure S55. ^{7}Li NMR spectrum of $Li_4[OC(=CHPh)(CH_2Ph)]_4(thf)_4$ (**7**) S47

Figure S56. ^{1}H NMR spectrum of $Li_8[OCMe_2(n\text{-}Bu)]_6Cl_2(thf)_6$ (**8**) S48

Figure S57. ^{7}Li NMR spectrum of $Li_8[OCMe_2(n\text{-}Bu)]_6Cl_2(thf)_6$ (**8**) S48

Figure S58. ^{1}H NMR spectrum of the reaction of 1,3-diphenylpropan-2-one and *n*-BuLi S49

Figure S59. ^{7}Li NMR spectrum of the reaction of 1,3-diphenylpropan-2-one with *n*-BuLi S49

Figure S60. ^{1}H NMR spectrum of 1,3-diphenylpropan-2-one S50

Figure S61. ^{1}H NMR spectrum of 1,3-diphenyl-2-butylpropan-2-ol S50

Figure S62. ^{1}H NMR spectrum of a mixture of 1,3-diphenylpropan-2-one and 1,3-diphenyl-2-butylpropan-2-ol from the reaction of $Li_3Ce(n\text{-}Bu)_6(thf)_4$ (3^{Ce}) with six equiv. of 1,3-diphenylpropan-2-one S51

References S51

Experimental Section

General Considerations. ***Caution!*** The organocerium compounds are highly pyrophoric and react violently when exposed to air and/or moisture. All manipulations were performed under an inert atmosphere (Ar) using either glovebox (MBraun UNIlab^{pro}; <0.1 ppm O_2, <0.1 ppm H_2O) or standard Schlenk techniques with oven-dried glassware. The solvents were purified with Grubbs columns (MBraun SPS, solvent purification system) and stored in a glovebox. Anhydrous cerium(III) chloride (99.9%), anhydrous lanthanum(III) chloride (99.9%), anhydrous yttrium(III) chloride (99.9%), anhydrous scandium(III) chloride (99.9%), and anhydrous lutetium(III) chloride (99.9%), were purchased from *abcr* and activated by Soxhlet extraction with THF giving $CeCl_3$(thf), $LaCl_3$(thf), $YCl_3(thf)_2$, $ScCl_3(thf)_3$, and $LuCl_3(thf)_2$. A 0.26 M $CeCl_3 \cdot 2$ LiCl THF solution ("Ce turbo chloride") was prepared according to literature.[1] *n*-Butyllithium (*n*-BuLi) (2.5 M solution in hexanes) and 1,3-diphenyl-2-propanon (99%) were purchased from *Sigma Aldrich* and used as received. TMEDA (98%), scandium(III)-trifluoromethanesulfonate $Sc(OTf)_3$ (97%), anhydrous aluminum chloride (99%), and anhydrous lithium chloride (98%) were purchased from *abcr* and used as received. Benzene-d_6, toluene-d_8, and THF-d_8 were purchased from *Sigma Aldrich*, degassed, dried over NaK alloy for 24 h, filtered and stored inside a glovebox. NMR spectra of moisture-sensitive compounds were recorded by using J. Young valve NMR tubes on either a Bruker AVII+400 (^{1}H: 400.13 MHz), a Bruker AVIIIHD (^{1}H: 300.13 MHz), or a Bruker AVII+500 (^{1}H: 500.13 MHz). ^{1}H NMR shifts are referenced to a solvent resonance and reported in parts per million (ppm) relative to tetramethylsilane.[2] Analysis of NMR spectra was performed with TopSpin 3.6.1 [Academic License].[3] Multiplicities of signals are given as s (singlet), bs (broad singlet), d (doublet) and dd (doublet of doublets). Coupling constants (J) are given in Hz. Signals were assigned via 2D NMR experiments. Infrared spectra were recorded on a *ThermoFisher* Scientific NICOLET 6700 FTIR ($\tilde{\nu}$= 4000 – 400 cm^{-1}) spectrometer using a DRIFTS chamber with dry KBr/sample mixtures and KBr windows. Elemental analysis (C, H, N) was performed on an *Elementar vario MICRO cube*. Crystals for X-ray crystallography were handpicked in a glovebox, coated with Parabar 10312, pump-oil, or perfluorinated oil, and stored on microscope slides. X-ray data were collected on a Bruker APEX II DUO diffractometer equipped with an IμS microfocus sealed tube and QUAZAR optics for MoK_α (λ = 0.71073 Å) and CuK_α (λ = 1.54184 Å) radiation. The data collection strategy was determined using COSMO[4] employing ω-scans. Raw data were processed using APEX[5] and SAINT,[6] corrections for absorption effects were applied using SADABS.[7] The structures were solved by direct methods and refined against all data by full-matrix least-squares methods on F^2 using SHELXTL[8] and ShelXle.[9] Disorder models were calculated using DSR, a program for refining structures in ShelXl.[10] All graphics were produced employing Mercury 4.2.0[11] and POV-Ray.[12]

[$Li_3CeMe_6(tmeda)_3$] (1). A slightly modified procedure from that published by Schumann and coworkers[13] was used to obtain cerous compound **1**. $CeCl_3$ (303.0 mg, 1.23 mmol) was suspended in

THF (10 mL), cooled to –10 °C, and TMEDA (714.3 mg, 6.25 mmol, 5 equiv.) added dropwise. After stirring for 30 min, MeLi (243.9 mg, 11.06 mmol, 9 equiv.) was added, which turned the suspension instantly into a yellowish/greenish solution. After 18 h, the solvent was removed in vacuo and the remaining solid extracted with cold Et_2O. The ethereal solution was concentrated under reduced pressure and stored at –40 °C, yielding yellow crystals of **1** (83%) suitable for XRD analysis. ^1H NMR (500.13 MHz, 223 K, THF-d_8): δ = 6.47 (bs, 36 H, CH_3 tmeda), 5.82 (bs, 12 H, CH_2 tmeda), –4.08 (bs, 18 H, Ce–CH_3) ppm. ^7Li NMR, (194.37 MHz, 223 K, THF-d_8): δ = 43.3 and 42.8 (Li_3CeMe$_6$(tmeda)$_3$, 97%), 2.4 and 1.8 (Me*Li*(tmeda), 3%) ppm. IR (KBr, cm^{-1}) ν = 2981 (m), 2952 (m), 2833 (m), 2789 (m), 2749 (w), 1464 (m), 1356 (w), 1291(m), 1255 (w), 1157 (m), 1038 (m), 1021 (w), 948 (m), 790 (w) 475 (s), 445 (s). Anal. (%) Calcd. for $C_{24}H_{66}CeLi_3N_6$ (599.77 g mol^{-1}): C 48.06, H 11.09, N 14.01; found: C 47.48, H 10.70, N 13.87. The analysis is slightly off because of a small LiCl impurity.

[Li(thf)$_4$][Ce(*t*-Bu)$_4$] (2). $CeCl_3$(thf) (63.7 mg, 0.20 mmol) was suspended in THF (0.5 mL) and cooled to –40 °C. Then, *t*-BuLi (51.2 mg, 0.80 mmol) dissolved in *n*-hexane was added dropwise. After stirring the suspension for 30 min, it was filtered and the solvent removed in vacuo to give a red powder of [Ce(*t*-Bu)$_4$][Li(thf)$_4$]•*x*LiCl in approximate 65% yield. Crystals of **2** suitable for XRD analysis were obtained from a highly concentrated Et_2O solution. Note: The oily consistency of the crystallization process is not a suitable method for purification. Amount of LiCl was determined via elemental analysis. ^1H NMR, (300.13 MHz, 299 K, THF-d_8): δ = 3.62(m, 16 H, thf) 2.39 (bs, 36 H, *t*-Bu), 1.78 ppm (m, 16 H, thf). ^7Li NMR, (116.64 MHz, 299 K, THF-d_8): δ = 0.4 (bs, *Li*(thf)$_4$) ppm. Anal. (%) calcd. for $C_{32}H_{68}CeLiO_4$ (663.95 gmol-1): C 57.89 49.92, H 10.32 8.90; found: C 49.65, H 7.66. The deviation between theoretical and experimental microanalytical data derives from LiCl incorporation, e.g., the presence of 2.5 LiCl per molecule would correspond to C 49.92, H 8.90.

General Procedure for the Synthesis of Li$_3$Ln(*n*-Bu)$_6$(thf)$_4$ (3Ln, Ln = Sc, Y, La, Ce, Lu). $LnCl_3(thf)_x$ (0.2 mmol) was suspended in *n*-hexane (1 mL) and THF (0.4 mL), and *n*-BuLi (2.5 M in hexanes, 480.0 µL, 1.2 mmol was added dropwise at –40 °C. The mixture was stirred for 30 min at –40 °C, then filtered, and the solvent evaporated in vacuo at –40 °C. The residue was extracted with cold *n*-hexane (~ 8 mL) and the extract filtered again. The solution was slowly concentrated under reduced pressure at –40 °C and stored at –40 °C for crystallization giving $\mathbf{3^{Ln}}$. Notes: Complexes $\mathbf{3^{Ln}}$ of the larger Ln (Ce and La) are better soluble in *n*-hexane than the complexes of the smaller Ln (Y, Lu, and Sc), but the latter are more stable. For example, $\mathbf{3^{Lu}}$ is stable in a *n*-hexane solution for months, whereas $\mathbf{3^{Ce}}$ had fully decomposed after about three days (at –40 °C). Solvent-free crystals however were stable for all complexes (at –40 °C) except for $\mathbf{3^{La}}$ which could only be obtained as an oil and had decomposed after a week. The ^1H and ^7Li NMR spectra indicated partial cleavage of *n*-BuLi, dependent on the Ln(III) size and the solvent.

Li$_3$Sc(*n*-Bu)$_6$(thf)$_4$ ($\mathbf{3^{Sc}}$). Colorless solid, yield: 44.7 mg (0.06 mmol, 32%). ^1H NMR (500.13 MHz, 233 K, toluene-d_8): δ = 3.65 (m, 16 H, thf), 2.09 (m, 12 H, CH_2-2-butyl), 1.84 (m, 12 H, CH_2-3-butyl), 1.36

(m, 34 H, CH_3-4-butyl and thf), 0.33 ppm (m, 12 H, CH_2-1-butyl). $^{13}C\{^1H\}$ NMR (125.76 MHz, 233 K, toluene-d_8): δ = 68.1 (s, thf), 34.0 (s, CH_2-2-butyl), 32.8 (s, CH_2-3-butyl), 29.9 (s, CH_2-1-butyl), 25.3 (s, thf), 14.6 ppm (s, CH_3-4-butyl). ^7Li NMR (194.37 MHz, 233 K, toluene-d_8): δ = 2.3 (free *n*-Bu*Li*(thf), ~8%), 1.2 ppm ($Li_3Sc(n\text{-}Bu)_6(thf)_4$, ~92%). ^{45}Sc NMR (121.49 MHz, 233 K, toluene-d_8): δ = 502 ppm (s, lwhh = 11787 Hz).

$Li_3Y(n\text{-}Bu)_6(thf)_4$ (3^Y). Colorless solid, yield: 93.7 mg (0.13 mmol, 64%). ^1H NMR (500.13 MHz, 233 K, toluene-d_8): δ = 3.59 (m, 16 H, thf), 2.01 (m, 12 H, CH_2-2-butyl), 1.80 (m, 12 H, CH_2-3-butyl), 1.31 (m, 34 H, CH_3-4-butyl and thf), 0.05 ppm (m, 12 H, CH_2-1-butyl). $^{13}C\{^1H\}$ NMR (125.76 MHz, 233 K, toluene-d_8): δ = 68.1 (s, thf), 33.3 (s, CH_2-2-butyl), 33.1 (s, CH_2-3-butyl), 29.2 (s, CH_2-1-butyl), 25.3 (s, thf), 14.5 ppm (s, CH_3-4-butyl). ^7Li NMR (194.37 MHz, 233 K, toluene-d_8): δ = 2.3 (free *n*-Bu*Li*(thf), ~1%), 1.6 ppm ($Li_3Y(n\text{-}Bu)_6(thf)_4$, ~99%). ^{89}Y (from ^1H-^{89}Y HSQC) NMR (24.52 MHz, 233 K, toluene-d_8): δ = 771 ppm (s, lwhh = 143 Hz). Anal. (%) Calcd. for $C_{40}H_{86}Li_3YO_4$ (740.85 gmol^{-1}): C 64.85, H 11.70; found: C 64.74, H 12.51.

$Li_3La(n\text{-}Bu)_6(thf)_4$ (3^{La}). Colorless solid/oil, yield: ~50% yield (no complete removal of THF at –40 °C). Because most of the *n*-BuLi is cleaved off in solution, not all signals could be assigned with complete accuracy in the ^1H NMR spectrum. ^1H NMR (500.13 MHz, 233 K, toluene-d_8): δ = 3.67 (m, thf), 1.86 (m, CH_2-2-butyl, CH_2-3-butyl, and *n*-BuLi), 1.29 (m, CH_2-4-butyl, and thf), 0.91 (t, *n*-BuLi), 0.00 (bs, CH_2-1-butyl), –0.53 ppm (m, *n*-BuLi). ^7Li NMR (194.37 MHz, 233 K, toluene-d_8): δ = 2.3, 2.1 and 1.70 (*n*-Bu*Li*(thf)).

$Li_3Ce(n\text{-}Bu)_6(thf)_4$ (3^{Ce}). Yellow solid, yield: 146.8 mg (0.19 mmol, 93%). Due to the paramagnetism of Ce(III) the ^1H NMR spectrum was not conclusive (see Figure S25–S27) . ^7Li NMR (194.37 MHz, 233 K, toluene-d_8): δ = 30.2 (*Li*[Ce], ~2%), 2.4, 2.0 ppm (free *n*-Bu*Li*(thf), ~98%). Elemental analysis calcd. (%) for $C_{40}H_{86}Li_3CeO_4$ (792.06 gmol^{-1}): C 61.12, H 10.26; found: C 61.34, H 10.50.

$Li_3Lu(n\text{-}Bu)_6(thf)_4$ (3^{Lu}). Colorless solid, yield: 121.9 mg (0.14 mmol, 74%). ^1H NMR (500.13 MHz, 233 K, toluene-d_8): δ = 3.59 (m, 16 H, thf), 2.01 (m, 12 H, CH_2-2-butyl), 1.79 (m, 12 H, CH_2-3-butyl), 1.30 (m, 34 H, CH_3-4-butyl, and thf), 0.14 ppm (m, 12 H, CH_2-1-butyl). $^{13}C\{^1H\}$ NMR (125.76 MHz, 233 K, toluene-d_8): δ = 68.4 (s, thf), 33.9 (s, CH_2-2-butyl), 33.6 (s, CH_2-3-butyl), 33.0 (s, CH_2-1-butyl), 25.5 (s, thf), 14.8 ppm (s, CH_3-4-butyl). ^7Li NMR (194.37 MHz, 233 K, toluene-d_8): δ = 2.3 (free *n*-Bu*Li*(thf), ~4%), 1.9 ppm ($Li_3Lu(n\text{-}Bu)_6(thf)_4$, ~96%). Anal. (%) Calcd. for $C_{40}H_{86}Li_3LuO_4$ (826.87 gmol^{-1}): C 58.10, H 10.48; found: C 57.93, H 10.50.

Equimolar reaction of $CeCl_3$(thf) with *n*-BuLi. $CeCl_3$(thf) (100.0 mg, 0.31 mmol) was suspended in THF and cooled to –40 °C. Then, *n*-BuLi (2.5 M in hexanes, 125.6 µL,0.31 mmol, one equiv.) was added and the mixture stirred for 30 min. After allowing time for settling of the insoluble parts, the supernatant was carefully removed. The residue was evaporated in vacuo to leave ~44 mg (44%) of unconsumed $CeCl_3$(thf).

Synthesis of 3^{Lu} for XRD analysis. $LuCl_3(thf)_2$ (72.5 mg, 0.18 mmol) was suspended in *n*-hexane (2 mL) and *n*-BuLi (2.5 M in hexanes, 240.0 µL, 0.6 mmol, 3.33 equiv.) was added dropwise at –45 °C. Stirring the mixture for 24 h at –45 °C caused the formation of an oily white precipitate. After filtration the solution was concentrated (under reduced pressure) and stored at – 40 °C to give colorless crystals suitable for XRD analysis in low yields (~5-10%).

Synthesis of 3^{Lu} from "lutetium turbo chloride". $LuCl_3(thf)_2$ (83.4 mg, 0.2 mmol) and LiCl (17.0 mg, 0.4 mmol were stirred together in THF (5 mL). Complete dissolution of the mixed metal chloride was observed after 30 min. The solution was cooled to –40 °C and *n*-BuLi (2.5 M in hexanes, 480.0 µL,1.2 mmol) was added dropwise. The mixture was stirred for 30 min at –40 °C and then filtered. The solvent of the filtrate was evaporated in vacuo at –40 °C, extracted with cold *n*-hexane (~ 8 mL), and the extract filtered again. The obtained solution was slowly concentrated under reduced pressure at –40 °C and stored at –40 °C for crystallization. The obtained colorless crystals were identified as **3^{Lu}** (~70%) via XRD unit-cell check and ^{1}H NMR spectroscopy.

Synthesis of 3^{Lu} from 3^{Ce}. To a suspension of $LuCl_3(thf)_2$ (56.4 mg, 0.14 mmol) in THF (0.4 mL) and *n*-hexane (1 mL), compound **3^{Ce}** (106.5 mg, 0.14 mmol) dissolved in *n*-hexane (3 mL) was added at –40 °C. After stirring for 18 h at –40 °C the brown mixture was filtered and concentrated to give **3^{Lu}** (26.3 mg, 0.03 mmol, 23%) as colorless crystals (characterized by XRD unit-cell check).

General Procedure for the Synthesis of $Li_2Ln(n\text{-}Bu)_5(tmeda)_2$ (4^{Ln}, Ln = Ce, Lu). $LnCl_3(thf)_x$ (0.2 mmol) was suspended in *n*-hexane (1 mL) and THF (0.4 mL) and *n*-BuLi (2.5 M in hexanes, 480.0 µL, 1.2 mmol, six equiv.) were added dropwise at –40 °C. The mixture was stirred for 30 min at –40 °C. After filtration the solvent was evaporated in vacuo at –40 °C, the obtained residue extracted with cold *n*-hexane (~ 8 mL) and the extract filtered again. Cold TMEDA (90.5 µL, 0.6 mmol, three equiv.) was added to the solution before it was slowly concentrated under reduced pressure at –40 °C until the onset of crystallization. Storage at – 40 °C yielded single-crystalline **4^{Ln}**.

$Li_2Ce(n\text{-}Bu)_5(tmeda)_2$ (4^{Ce}). 73.2 mg (0.11 mmol, 55%). Due to the paramagnetism of Ce(III) the ^{1}H NMR spectrum was not conclusive (see Figure S41) . ^{7}Li NMR (194.37 MHz, 233 K, toluene-d_8): δ = 79.7 (*Li*[Ce], ~54%), 2.6 ppm (free *n*-Bu*Li*(tmeda), ~46%). Anal. (%) Calcd. for $C_{32}H_{77}Li_2CeN_4$ (671.97 g mol^{-1}): C 57.54, H 11.02, N 8.39; found: C 58.78, H 12.59, N 8.63. The deviation between theoretical and experimental microanalytical data derives from decomposition of **4^{Ce}** at ambient temperature (change from crystalline to oily consistency).

$Li_2Lu(n\text{-}Bu)_5(tmeda)_2$ (4^{Lu}). Colorless solid, yield: 87.8 mg (0.12 mmol, 62%). ^{1}H NMR (500.13 MHz, 233 K, toluene-d_8): δ = 2.06 (bs, 24 H, tmeda-CH_3), 1.98 (m, 10 H, CH_2-2-butyl), 1.86 (m, 10 H, CH_2-3-butyl), 1.64 (bs, 8 H, tmeda-CH_2), 1.37 (t, 15 H, CH_3-4-butyl), 0.05 ppm (m, 10 H, CH_2-1-butyl). ^{13}C{^{1}H} NMR (125.76 MHz, 233 K, toluene-d_8): δ = 56.3 (s, tmeda-CH$_2$), 45.8 (s, tmeda-CH$_3$), 41.8 (s, CH$_2$-1-butyl), 32.8 (s, CH$_2$-3-butyl), 32.0 (s, CH$_2$-2-butyl), 14.8 ppm (s, CH$_3$-4-butyl). ^{7}Li NMR (194.37

MHz, 233 K, toluene-d_8): δ = 2.5 (free *n*-Bu*Li*(tmeda), ~20%), 1.8 ppm (*Li*$_2$Lu(*n*-Bu)$_5$(tmeda)$_2$, ~80%). Anal. (%) Calcd. for $C_{32}H_{77}Li_2LuN_4$ (706.82 gmol^{-1}): C 54.69, H 10.47, N 7.97; found: C 53.73, H 11.32, N 8.58. The deviation between theoretical and experimental microanalytical data derives from decomposition of **4^{Lu}** at ambient temperature (change form crystalline to oily consistency).

LiLu(*n*-Bu)$_3$Cl(tmeda)$_2$ (5). Li$_2$Lu(*n*-Bu)$_5$(tmeda)$_2$ (**4^{Lu}**) (87.8 mg, 0.12 mmol) was dissolved in *n*-hexane (4 mL) at –40 °C and ClSiMe$_3$ (76.3 µL, 0.60 mmol, five equiv.) was added. After stirring for 4 h the mixture was filtered and the volatiles were removed in vacuo at –40 °C. The crude product was redissolved in *n*-hexane, and after filtration and concentration under reduced pressure at –40 °C, compound **5** was obtained as colorless crystals, suitable for XRD analysis, albeit in a low yield. ^{1}H NMR (500.13 MHz, 233 K, THF-d_8): δ = 2.32 (s, 8 H, tmeda-C*H*$_2$), 2.17 (s, 24 H, tmeda-C*H*$_3$), 1.64 (m, 6 H, C*H*$_2$-2-butyl), 1.25 (m, 4 H, C*H*$_2$-3-butyl), 1.19 (m, 2 H, C*H*$_2$-3-Li-butyl), 0.84 (m, 9 H, C*H*$_2$-4-butyl), –0.39 (m, 4 H, C*H*$_2$-1-butyl), –0.54 ppm (m, 2 H, C*H*$_2$-1-Li-butyl). ^{13}C{^{1}H} NMR (125.76 MHz, 233 K, THF-d_8): δ = 58.0 (s, tmeda-*C*H$_2$), 52.8 (s, *C*H$_2$-1-Li-butyl), 49.8 (s, *C*H$_2$-1-butyl), 45.5 (s, tmeda-*C*H$_3$), 34.2 (s, *C*H$_2$-2-Li-butyl) 33.1 (s, *C*H$_2$-2-butyl), 32.8 (s, *C*H$_2$-3-Li-butyl), 31.6 (s, *C*H$_2$-3-butyl), 14.4 (s, *C*H$_3$-4-Li-butyl) 14.0 ppm (s, *C*H$_3$-4-butyl). ^{7}Li NMR (194.37 MHz, 233 K, THF-d_8): δ = 0.28 ppm. The small amount of product was not sufficient for microanalysis (NMR study was prioritized).

Synthesis of Li$_3$Ce$_2$(ONep)$_9$(HONep)$_2$(thf) (6). Li$_3$Ce(*n*-Bu)$_6$(thf)$_4$ (**3^{Ce}**) (133.8 mg, 0.20 mmol) was dissolved in *n*-hexane (4 mL) at –40 °C and solution of neopentanol (105.8 mg, 1.20 mmol, six equiv.) in *n*-hexane was added. After stirring for 30 min the mixture was concentrated under reduced pressure at –40 °C and filtered. The obtained solution was stored at –40 °C to afford **6** as colorless crystals, suitable for XRD analysis, albeit in a low yield. ^{1}H NMR (300.13 MHz, 299 K, benzene-d_6): δ = 8.23 (bs, 2 H, O*H*), 3.76 (bs, 22 H, C*H*$_2$), 3.62 (bs, 4 H, thf), 1.04 (bs, 4 H, thf), 0.55 ppm (bs, 99 H, C*H*$_3$). ^{7}Li NMR (116.64 MHz, 299 K, benzene-d_6): δ = 91.76, 65.11, 57.55, 30.67, 26.28, 23.75, 22.83, 13.09, 10.80, 3.76, 2.59, 1.85, 0.80 ppm. Anal. (%) Calcd. for $C_{59}H_{131}Ce_2Li_3O_{12}$ (1333.69 gmol^{-1}): C 53.13, H 9.90; found: C 51.76, H 11.90. The elemental analysis is slightly off, because of some remaining solvent.

Li$_4$[OC(=CHPh)(CH$_2$Ph)]$_4$(thf)$_4$ (7). CeCl$_3$(thf) (63.7 mg, 0.2 mmol) was suspended in THF (0.4 mL), *n*-hexane (1 mL) and cooled to –40 °C. Then *n*-BuLi (2.5 M in hexanes, 480.0 µL, 1.2 mmol, six equiv.) was added dropwise. After 30 min of stirring at –40 °C cold 1,3-diphenylpropan-2-one (252.3 mg, 1.2 mmol, six equiv.) was added to the yellow solution, which instantly decolorized to slightly yellow. The mixture was stirred for another 30 min at –40 °C. Then it was filtered, concentrated under reduced pressure, and stored at –40 °C for three weeks, which produced heavily intertwined colorless crystals. The yellow supernatant was removed and the crystals were recrystallized several times from a *n*-hexane/THF mixture affording colorless crystals of **7**. Yield: 69.3 mg, 0.05 mmol, 18% (after crystals had been removed for XRD analysis). ^{1}H NMR (300.13 MHz, 299 K, THF-d_8; numbering scheme, see Figures S51 and S52): δ = 7.58 (m, 2 H, CH-aromatic 5&9), 7.31 (m, 2 H, CH-aromatic 11&15), 7.19 (m, 2 H, CH-aromatic 12&14), 7.10 (m, 1 H, CH-aromatic 13), 7.00 (m, 2 H, CH-aromatic 6&8), 6.66

(m, 1 H, CH-aromatic 7), 4.75 (s, 1 H, CH 1), 3.61 (m, 4 H, THF), 3.38 (s, 2 H, CH_2 3), 1.76 ppm (m, 4 H, THF). $^{13}C\{^1H\}$ NMR (75.47 MHz, 299 K, THF-d_8): δ = 167.6 (s, C 2), 143.1 (s, C 10), 142.1 (s, C 4), 129.0 (s, C 11&15), 127.5 (s, C 12&14), 127.1 (s, C 6&8), 124.9 (s, C 13), 124.8 (s, C 5&9), 119.9 (s, C 7), 96.1 (s, C 1), 67.2 (s, THF), 49.1 (s, C 3), 25.4 ppm (s, THF). ^7Li NMR (116.64 MHz, 299 K, THF-d_8): δ = 0.14 ppm.

Synthesis of $Li_8[OCMe_2(n\text{-}Bu)]_6Cl_2(thf)_6$ (8). $CeCl_3$(thf) (63.7 mg, 0.2 mmol) was suspended in THF (2 mL) and cooled to –40 °C. Then *n*-BuLi (2.5 M in hexanes, 240.0 µL, 0.6 mmol, three equiv.) was added dropwise. After 30 min of stirring at –40 °C, cold acetone (44.4 µL, 0.6 mmol, three equiv.) was added to the yellow solution which decolorized immediately. The mixture was stirred for another 30 min at –40 °C before it was filtered and stored at –40 °C for two weeks to yield colorless crystals of **8** in a low yield.

NMR-scale reactions of $LuCl_3(thf)_2$ with x *n*-BuLi (x = 1, 3, 6, 12). In a J.-Young valved NMR tube $LuCl_3(thf)_2$ was suspended in THF-d_8 and cooled down to –40 °C. Then *x* (1, 3, 6, 12) equivalents of *n*-BuLi were added. After 30 min at –40 °C, during which the NMR tube was regularly shaken ^1H and ^7Li NMR spectra were measured at –80 °C.

NMR-scale reactions of *n*-BuLi with 1,3-diphenylpropan-2-one. In a J.-Young valved NMR tube 1,3-diphenylpropan-2-one (21.0 mg, 0.1 mmol) was dissolved in THF-d_8 and *n*-BuLi (2.5 M in hexanes, 40.0 µL, 0.1 mmol, one equiv.) was added, upon which the solution turned deep red immediately. After 30 min during which time the NMR tube was regularly shaken ^1H and ^7Li NMR spectra were measured (see Figures S56 and S57). The NMR spectra appear intricate, but clearly show that both the $Li_4[OC(=CHPh)(CH_2Ph)]_4(thf)_4$ and the lithium salt of 1,3-diphenyl-2-butylpropan-2-ol are produced alongside of further unknown side products. The NMR spectra did not change after 18 h.

Ketone/alcohol transformations employing 1,3-diphenylpropan-2-one. A detailed reaction is described as a typical example (Table 1, entry 1). For the other reactions, see the supporting information. The ketone reduction with an equimolar amount of $CeCl_3$ and *n*-BuLi was adapted from the original work by the Imamoto group in 1984.[7] A suspension of $CeCl_3$(thf) (414.2 mg, 1.3 mmol) in THF (5 mL) was cooled to –35 °C. Then *n*-BuLi (2.5 M in hexanes, 520.0 µL, 1.3 mmol, one equiv.) was added dropwise. After 30 min of stirring at –35 °C a pre-cooled solution of 1,3-diphenylpropan-2-one (210.3 mg, 1.0 mmol, 0.77 equiv.) dissolved in THF (5 mL) was added and the mixture stirred for another 30 min at –35 °C. The mixture was then quenched with a saturated aqueous solution of sodium carbonate, and the aqueous layer was extracted three times with diethyl ether. The combined organic layers were dried over anhydrous sodium sulfate and the volatiles were removed in vacuo giving 1,3-diphenyl-2-butylpropan-2-ol as a colorless oil. Yield: 266.8 mg (0.99 mmol, 99%). ^1H NMR (400.11 MHz, 299 K, benzene-d_6): δ = 7.18-7.03 (m, 12 H, phenyl), 2.65 (s, 4 H, 1,3-CH_2-propan), 1.33-1.06 (m, 6 H, CH_2-1-butyl, CH_2-2-butyl, and CH_2-3-butyl), 0.81 ppm (t, 3 H, CH_3-4-butyl).

Ketone/alcohol transformations employing 1,3-diphenylpropan-2-one. For incomplete reactions, the ketone/alcohol ratio was determined via the CH_2 groups of the propane part of the molecules in the ^{1}H NMR spectra: for 1,3-diphenylpropan-2-one, one singlet at 3.33 ppm; for 1,3-diphenyl-2-butylpropan-2-ol, one singlet at 2.65 ppm. Due to equal number of protons, comparison of the integrals of these two signals will indicate the molar ratio of the ketone and alcohol. Weighting of the ratio with the molecular weight of the ketone and alcohol lead to the yield according to the formula shown below.

$$\text{alcohol [mmol]} = \frac{\text{yield of alcohol + ketone [mg]}}{\text{alcohol}[M] + \left(\frac{\text{ketone[mol \%]}}{\text{alcohol [mol \%]}}\right) \times \text{ketone}[M]}$$

$CeCl_3$ and 6 *n*-BuLi and ketone (Table 1, entry 2). A suspension of $CeCl_3$(thf) (63.7mg, 0.2 mmol) in THF (2 mL) was cooled to –40 °C before *n*-BuLi (2.5 M in hexanes, 480.0 µL, 1.2 mmol, six equiv.) was added dropwise. After 30 min of stirring at –40 °C a pre-cooled solution of 1,3-diphenylpropan-2-one (252.3 mg, 1.2 mmol, six equiv.) dissolved in THF (2 mL) was added and the mixture stirred for another 30 min at –40 °C. The mixture was then quenched with a saturated aqueous solution of sodium carbonate, and the aqueous layer was extracted three times with diethyl ether. The combined organic layers were dried over anhydrous sodium sulfate and the volatiles were removed in vacuo giving 1,3-diphenyl-2-butylpropan-2-ol as a colorless oil. Yield: 246.9 mg (0.92 mmol, 77%); amount of recovered ketone: 50.5 mg (0.24 mmol, 20%).

Li_3Ce(*n*-Bu)$_6$(thf)$_4$ ($\mathbf{3^{Ce}}$) and ketone/THF (Table 1, entries 3 and 4). A chilled solution of $\mathbf{3^{Ce}}$ (158.4 mg, 0.2 mmol) in *n*-hexane (2 mL, –40 °C) was added dropwise to a pre-cooled solution of 1,3-diphenylpropan-2-one (252.3 mg/1.2 mmol/six equiv. or 42.1 mg/0.2 mmol/one equiv.) dissolved in THF (2 mL) and stirred for 30 min at –40 °C. The mixture was then quenched with a saturated aqueous solution of sodium carbonate, and the aqueous layer was extracted three times with diethyl ether. The combined organic layers were dried over anhydrous sodium sulfate and the volatiles were removed in vacuo giving 1,3-diphenyl-2-butylpropan-2-ol as a colorless oil. Yield for six equivs. of ketone: 226.1 mg (0.84 mmol, 70%); amount of recovered ketone: 33.7 mg (0.16 mmol, 13%). Yield for one equiv. of ketone: 47.2 mg (0.18 mmol, 88%); amount of recovered ketone 1.2 mg (0.01 mmol, 3%).

Li_3Ce(*n*-Bu)$_6$(thf)$_4$ ($\mathbf{3^{Ce}}$) and ketone/OEt_2 (Table 1, entries 5, 6, and 7). A chilled solution of $\mathbf{3^{Ce}}$ (158.4 mg, 0.2 mmol) in *n*-hexane (2 mL, –40 °C) was added dropwise to a pre-cooled solution of 1,3-diphenylpropan-2-one (252.3 mg/1.2 mmol/six equiv. or 126.2 mg/0.6 mmol/three equiv. or 42.1 mg/0.2 mmol/one equiv.) dissolved in Et_2O (2 mL) and stirred for 30 min at –40 °C. The mixture was then quenched with a saturated aqueous solution of sodium carbonate, and the aqueous layer was extracted three times with diethyl ether. The combined organic layers were dried over anhydrous sodium sulfate

and the volatiles were removed in vacuo giving 1,3-diphenyl-2-butylpropan-2-ol as a colorless oil. Yield for six equiv. of ketone: could not be determined gravimetrically because the volatiles were not removed completely, so only the NMR ratio of the alcohol and ketone are stated here: alcohol 54%, ketone 46%. Yield for three equivs. of ketone: 125.8 mg (0.47 mmol, 78%); amount of recovered ketone: 19.3 mg (0.09 mmol, 15%). Yield for one equiv. of ketone: 47.9 mg (0.18 mmol, 89%); amount of recovered ketone: 4.1 mg (0.02 mmol, 10%).

Li_3Ce(*n*-Bu)$_6$(thf)$_4$ (3^{Ce}) and ketone/toluene (Table 1, entry 8). A chilled solution of $\mathbf{3^{Ce}}$ (158.4 mg, 0.2 mmol) in *n*-hexane (2 mL, –40 °C) was added dropwise to a pre-cooled solution of 1,3-diphenylpropan-2-one (252.3 mg, 1.2 mmol, six equiv.) dissolved in toluene (2 mL) and stirred for 30 min at –40 °C. The mixture was then quenched with a saturated aqueous solution of sodium carbonate, and the aqueous layer was extracted three times with diethyl ether. The combined organic layers were dried over anhydrous sodium sulfate and the volatiles were removed in vacuo giving 1,3-diphenyl-2-butylpropan-2-ol as a colorless oil. Yield: 243.7 mg (0.91 mmol, 76%); amount of recovered ketone: 28.5 mg (0.14 mmol, 11%).

Li_3Ce(*n*-Bu)$_6$(thf)$_4$ (3^{Ce}) and ketone/THF/LiCl (Table 1, entry 9). A chilled solution of $\mathbf{3^{Ce}}$ (158.4 mg, 0.2 mmol) in *n*-hexane (2 mL, –40 °C) was added dropwise to a pre-cooled mixture of 1,3-diphenylpropan-2-one (252.3 mg, 1.2 mmol, six equiv.) and LiCl (25.4 mg, 0.6 mmol, three equiv.) dissolved in THF (2 mL), and stirred for 30 min at –40 °C. The mixture was then quenched with a saturated aqueous solution of sodium carbonate, and the aqueous layer was extracted three times with diethyl ether. The combined organic layers were dried over anhydrous sodium sulfate and the volatiles were removed in vacuo giving 1,3-diphenyl-2-butylpropan-2-ol as a colorless oil. Yield: 238.9 mg (0.89 mmol, 74%); amount of recovered ketone: 40.3 mg (0.19 mmol, 16%).

Li_3Ce(*n*-Bu)$_6$(thf)$_4$ (3^{Ce}) and ketone/THF/$CeCl_3$(thf) (Table 1, entry 10). A chilled solution of $\mathbf{3^{Ce}}$ (158.4 mg, 0.2 mmol) in *n*-hexane (2 mL, –40 °C) was added dropwise to a pre-cooled mixture of 1,3-diphenylpropan-2-one (252.3 mg, 1.2 mmol, six equiv.) and $CeCl_3$(thf) (318.6 mg, 1.0 mmol, five equiv.) suspended in THF (5 mL), and stirred for 30 min at –40 °C. The mixture was then quenched with a saturated aqueous solution of sodium carbonate, and the aqueous layer was extracted three times with diethyl ether. The combined organic layers were dried over anhydrous sodium sulfate and the volatiles were removed in vacuo giving 1,3-diphenyl-2-butylpropan-2-ol as a colorless oil. Yield: 285.3 mg, (1.06 mmol, 89%), amount of recovered ketone: 20.8 mg (0.10 mmol, 8%).

Li_3Ce(*n*-Bu)$_6$(thf)$_4$ (3^{Ce}) and ketone/THF/"cerium turbo chloride" (Table 1, entry 11). A chilled solution of $\mathbf{3^{Ce}}$ (158.4 mg, 0.2 mmol) in *n*-hexane (2 mL, –40 °C) was added dropwise to a pre-cooled mixture of 1,3-diphenylpropan-2-one (252.3 mg, 1.2 mmol, six equiv.) and "cerium turbo chloride" (1.0 mmol, five equiv.) dissolved in THF (5 mL) and stirred for 30 min at –40 °C. The mixture was then quenched with a saturated aqueous solution of sodium carbonate, and the aqueous layer was extracted three times with diethyl ether. The combined organic layers were dried over anhydrous sodium sulfate

and the volatiles were removed in vacuo giving 1,3-diphenyl-2-butylpropan-2-ol as a colorless oil. Yield: 258.6 mg (0.96 mmol, 80%); amount of recovered ketone: 36.0 mg (0.17 mmol, 14%).

Li_3Ce(*n*-Bu)$_6$(thf)$_4$ (3^{Ce}) and ketone/THF, then ”cerium turbo chloride” (Table 1, entry 12). A chilled solution of 3^{Ce} (158.4 mg, 0.2 mmol) in *n*-hexane (2 mL, –40 °C) was added dropwise to a pre-cooled mixture of 1,3-diphenylpropan-2-one (252.3 mg, 1.2 mmol, six equiv.) dissolved in THF (3 mL). After stirring for 5 min at –40 °C an equally cold solution of "cerium turbo chloride" (1.0 mmol, five equiv.) was added. The mixture was then stirred for another 30 min at –40 °C before it was quenched with a saturated aqueous solution of sodium carbonate, and the aqueous layer was extracted three times with diethyl ether. The combined organic layers were dried over anhydrous sodium sulfate and the volatiles were removed in vacuo giving 1,3-diphenyl-2-butylpropan-2-ol as a colorless oil. Yield: 292.2 mg (1.08 mmol, 90%); amount of recovered ketone: 2.3 mg (0.01 mmol, 1%).

Li_3Ce(*n*-Bu)$_6$(thf)$_4$ (3^{Ce}) and ketone/THF, then Sc(OTf)$_3$ (Table 1, entry 13). A chilled solution of 3^{Ce} (158.4 mg, 0.2 mmol) in *n*-hexane (2 mL, –40 °C) was added dropwise to a pre-cooled mixture of 1,3-diphenylpropan-2-one (252.3 mg, 1.2 mmol, six equiv.) dissolved in THF (3 mL). After stirring for five min at –40 °C an equally cold solution of scandium(III) triflate (1.0 mmol, five equiv.) in THF (2 mL) was added. The mixture was then stirred for another 30 min at –40 °C before it was quenched with a saturated aqueous solution of sodium carbonate, and the aqueous layer was extracted three times with diethyl ether. The combined organic layers were dried over anhydrous sodium sulfate and the volatiles were removed in vacuo giving 1,3-diphenyl-2-butylpropan-2-ol as a colorless oil. The yield could not be determined gravimetrically because the lithium triflate could not be removed completely by this work-up procedure; so only the NMR ratios of the alcohol and ketone are stated here: alcohol 62%, ketone 38%.

Li_3Ce(*n*-Bu)$_6$(thf)$_4$ (3^{Ce}) and ketone/THF, then $AlCl_3$ (Table 1, entry 14). A chilled solution of 3^{Ce} (158.4 mg, 0.2 mmol) in *n*-hexane (2 mL, –40 °C) was added dropwise to a pre-cooled mixture of 1,3-diphenylpropan-2-one (252.3 mg, 1.2 mmol, six equiv.) dissolved in THF (3 mL). After stirring for five min at –40 °C an equally cold solution of $AlCl_3$ (1.0 mmol, five equiv.) in THF (2 mL) was added. The mixture was then stirred for another 30 min at –40 °C before it was quenched with a saturated aqueous solution of sodium carbonate, and the aqueous layer was extracted three times with diethyl ether. The combined organic layers were dried over anhydrous sodium sulfate and the volatiles were removed in vacuo giving 1,3-diphenyl-2-butylpropan-2-ol as a colorless oil. Yield: 254.2 mg (0.95 mmol, 79%); amount of recovered ketone: 52.8 mg (0.25 mmol, 21%).

Li_3Ce(*n*-Bu)$_6$(thf)$_4$ (3^{Ce}) and ketone/THF/TMEDA (Table 1, entry 15). A chilled solution of 3^{Ce} (158.4 mg, 0.2 mmol) in *n*-hexane (2 mL, –40 °C) was added dropwise to a pre-cooled mixture of 1,3-diphenylpropan-2-one (252.3 mg, 1.2 mmol, six equiv.) and TMEDA (90.5 µL, 0.6 mmol, three equiv.) dissolved in THF (5 mL), and stirred for 30 min at –40 °C. The mixture was then quenched with a saturated aqueous solution of sodium carbonate, and the aqueous layer was extracted three times with

diethyl ether. The combined organic layers were dried over anhydrous sodium sulfate and the volatiles were removed in vacuo giving 1,3-diphenyl-2-butylpropan-2-ol as a colorless oil. Yield: 235.1 mg (0.88 mmol, 73%); amount of recovered ketone: 40.2 mg (0.19 mmol, 16%).

$Li_3Lu(n\text{-}Bu)_6(thf)_4$ (3^{Lu}) and ketone/THF (Table 1, entry 16). A chilled solution of $\mathbf{3^{Lu}}$ (100.6 mg, 0.12 mmol) in *n*-hexane (2 mL, –40 °C) was added dropwise to a pre-cooled solution of 1,3-diphenylpropan-2-one (153.4 mg, 0.73 mmol, six equiv.) dissolved in THF (2 mL) and stirred for 30 min at –40 °C. The mixture was then quenched with a saturated aqueous solution of sodium carbonate, and the aqueous layer was extracted three times with diethyl ether. The combined organic layers were dried over anhydrous sodium sulfate and the volatiles were removed in vacuo giving 1,3-diphenyl-2-butylpropan-2-ol as a colorless oil. Yield: 145.4 mg (0.54 mmol, 74%); amount of recovered ketone: 34.6 mg (0.16 mmol, 23%).

$Li_3Lu(n\text{-}Bu)_6(thf)_4$ (3^{Lu}) and ketone/THF/TMEDA (Table 1, entry 17). A chilled solution of $\mathbf{3^{Lu}}$ (165.4 mg, 0.2 mmol) in *n*-hexane (2 mL, –40 °C) was added dropwise to a pre-cooled solution of 1,3-diphenylpropan-2-one (252.3 mg, 1.2 mmol, six equiv.) and TMEDA (90.5 µL, 0.6 mmol, three equiv.) dissolved in THF (5 mL) and stirred for 30 min at –40 °C. The mixture was then quenched with a saturated aqueous solution of sodium carbonate, and the aqueous layer was extracted three times with diethyl ether. The combined organic layers were dried over anhydrous sodium sulfate and the volatiles were removed in vacuo giving 1,3-diphenyl-2-butylpropan-2-ol as a colorless oil. Yield: 141.7 mg (0.53 mmol, 44%); amount of recovered ketone: 141.6 mg (0.67 mmol, 56%).

$Li_3Y(n\text{-}Bu)_6(thf)_4$ (3^{Y}) and ketone/THF (Table 1, entry 18). A chilled solution of $\mathbf{3^{Y}}$ (93.7 mg, 0.13 mmol) in *n*-hexane (2 mL, –40 °C) was added dropwise to a pre-cooled solution of 1,3-diphenylpropan-2-one (160.9 mg, 0.73 mmol, six equiv.) dissolved in THF (2 mL) and stirred for 30 min at –40 °C. The mixture was then quenched with a saturated aqueous solution of sodium carbonate, and the aqueous layer was extracted three times with diethyl ether. The combined organic layers were dried over anhydrous sodium sulfate and the volatiles were removed in vacuo giving 1,3-diphenyl-2-butylpropan-2-ol as a colorless oil. Yield: 155.7 mg (0.58 mmol, 75%); amount of recovered ketone: 19.4 mg (0.09 mmol, 12%).

$Li_3Y(n\text{-}Bu)_6(thf)_4$ (3^{Y}) and ketone/THF @ ambient temperature (Table 1, entry 19). A chilled solution of $\mathbf{3^{Y}}$ (38.1 mg, 0.05 mmol) in *n*-hexane (2 mL, –40 °C) was added dropwise to a solution of 1,3-diphenylpropan-2-one (65.3 mg, 0.31 mmol, six equiv.) dissolved in THF (2 mL) and stirred for 30 min at ambient temperature. The mixture was then quenched with a saturated aqueous solution of sodium carbonate, and the aqueous layer was extracted three times with diethyl ether. The combined organic layers were dried over anhydrous sodium sulfate and the volatiles were removed in vacuo giving 1,3-diphenyl-2-butylpropan-2-ol as a colorless oil. Yield: 51.6 mg (0.19 mmol, 62%); amount of recovered ketone: 24.7 mg (0.12 mmol, 38%.).

***n*-BuLi and ketone/THF (Table 1, entry 20).** To a chilled solution of 1,3-diphenylpropan-2-one (420.5 mg, 2.0 mmol) dissolved in THF (5 mL, –40 °C) *n*-BuLi (2.5 M in hexanes, 800.0 µL, 2.0 mmol, one equiv.) was added dropwise and stirred for 30 min at –40 °C. The mixture was then quenched with a saturated aqueous solution of sodium carbonate, and the aqueous layer was extracted three times with diethyl ether. The combined organic layers were dried over anhydrous sodium sulfate and the volatiles were removed under reduced pressure giving 1,3-diphenyl-2-butylpropan-2-ol as a colorless oil. Yield: 268.1 mg (1.00 mmol, 50%); amount of recovered ketone: 209.2 mg (0.99 mmol, 50%).

X-Ray Crystallography

All crystals are extremely sensitive toward oxygen and moisture. The preparation for XRD analysis must be carried out under seamless cooling. The behavior at low temperatures was also very different, which causes the different measuring temperatures for the complexes in Tables S1, S2, and S3 below. For compound **2** a very small slot between 200 and 220 K had to be used for a successful preparation and measuring of the crystal. For most of the compounds disorder was observed and restraints have to be used for most of them.

Table S1. X-ray crystallographic parameters for complexes 1, 2, and 3^{Lu}

Compound	$Li_3CeMe_6(tmeda)_3$	$[Li(thf)_4][Ce(tBu)_4]$	$Li_3Lu(n\text{-}Bu)_6(thf)_4$
**Sample code**	**1**	**2**	**3^{Lu}**
**CCDC**	2069609	2069616	2069613
**Empirical formula**	$C_{24}H_{66}CeLi_3N_6$	$C_{32}H_{68}CeLiO_4$	$C_{40}H_{86}Li_3LuO_4$
**Formula weight**	599.77	663.92	826.87
**Temperature [K]**	150(2)	220(2)	173(2)
**Crystal system**	Trigonal	Monoclinic	Monoclinic
**Space group**	R-3c	Pc	$P2_1$
**a [Å]**	16.4893(12)	11.0681(16)	12.6114(17)
**b [Å]**	16.4893(12)	9.7385(14)	14.251(2)
**c [Å]**	26.526(3)	18.666(3)	12.9534(17)
**α [°]**	90.00	90.00	90.00
**β [°]**	90.00	89.994(3)	91.914(3)
**γ [°]**	120	90.00	90.00
**Volume [Å^3]**	6246.0(11)	2012.0(5)	2326.8(5)
**Z**	6	2	2
**ρ_{calc} [g/cm^3]**	0.957	1.096	1.180
**μ [mm^{-1}]**	1.109	1.157	2.153
**F(000)**	1914	706	876
**Crystal size [mm^3]**	0.298 × 0.242 × 0.195	0.457 x 0.448 x 0.274	0.528 × 0.381 × 0.278
**Radiation**	MoK_α (λ = 0.71073)	MoK_α (λ = 0.71073)	MoK_α (λ = 0.71073)
**Temperature [K]**	150	220	173
**Θ range for data collection [°]**	3.379 to 29.559	1.091 to 25.682	1.573 to 24.749
**Index ranges**	-22≤h≤15, -22≤k≤22, -35≤l≤35	-13≤h≤13, -11≤k≤11, -22≤l≤22	-14≤h≤14, -16≤k≤16, -15≤l≤15
**Reflections collected**	11031	40711	56503
**Independent reflections**	1930 [R_{int} = 0.0308]	7636 [R_{int} = 0.0319]	7964 [R_{int} = 0.0937]
**Data/restraints/ parameters**	1930 / 123 / 97	7636 / 1847 / 536	7964 / 2167 / 629
**Goodness-of-fit on F^2[a]**	1.030	1.028	1.034
**Final R indexes [I>=2σ (I)][b][c]**	R_1 = 0.0203, wR_2 = 0.0489	R_1 = 0.0299, wR_2 = 0.0649	R_1 = 0.0601, wR_2 = 0.1532
**Final R indexes [all data]**	R_1 = 0.0363, wR_2 = 0.0582	R_1 = 0.0414, wR_2 = 0.0713	R_1 = 0.0930, wR_2 = 0.1869
**Largest diff. peak/hole [e Å^{-3}]**	0.318 / -0.153	0.564 / -0.164	1.504 / -1.240

[a]GOF = $[\Sigma w(F_0^2 - F_c^2)^2 / (n_0 - n_p)]^{1/2}$. [b]R1 = $\Sigma(||F_0| - |F_c||) / \Sigma|F_0|$, $F_0 > 4\sigma(F_0)$. [c]$wR_2 = \{\Sigma[w(F_0^2 - F_c^2)^2 / \Sigma[w(F_0^2)^2]\}^{1/2}$.

Table S2. X-ray crystallographic parameters for complexes 4^{Ce}, 4^{Lu}, and 5

Compound	$Li_2Ce(n\text{-}Bu)_5(tmeda)_2$	$Li_2Lu(n\text{-}Bu)_5(tmeda)_2$	$LiLu(n\text{-}Bu)_3Cl(tmeda)_2$
**Sample code**	**4^{Ce}**	**4^{Lu}**	**5**
**CCDC**	2069615	2069610	2069611
**Empirical formula**	$C_{32}H_{77}CeLi_2N_4$	$C_{32}H_{77}Li_2LuN_4$	$C_{24}H_{59}ClLiLuN_4$
**Formula weight**	671.97	706.82	621.11
**Temperature [K]**	150(2)	100(2)	100(2)
**Crystal system**	Monoclinic	Monoclinic	Monoclinic
**Space group**	$P2_1/n$	$P2_1/c$	$P2_1/n$
**a [Å]**	12.9223(12)	21.2812(6)	14.962(4)
**b [Å]**	21.4375(19)	9.9334(3)	14.748(3)
**c [Å]**	14.6340(13)	20.0178(6)	16.608(3)
**α [°]**	90.00	90.00	90.00
**β [°]**	91.063(2)	110.6500(10)	116.449(4)
**γ [°]**	90.00	90.00	90.00
**Volume [Å^3]**	4053.2(6)	3959.8(2)	3281.1(12)
**Z**	4	4	4
**ρ_{calc} [g/cm^3]**	1.101	1.186	1.257
**μ [mm^{-1}]**	1.145	2.515	3.105
**F(000)**	1444	1496	1288
**Crystal size [mm^3]**	0.365 × 0.289 × 0.056	0.373 × 0.196 × 0.191	0.345 x 0.123 x 0.113
**Radiation**	MoK_α (λ = 0.71073)	MoK_α (λ = 0.71073)	MoK_α (λ = 0.71073)
**Temperature [K]**	150	100	100
**Θ range for data collection [°]**	1.685 to 27.910	2.045 to 31.655	1.945 to 31.565
**Index ranges**	-17≤h≤17, -28≤k≤28, -19≤l≤19	-29≤h≤31, -14≤k≤14, -27≤l≤29	-21≤h≤21, -21≤k≤21, -23≤l≤24
**Reflections collected**	62172	88118	73017
**Independent reflections**	9673 [R_{int} = 0.0549]	13298 [R_{int} = 0.0332]	10452 [R_{int} = 0.0865]
**Data/restraints/ parameters**	9673 / 195 / 458	13298 / 116 / 447	10452 / 0 / 323
**Goodness-of-fit on F^2[a]**	1.025	1.042	1.018
**Final R indexes [I>=2σ (I)][b][c]**	R_1 = 0.0387, wR_2 = 0.0760	R_1 = 0.0252, wR_2 = 0.0588	R_1 = 0.0344, wR_2 = 0.0657
**Final R indexes [all data]**	R_1 = 0.0584, wR_2 = 0.0845	R_1 = 0.0322, wR_2 = 0.0617	R_1 = 0.0593, wR_2 = 0.0727
**Largest diff. peak/hole [e Å^{-3}]**	1.496 / -1.014	2.127 / -0.830	2.165 / -0.977

[a]GOF = $[\Sigma w(F_0^2 - F_c^2)^2 / (n_0 - n_p)]^{1/2}$. [b]R1 = $\Sigma(||F_0| - |F_c||) / \Sigma|F_0|$, $F_0 > 4\sigma(F_0)$. [c]$wR_2 = \{\Sigma[w(F_0^2 - F_c^2)^2 / \Sigma[w(F_0^2)^2]\}^{1/2}$.

Table S3. X-ray crystallographic parameters for complexes 6, 7, and 8

Compound	$Li_3Ce_2(ONep)_9(HONep)_2(thf)$	$Li_4[OC(=CHPh)(CH_2Ph)]_4(thf)_4$	$Li_8[OCMe_2(n\text{-}Bu)]_6Cl_2(thf)_6$
**Sample code**	**6**	**7**	**8**
**CCDC**	2069612	2069617	2069614
**Empirical formula**	$C_{59}H_{131}Ce_2Li_3O_{12}$	$C_{84}H_{100}Li_4O_{10}$	$C_{66}H_{138}Cl_2Li_8O_{12}$
**Formula weight**	1333.69	1297.39	1250.18
**Temperature [K]**	100(2)	100(2)	173(2)
**Crystal system**	Monoclinic	Tetragonal	Triclinic
**Space group**	$P2_1/c$	$I4_1/a$	P-1
**a [Å]**	12.0469(12)	17.7858(11)	11.6640(4)
**b [Å]**	22.287(2)	17.7858(11)	14.1661(5)
**c [Å]**	27.095(3)	50.026(5)	14.2938(5)
**α [°]**	90.00	90.00	62.4460(10)
**β [°]**	93.595(2)	90.00	73.8420(10)
**γ [°]**	90.00	90.00	74.2460(10)
**Volume [Å^3]**	7260.4(12)	15825(2)	1982.74(12)
**Z**	4	8	1
**ρ_{calc} [g/cm^3]**	1.218	1.089	1.047
**μ [mm^{-1}]**	1.287	0.069	0.132
**F(000)**	2816	5568	688
**Crystal size [mm^3]**	0.224 x 0.174 x 0.050	0.251 × 0.227 × 0.218	0.429 × 0.135 × 0.134
**Radiation**	MoK_α (λ = 0.71073)	MoK_α (λ = 0.71073)	MoK_α (λ = 0.71073)
**Temperature [K]**	100	100	173
**Θ range for data collection [°]**	1.506 to 29.159	1.215 to 25.309	1.633 to 25.082
**Index ranges**	-15≤h≤16, -30≤k≤30, -36≤l≤37	-14≤h≤21, -20≤k≤20, -60≤l≤60	-13≤h≤13, -16≤k≤16, -17≤l≤17
**Reflections collected**	82404	50086	44374
**Independent reflections**	19554 [R(int) = 0.0655]	7224 [R_{int} = 0.0717]	7033 [R_{int} = 0.0472]
**Data/restraints/ parameters**	19554 / 1075 / 843	7224 / 150 / 466	7033 / 36 / 406
**Goodness-of-fit on F^2[a]**	1.034	1.021	1.038
**Final R indexes [I>=2σ (I)][b][c]**	R_1 = 0.0415, wR_2 = 0.0870	R_1 = 0.0565, wR_2 = 0.1450	R_1 = 0.0532, wR_2 = 0.1384
**Final R indexes [all data]**	R_1 = 0.0652, wR_2 = 0.0998	R_1 = 0.0927, wR_2 = 0.1682	R_1 = 0.0698, wR_2 = 0.1532
**Largest diff. peak/hole [e Å^{-3}]**	1.701 / -0.657	0.334 / -0.372	0.983 / -0.367

[a]GOF = $[\Sigma w(F_0^2 - F_c^2)^2 / (n_0 - n_p)]^{1/2}$. [b]R1 = $\Sigma(||F_0| - |F_c||) / \Sigma|F_0|$, $F_0 > 4\sigma(F_0)$. [c]$wR_2 = \{\Sigma[w(F_0^2 - F_c^2)^2 / \Sigma[w(F_0^2)^2]\}^{1/2}$.

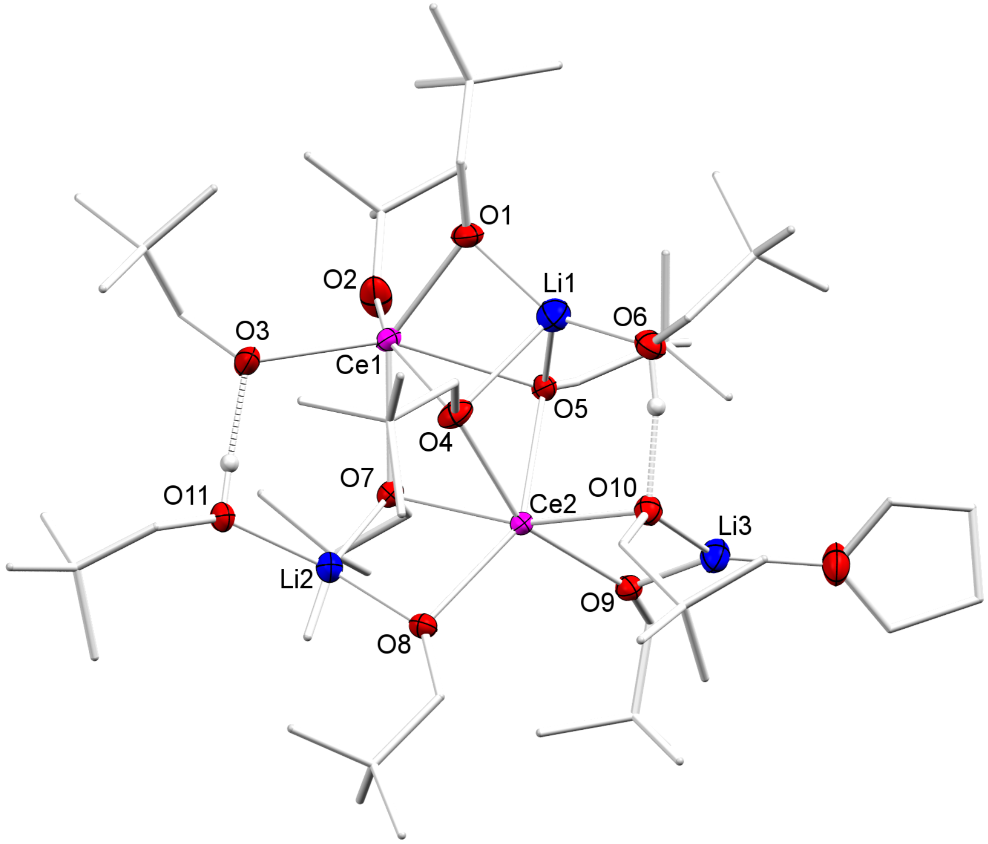

Figure S1. Crystal structure of $Li_3Ce_2(OCH_2tBu)_9(HOCH_2tBu)_2(thf)$ (**6**) with atomic displacement ellipsoids set at 50% probability. Hydrogen atoms except those engaging in hydrogen bridges are omitted for clarity. The neopentoxy and thf carbon atoms are shown in a ball-and-stick representation. Selected interatomic distances [Å] for **6**: Ce1–O1 2.327(2), Ce1–O2 2.196(2), Ce1–O3 2.337(2), Ce1–O4 2.585(2), Ce1–O5 2.608(2), Ce1–O7 2.496(2), Ce2–O4 2.393(2), Ce2–O5 2.421(2), Ce2–O7 2.472(2), Ce2–O8 2.354(2), Ce2–O9 2.320(2), Ce2–O10 2.447(2).

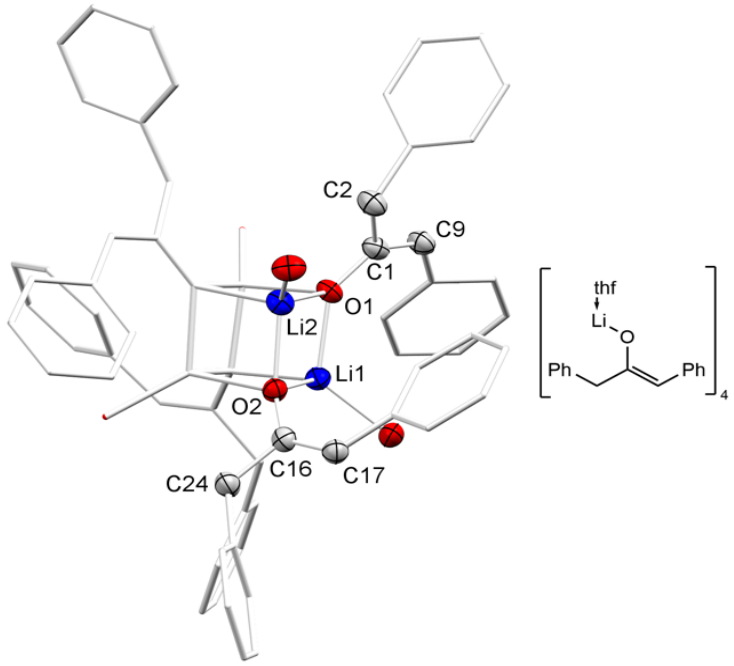

Figure S2. Crystal structure of $Li_4[OC(=CHPh)CH_2Ph]_4(thf)_4$ (**7**) with atomic displacement ellipsoids set at 50% probability. Hydrogen atoms, part of the THF molecules, and lattice THF are omitted for clarity. The second part of the complex is created by symmetry. Selected interatomic distances [Å] and angles [°] for **7**: Li1–O1 1.934(4), Li1–O2 1.991(4), Li2–O1 2.028(4), Li2–O2 1.931(4), Li1–O2' 2.036(4), O1–Li2' 1.985(4), C1–C2 1.528(3), C1–C9 1.349(3), C16–C17 1.352(3), C16–C24 1.521(3), O1–Li1–O2 94.25(15), Li1–O1–Li2 84.56(15).

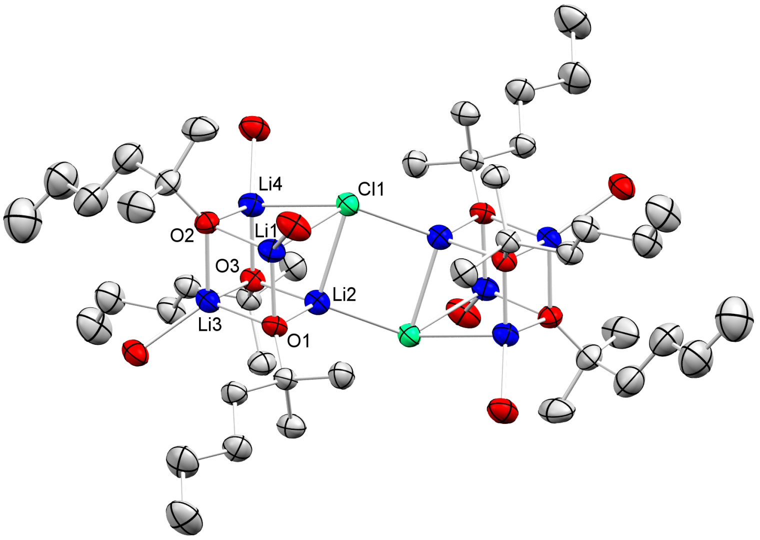

Figure S3. Crystal structure of $Li_8[OCMe_2(n\text{-}Bu)]_6Cl_2(thf)_6$ (**8**) with atomic displacement ellipsoids set at 50% probability. Hydrogen atoms and THF carbon atoms are omitted for clarity. Selected interatomic distances [Å] and angles [°] for **8**: Li1–O1 1.892(4), Li1–O2 1.932(4), Li1–Cl1 2.533(4), Li2–O1 1.926(4), Li2–O3 1.932(4), Li2–Cl1 2.517(4), Li2–Cl1' 2.517(4), Li3–O1 1.974(4), Li3–O2 1.962(4), Li3–O3 1.957(4), Li4–O2 1.941(4), Li4–O3 1.893(4), Li4–Cl1 2.516(4), Li1–O1–Li2 92.59(16), Li1–O1–Li3 83.67(16), Li2–O1–Li3 83.24(16), Cl1–Li4–O2 96.05(1), O2–Li4–O3 97.68(1), Cl1–Li4–O3 99.82(1), O1–Li3–O2 94.84(1), O1–Li3–O3 95.37(1), O2–Li3–O3 94.89(1), Li1–Cl1–Li2 66.27(1), Li1–Cl1–Li4 66.73(1), Li2–Cl1–Li4 66.44(1), Cl1–Li2–Cl1' 98.06(1), Li2–Cl1–Li2' 81.94(1).

NMR Spectra

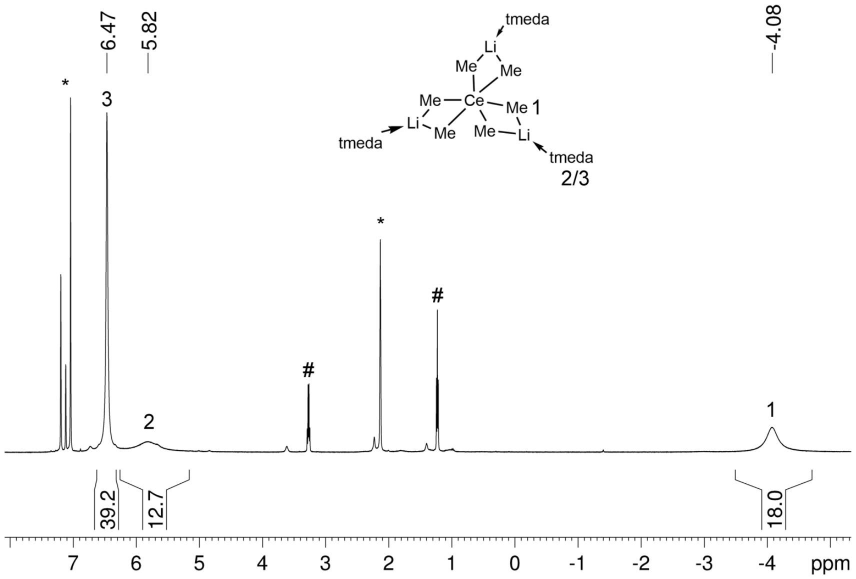

Figure S4. ^{1}H NMR spectrum of $Li_3CeMe_6(tmeda)_3$ (**1**) (500.13 MHz, toluene-d_8, 233 K) solvent residual signals are marked with *. The signals of non-coordinated TMEDA are marked with #.

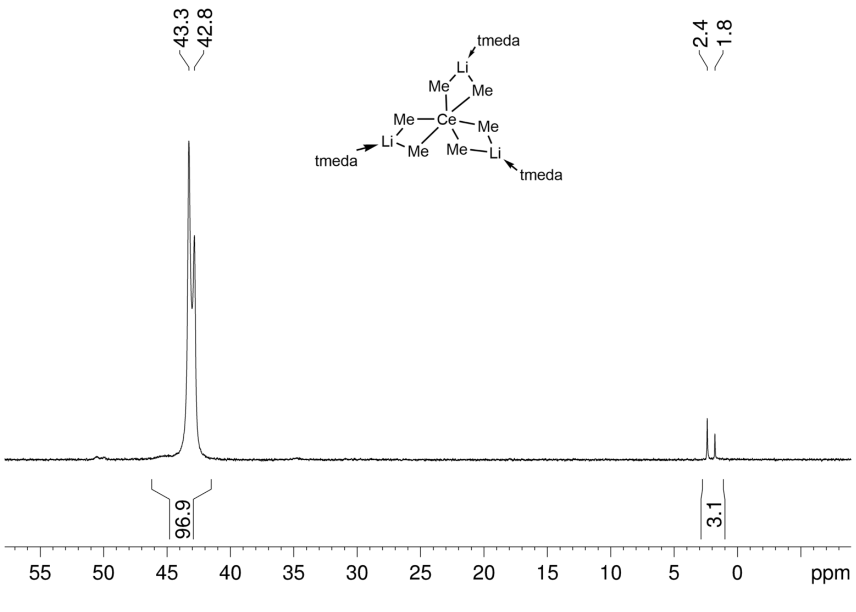

Figure S5. ^{7}Li NMR spectrum of $Li_3CeMe_6(tmeda)_3$ (**1**) (194.37 MHz, toluene-d_8, 233 K).

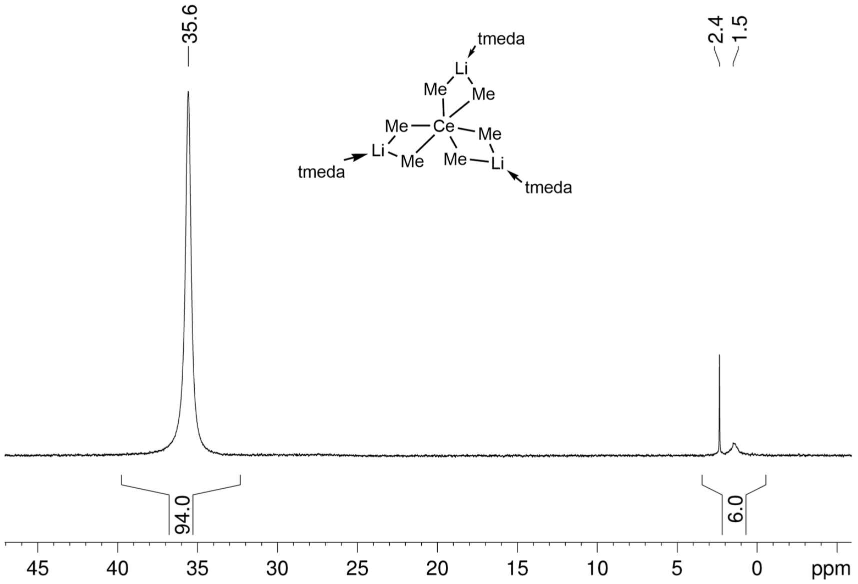

Figure S6. ^{7}Li NMR spectrum of $Li_3CeMe_6(tmeda)_3$ (**1**) (194.37 MHz, THF-d_8, 233 K).

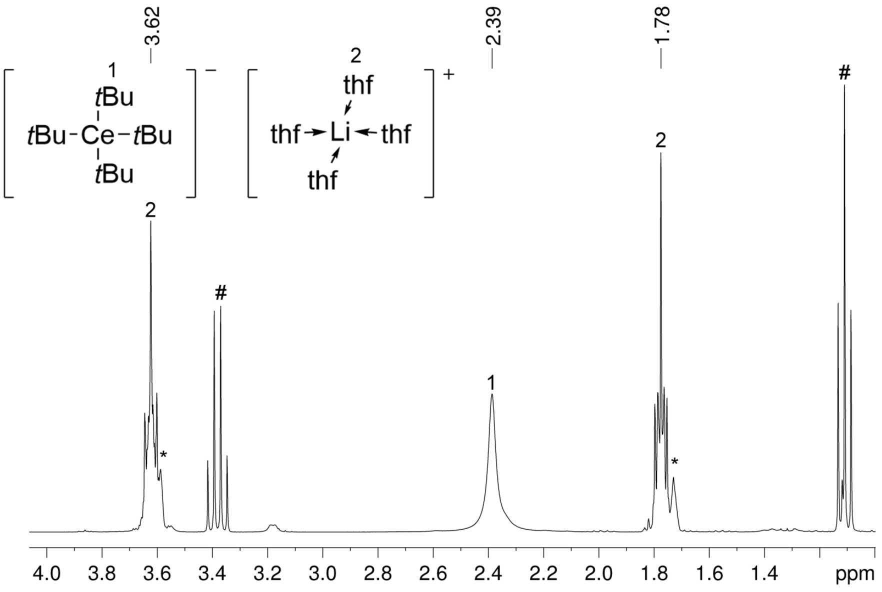

Figure S7. ^{1}H NMR spectrum of $[Li(thf)_4][Ce(t\text{-}Bu)_4]$ (**2**) (300.13 MHz, THF-d_8, 299 K). Solvent residual signals are marked with *. Residual Et_2O is marked with #.

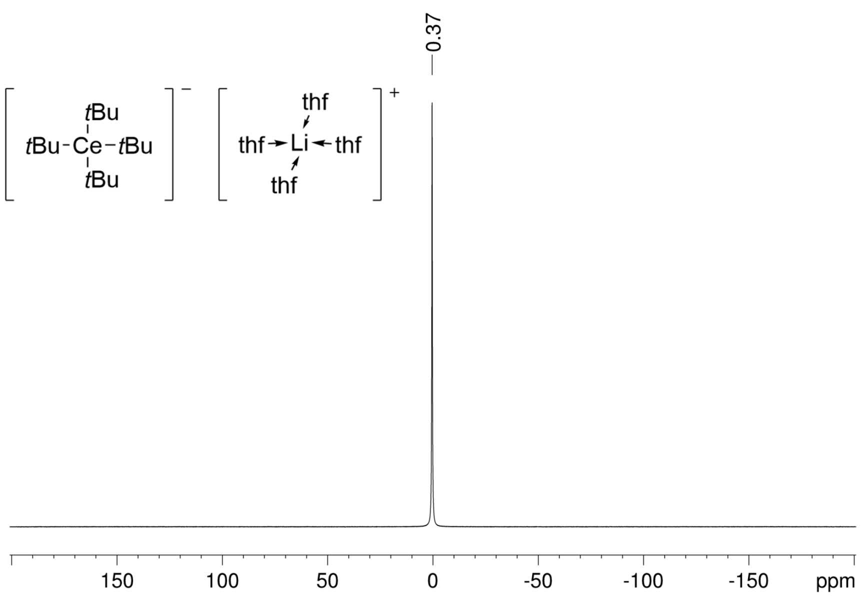

Figure S8. ^{7}Li NMR spectrum of $[Li(thf)_4][Ce(t\text{-}Bu)_4]$ (**2**) (116.64 MHz, THF-d_8, 299 K).

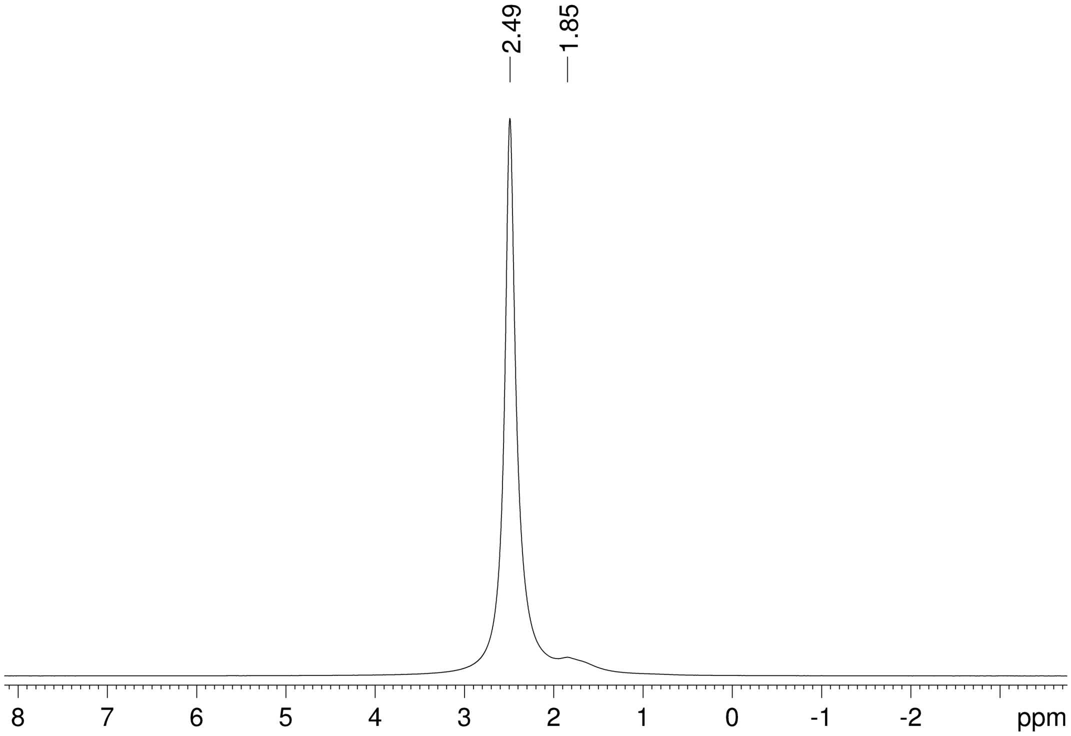

Figure S9. ^{7}Li NMR spectrum of *n*-BuLi (194.37 MHz, toluene-d_8, 233 K).

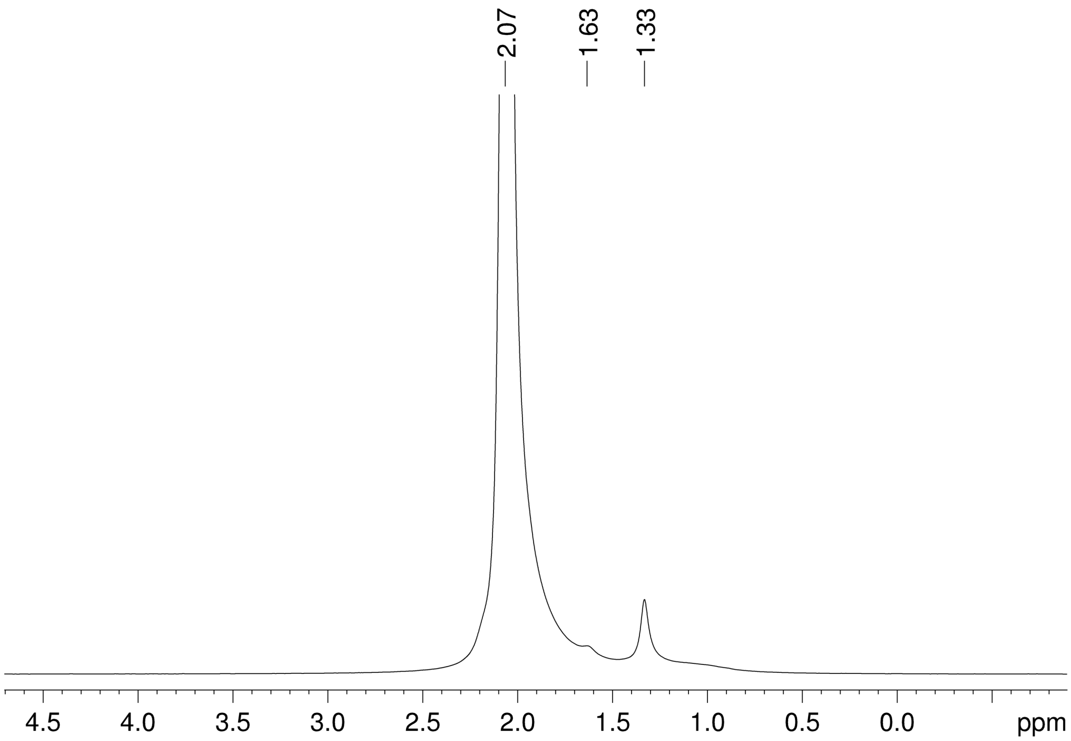

Figure S10. ^{7}Li NMR spectrum of *n*-BuLi + 1 equiv. of THF (194.37 MHz, toluene-d_8, 233 K).

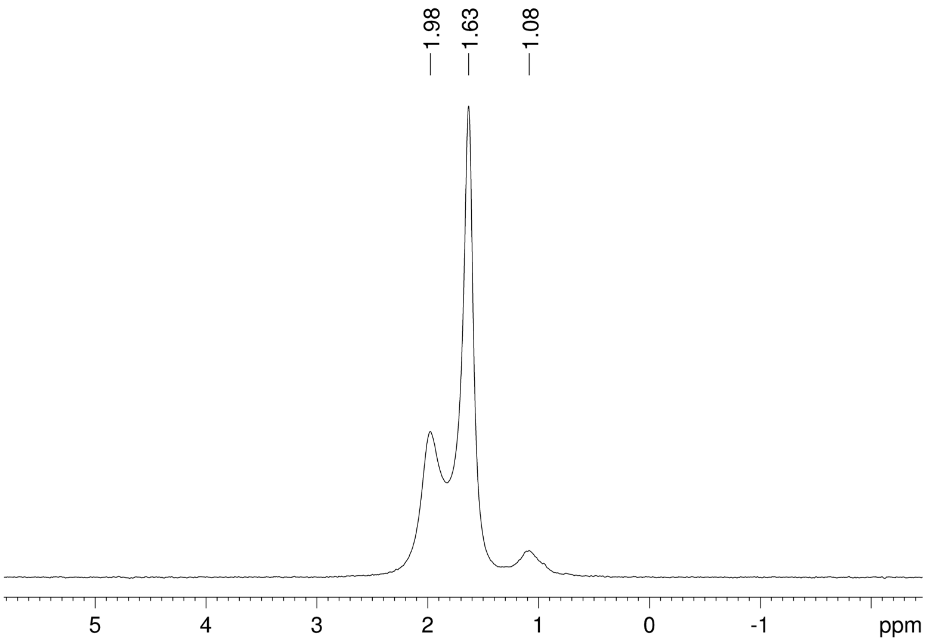

Figure S11. ^{7}Li NMR spectrum of *n*-BuLi (194.37 MHz, THF-d_8, 233 K).

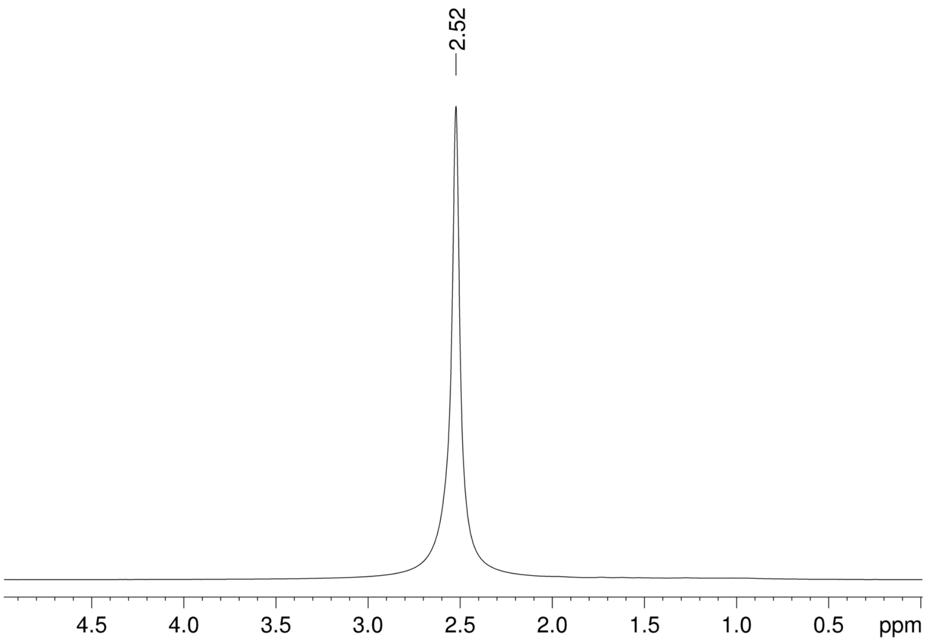

Figure S12. ^{7}Li NMR spectrum of *n*-BuLi + 1 equiv. of TMEDA (194.37 MHz, toluene-d_8, 233 K).

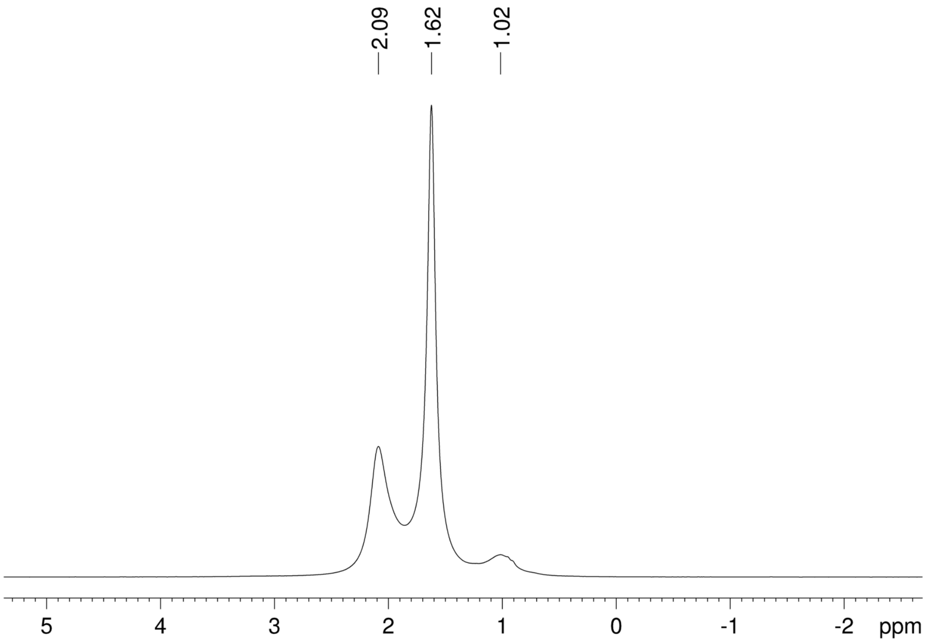

Figure S13. ^{7}Li NMR spectrum of *n*-BuLi + 1 equiv. of TMEDA (194.37 MHz, THF-d_8, 233 K).

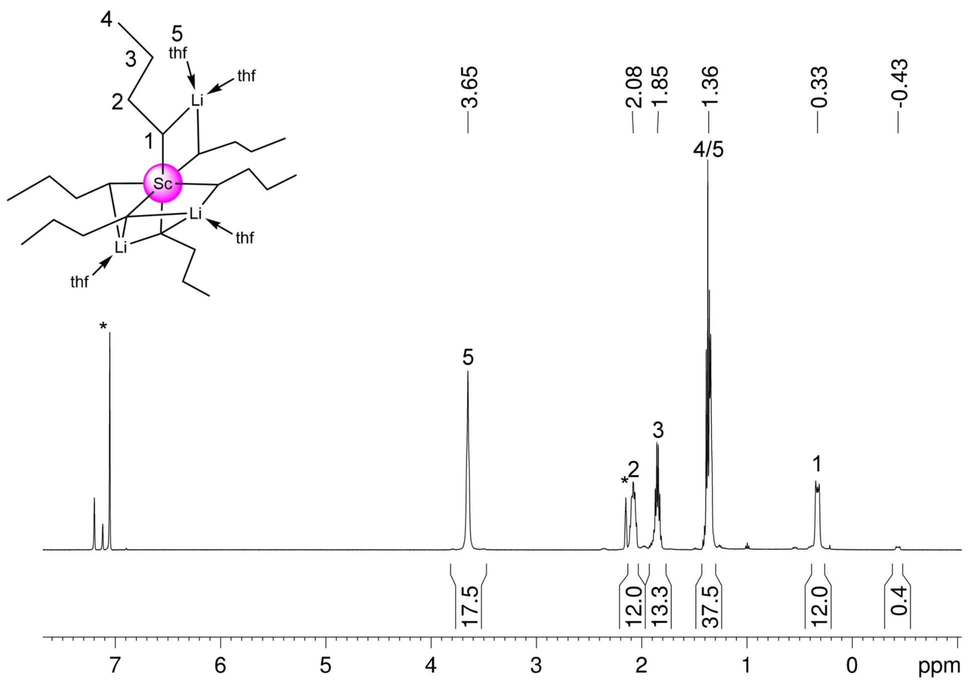

Figure S14. ^{1}H NMR spectrum of $Li_3Sc(n\text{-}Bu)_6(thf)_4$ (**3**^{Sc}) (500.13 MHz, toluene-d_8, 233 K). Solvent residual signals are marked with *.

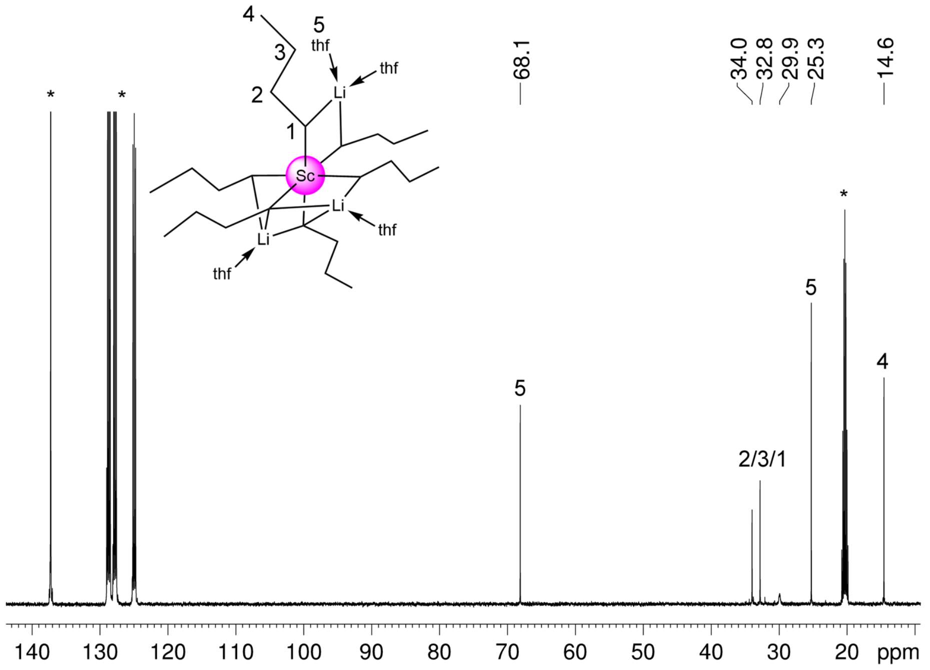

Figure S15. $^{13}C\{^1H\}$ NMR spectrum of $Li_3Sc(n\text{-}Bu)_6(thf)_4$ (**3**^{Sc}) (125.76 MHz, toluene-d_8, 233 K). Solvent residual signals are marked with *.

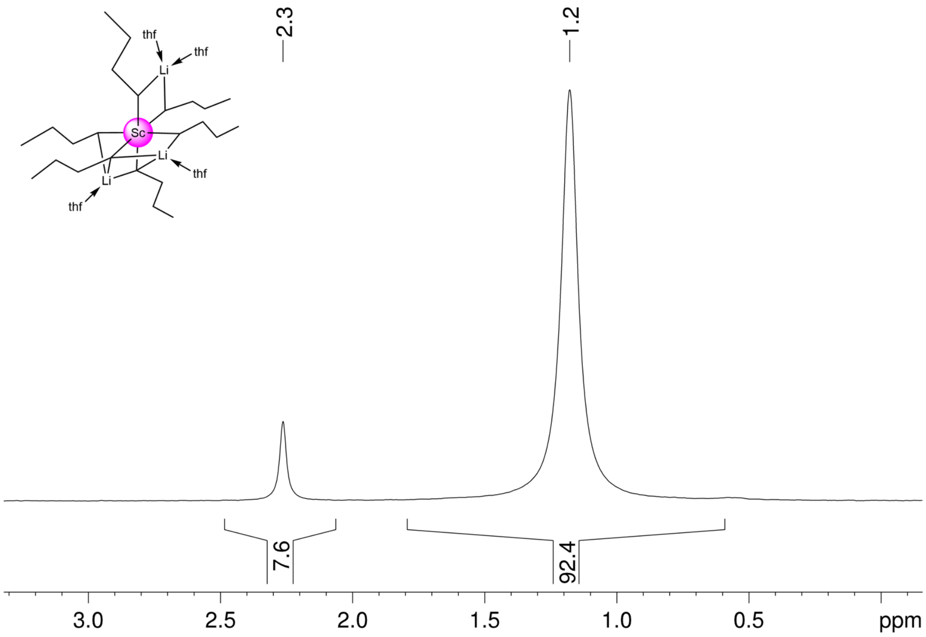

Figure S16. ^{7}Li NMR spectrum of $Li_3Sc(n\text{-}Bu)_6(thf)_4$ (**3^{Sc}**) (194.37 MHz, toluene-d_8, 233 K), showing free *n*-BuLi(thf) at 2.3 ppm and the mixed scandium–lithium complex at 1.2 ppm.

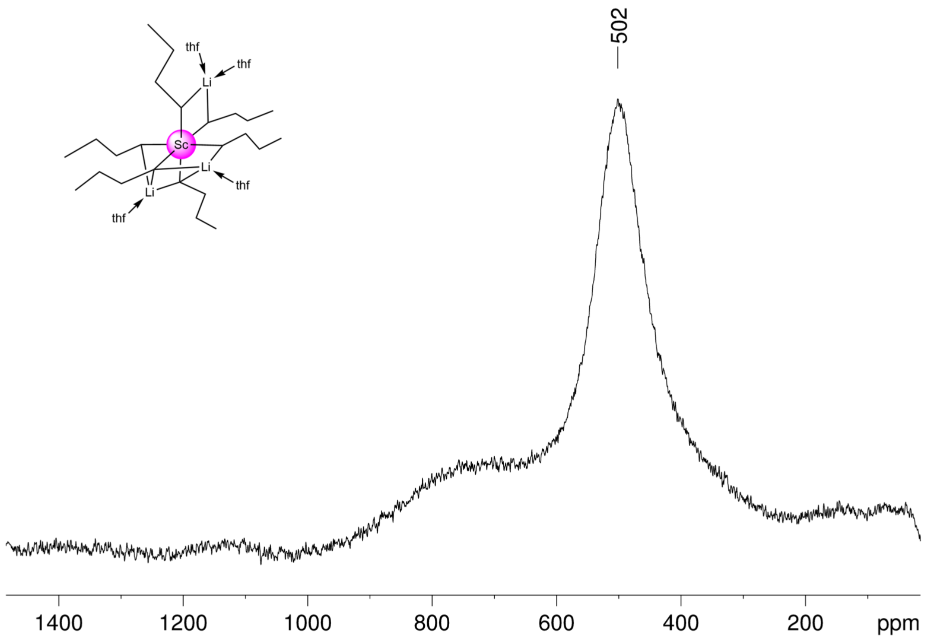

Figure S17. ^{45}Sc NMR spectrum of $Li_3Sc(n\text{-}Bu)_6(thf)_4$ (**3^{Sc}**) (121.49 MHz, toluene-d_8, 233 K), showing a main signal at 502 ppm with a line width at half height of 11787 Hz.

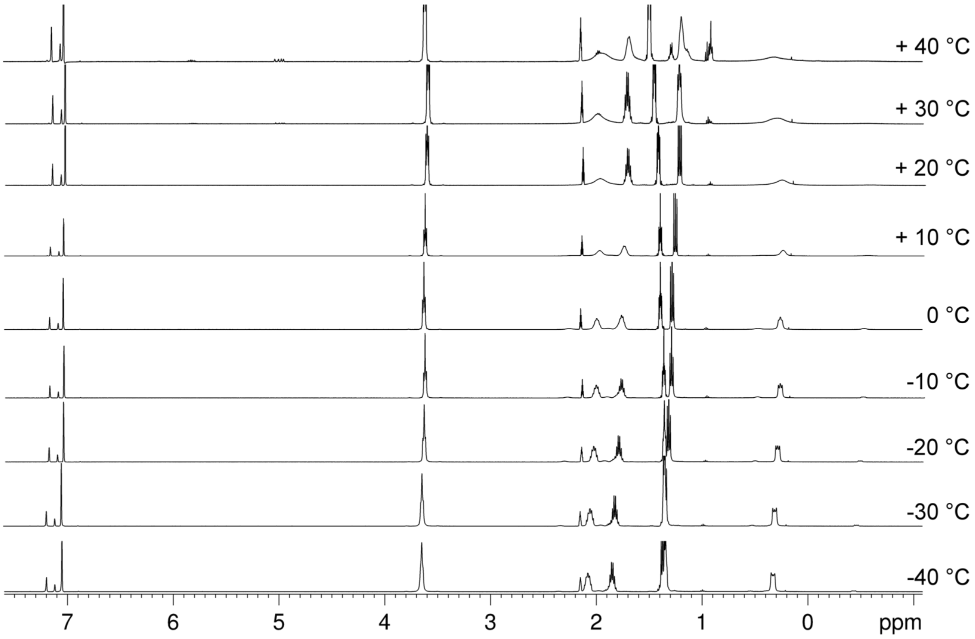

Figure S18. VT ^{1}H NMR spectra of $Li_3Sc(n\text{-}Bu)_6(thf)_4$ ($\mathbf{3^{Sc}}$) (500.13 MHz, toluene-d_8, 233 K-313 K) showing a slight amount of the decomposition product 1-butene at 5.82 and 4.99 ppm at +30 °C and +40 °C.

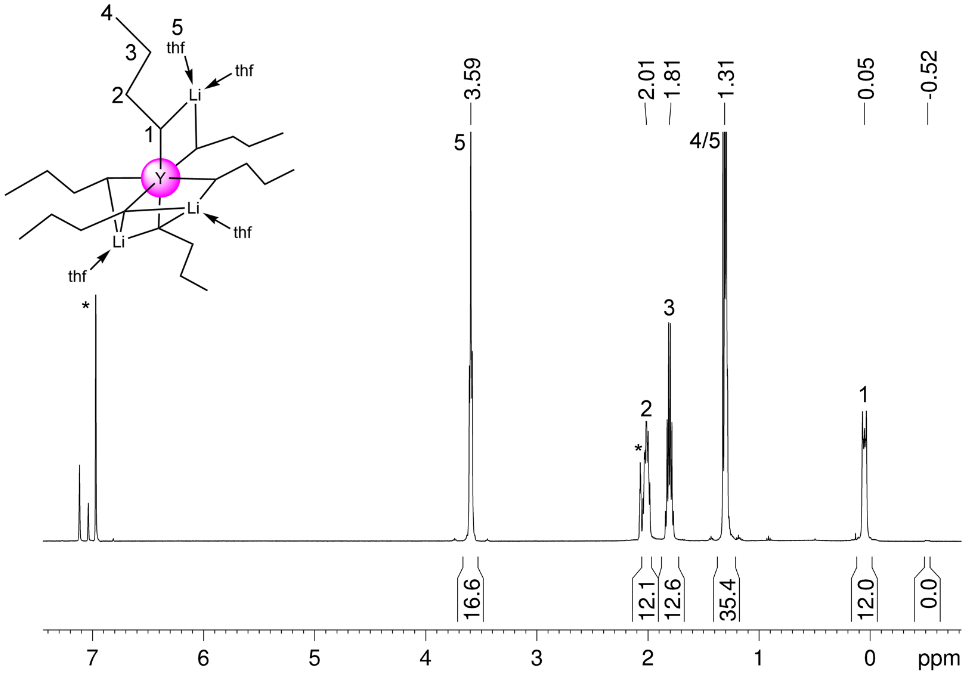

Figure S19. ^{1}H NMR spectrum of $Li_3Y(n\text{-}Bu)_6(thf)_4$ ($\mathbf{3^{Y}}$) (500.13 MHz, toluene-d_8, 233 K). Solvent residual signals are marked with *.

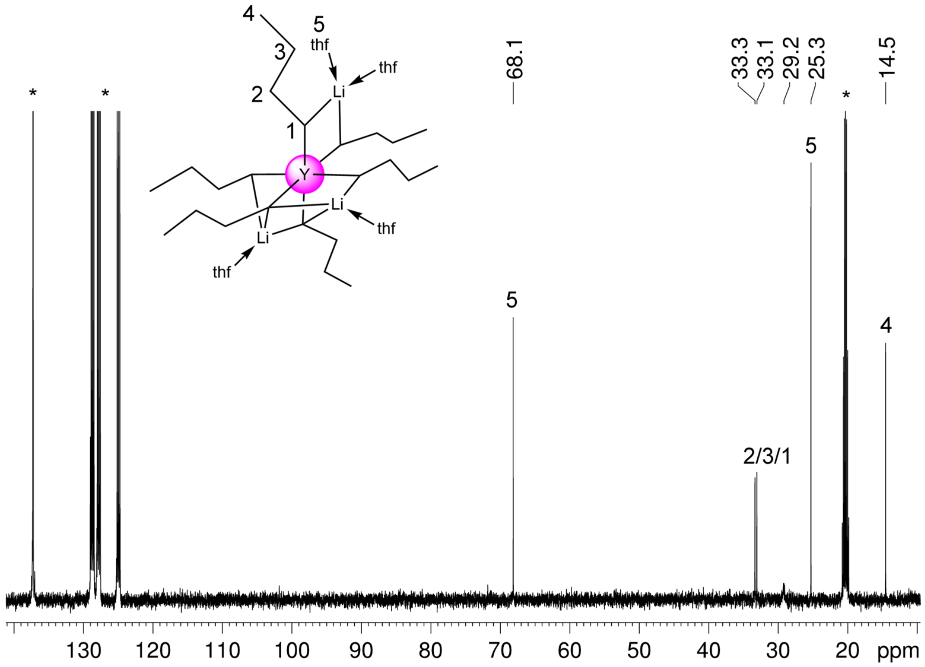

Figure S20. $^{13}C\{^{1}H\}$ NMR spectrum of $Li_3Y(n\text{-}Bu)_6(thf)_4$ (**3**$^{\mathbf{Y}}$) (125.76 MHz, toluene-d_8, 233 K). Solvent residual signals are marked with *.

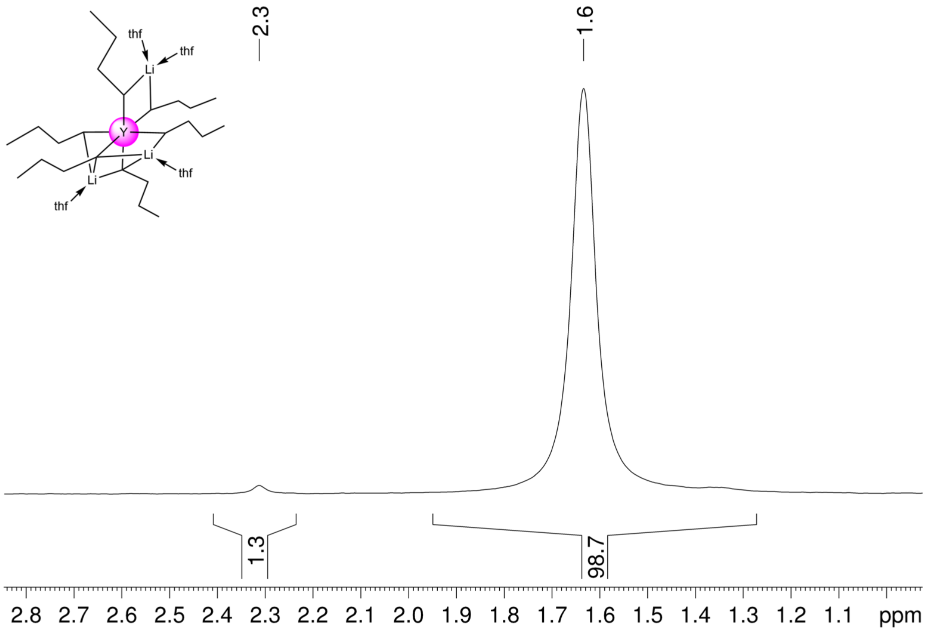

Figure S21. ^{7}Li NMR spectrum of $Li_3Y(n\text{-}Bu)_6(thf)_4$ (**3**$^{\mathbf{Y}}$) (194.37 MHz, toluene-d_8, 233 K), showing free *n*-BuLi(thf) at 2.3 ppm and the mixed yttrium–lithium complex at 1.6 ppm.

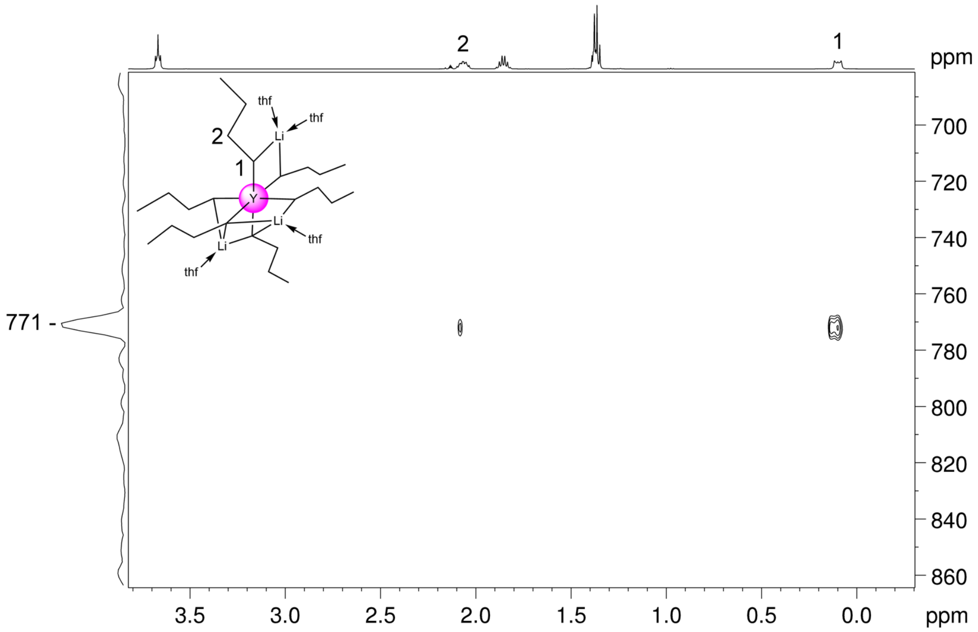

Figure S22. ^{1}H-^{89}Y HSQC NMR spectrum of $Li_3Y(n\text{-}Bu)_6(thf)_4$ (**3**$^{\mathbf{Y}}$) (500.13/24.51 MHz, toluene-d_8, 233 K), showing a single yttrium signal at 771 ppm with a line width at half height of 143 Hz.

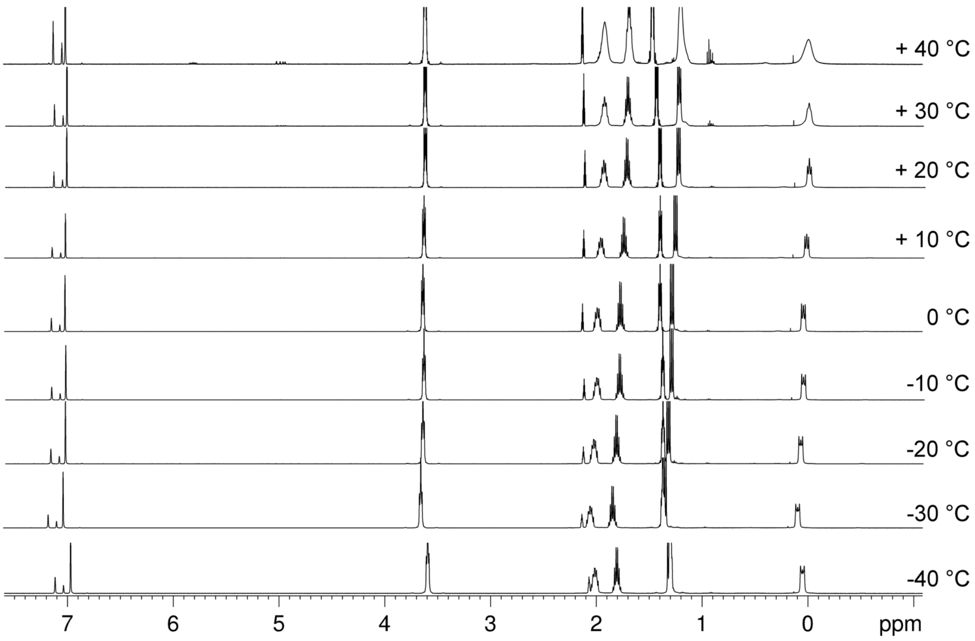

Figure S23. VT ^{1}H NMR spectra of $Li_3Y(n\text{-}Bu)_6(thf)_4$ (**3**$^{\mathbf{Y}}$) (500.13 MHz, toluene-d_8, 233 K-313 K), showing a slight amount of the decomposition product 1-butene at 5.82 and 4.99 ppm at +30 °C and +40 °C.

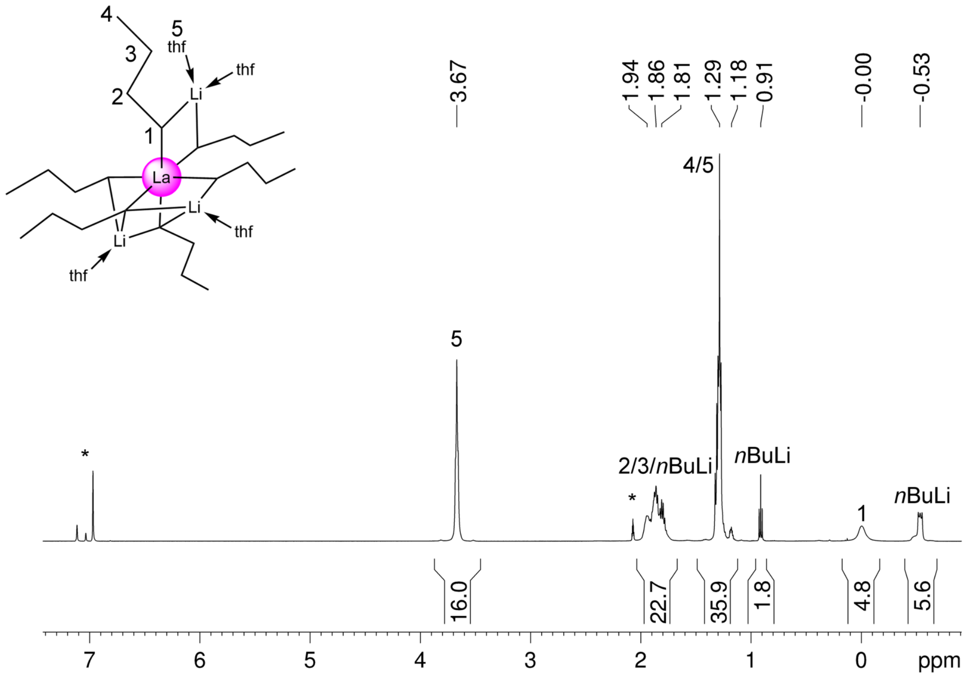

Figure S24. ^{1}H NMR spectrum of $Li_3La(n\text{-}Bu)_6(thf)_4$ (**3^{La}**) (500.13 MHz, toluene-d_8, 233 K). Solvent residual signals are marked with *.

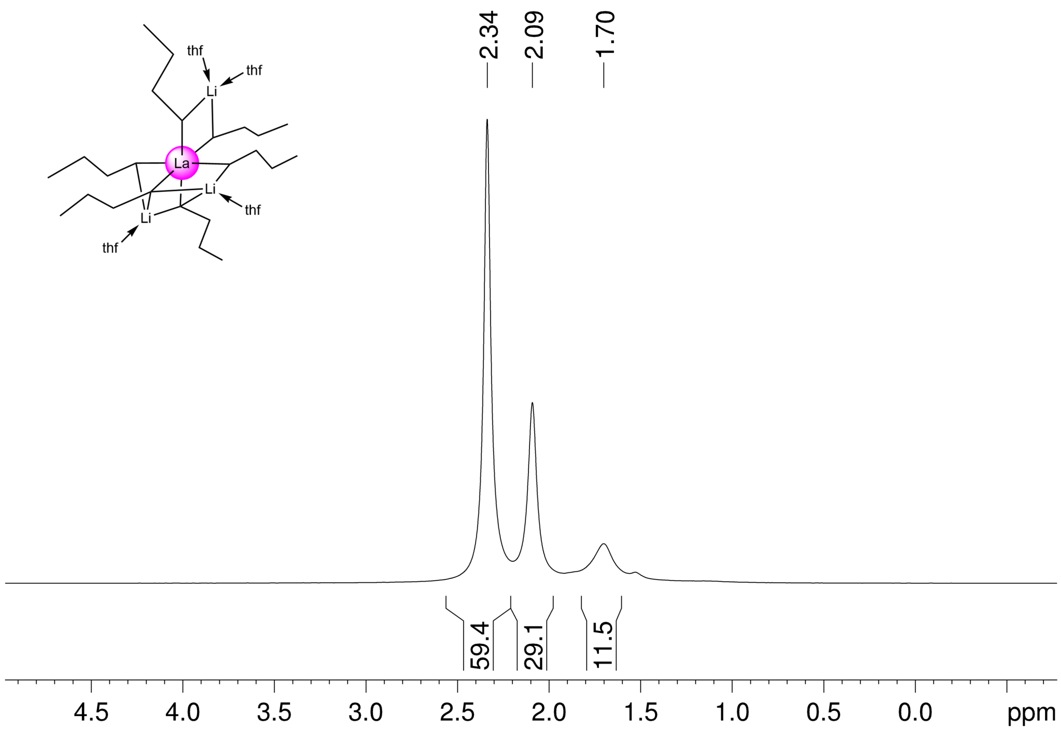

Figure S25. ^{7}Li NMR spectrum of $Li_3La(n\text{-}Bu)_6(thf)_4$ (**3^{La}**) (194.37 MHz, toluene-d_8, 233 K), showing mainly *n*-BuLi(thf).

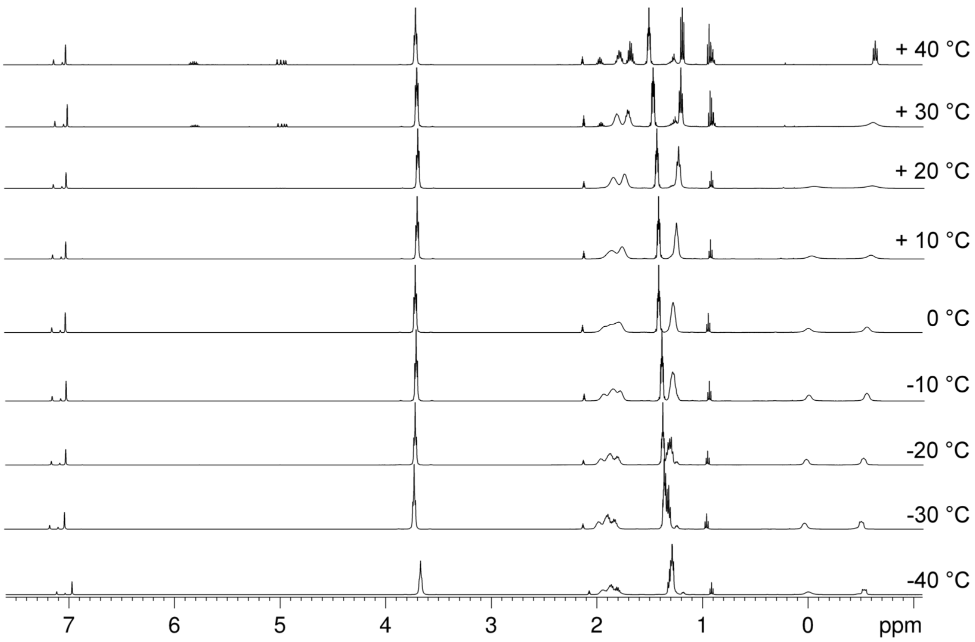

Figure S26. VT ^{1}H NMR spectra of $Li_3La(n\text{-}Bu)_6(thf)_4$ ($\mathbf{3^{La}}$) (500.13 MHz, toluene-d_8, 233 K-313 K), showing a slight amount of the decomposition product of 1-butene at 5.82 and 4.99 ppm at +30 °C and +40 °C.

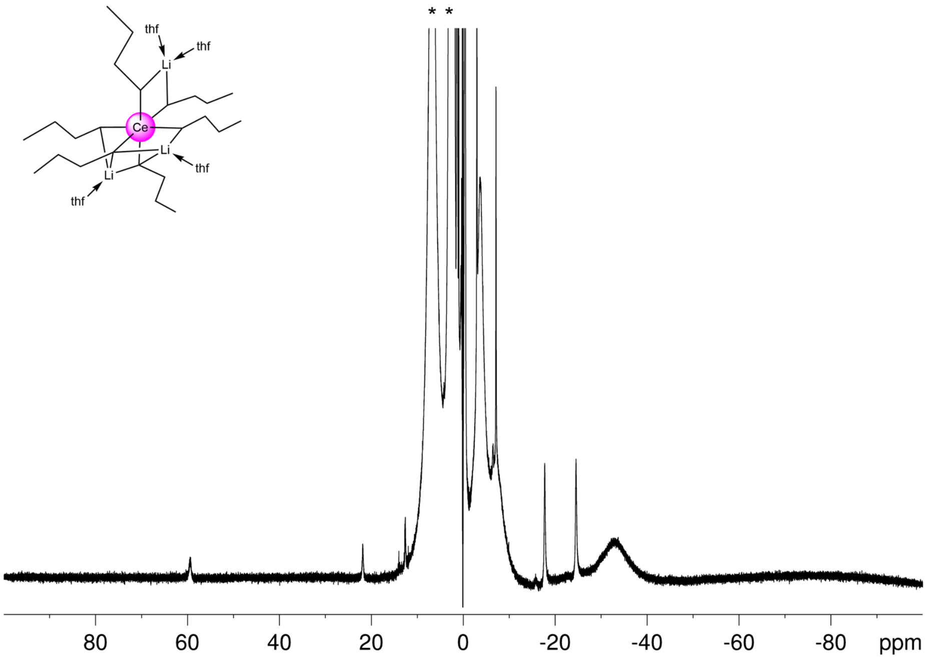

Figure S27. Wide ^{1}H NMR spectrum of $Li_3Ce(n\text{-}Bu)_6(thf)_4$ ($\mathbf{3^{Ce}}$) (500.13 MHz, toluene-d_8, 233 K). Solvent residual signals are marked with *. Except for the sharp *n*-BuLi(thf) signals, resonance assignment is not conclusive.

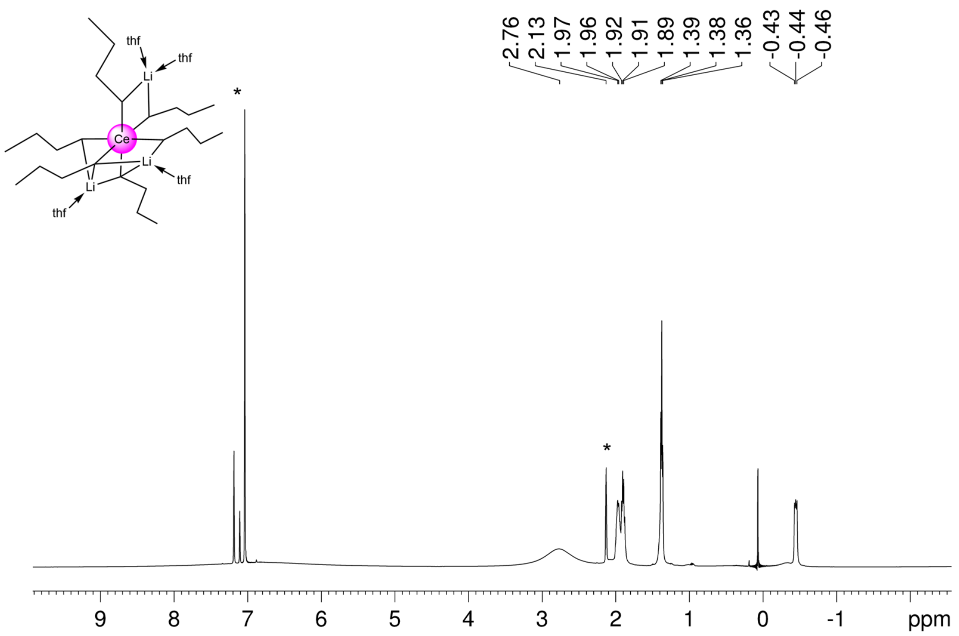

Figure S28. Narrow ^{1}H NMR spectrum of $Li_3Ce(n\text{-}Bu)_6(thf)_4$ ($\mathbf{3}^{Ce}$) (500.13 MHz, toluene-d_8, 233 K). Solvent residual signals are marked with *. The sharp signals can all be assigned to free *n*-BuLi or *n*-BuLi(thf).

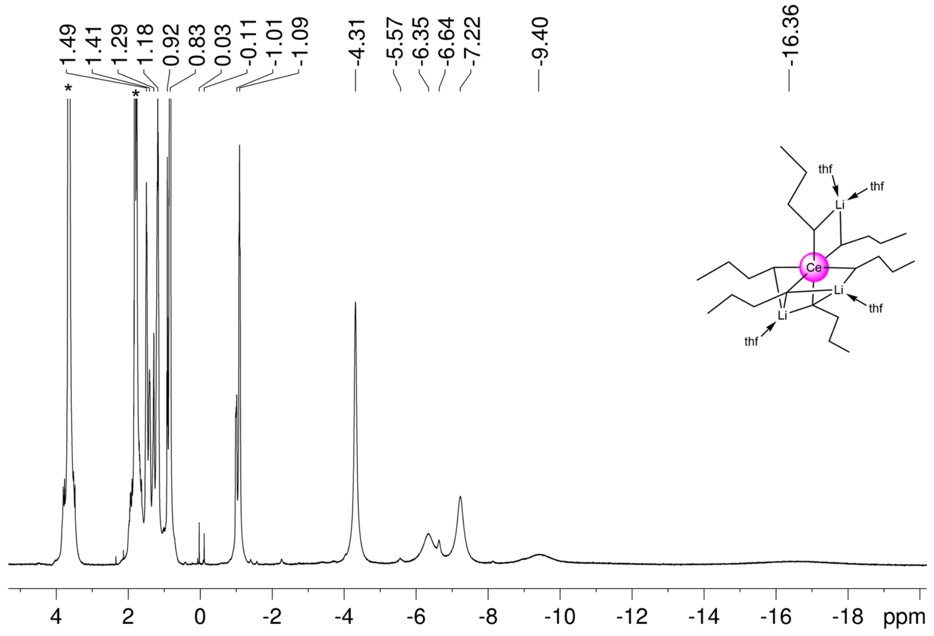

Figure S29. ^{1}H NMR spectrum of $Li_3Ce(n\text{-}Bu)_6(thf)_4$ ($\mathbf{3}^{Ce}$) (500.13 MHz, THF-d_8, 233 K). Solvent residual signals are marked with *.

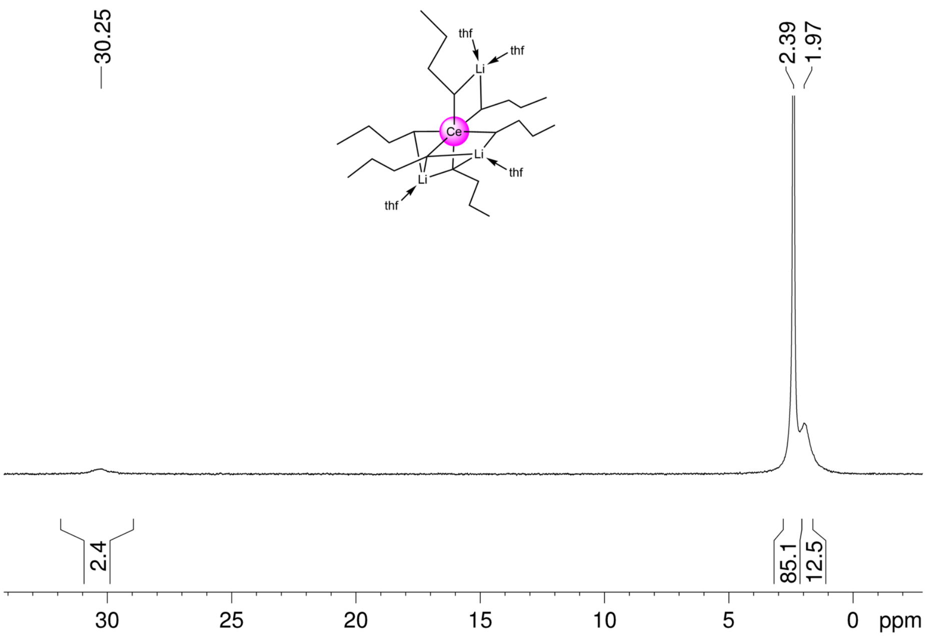

Figure S30. ^{7}Li NMR spectrum of $Li_3Ce(n\text{-}Bu)_6(thf)_4$ (**3**^{Ce}) (194.37 MHz, toluene-d_8, 233 K), showing the *n*-BuLi(thf) at 2.4 and 2.0 ppm and a mixed cerium–lithium species at 30.3 ppm.

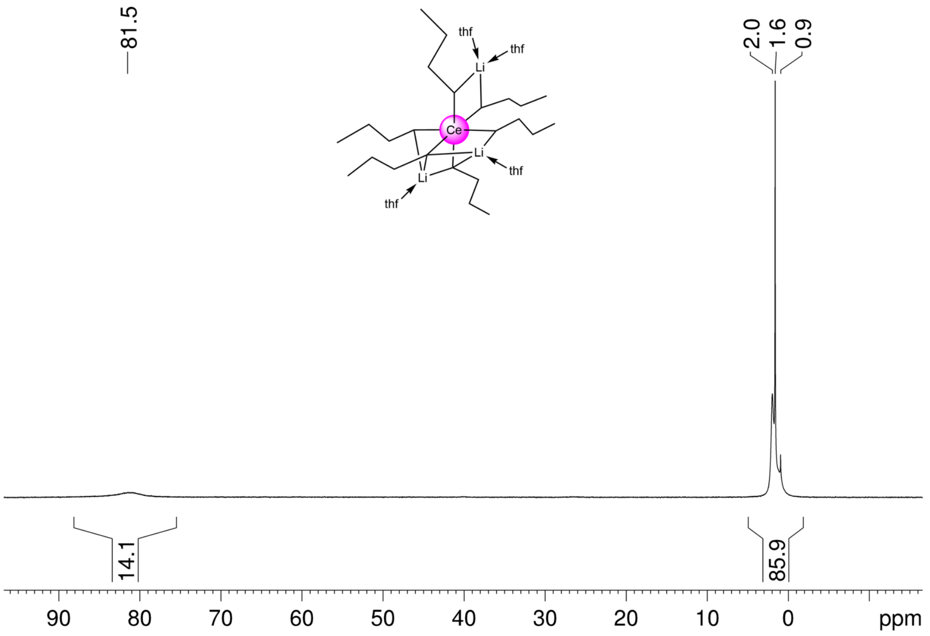

Figure S31. ^{7}Li NMR spectrum of $Li_3Ce(n\text{-}Bu)_6(thf)_4$ (**3**^{Ce}) (194.37 MHz, THF-d_8, 233 K), showing the *n*-BuLi(thf) at 2.0,1.6 and 0.9 ppm and a mixed cerium–lithium species at 81.5 ppm.

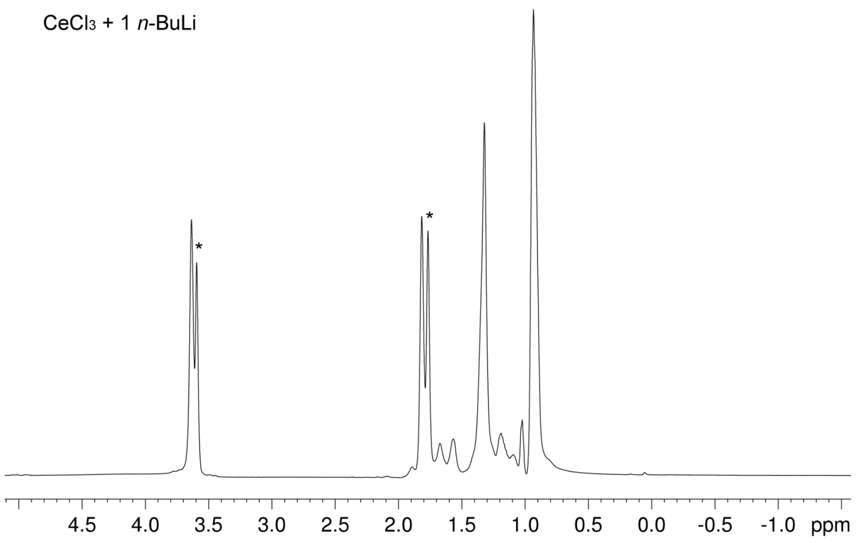

Figure S32. ^{1}H NMR spectrum of $CeCl_3$(thf) + 1 equiv. of *n*-BuLi (500.13 MHz, THF-d_8, 233 K). Solvent residual signals are marked with *.

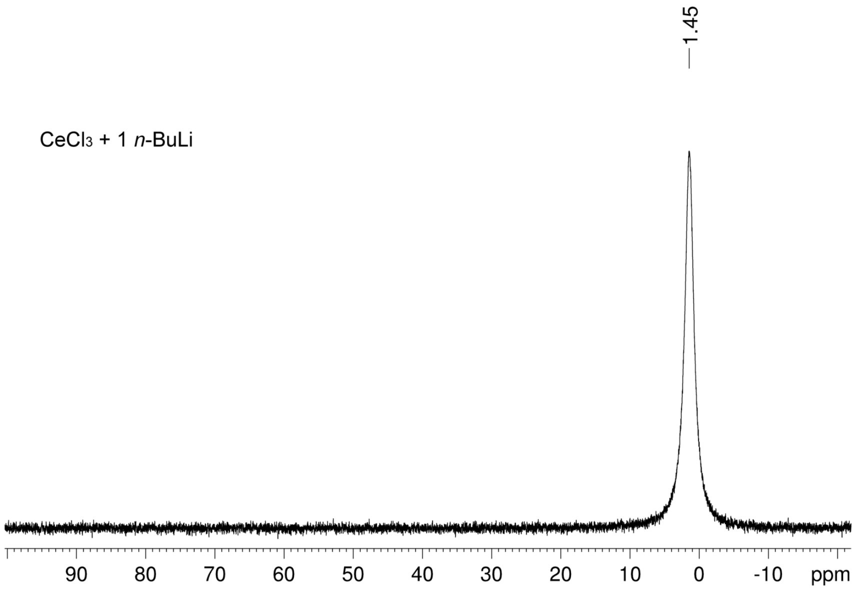

Figure S33. ^{7}Li NMR spectrum of $CeCl_3$(thf) + 1 equiv. of *n*-BuLi (194.37 MHz, THF-d_8, 233 K).

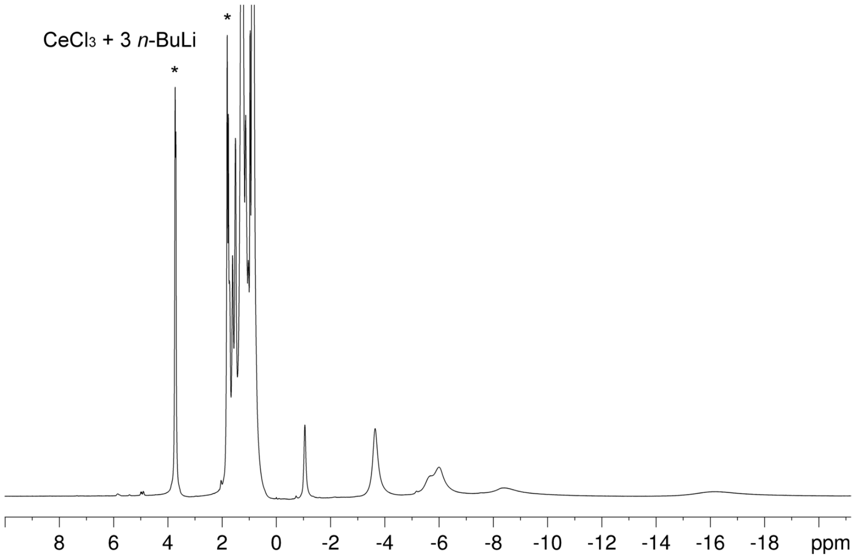

Figure S34. ^{1}H NMR spectrum of $CeCl_3$(thf) + 3 equivs. of *n*-BuLi (500.13 MHz, THF-d_8, 233 K). Solvent residual signals are marked with *.

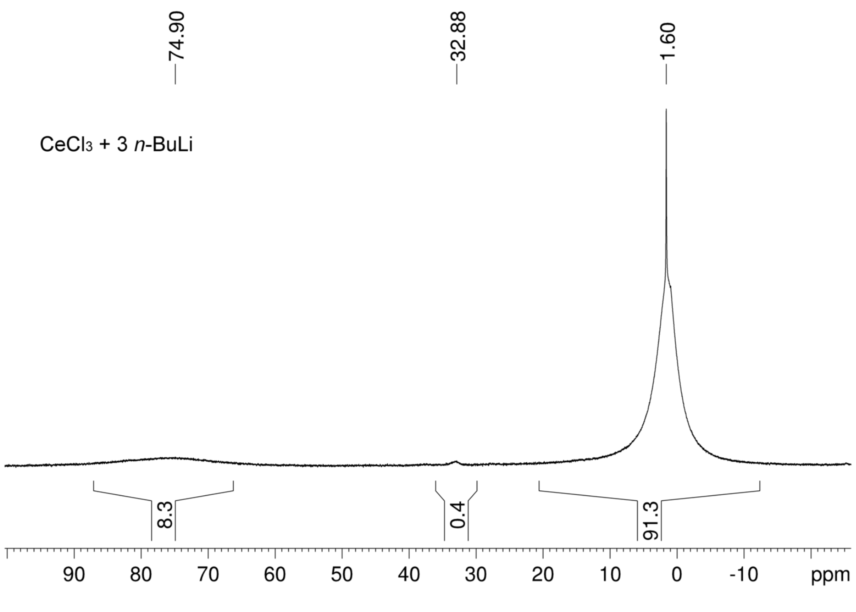

Figure S35. ^{7}Li NMR spectrum of $CeCl_3$(thf) + 3 equivs. of *n*-BuLi (194.37 MHz, THF-d_8, 233 K).

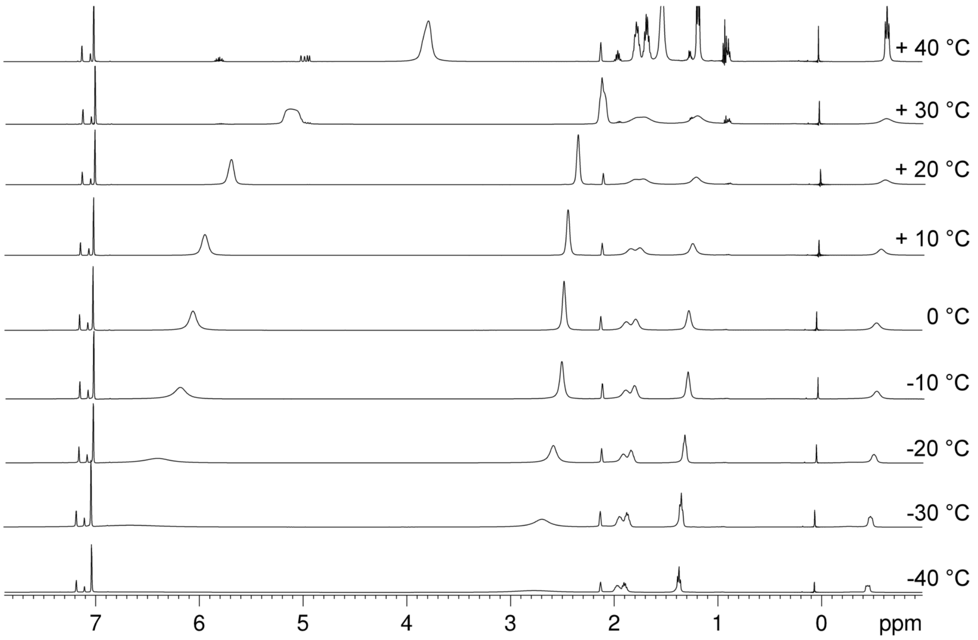

Figure S36. VT ^{1}H NMR spectra of $Li_3Ce(n\text{-}Bu)_6(thf)_4$ ($\mathbf{3^{Ce}}$) (500.13 MHz, toluene-d_8, 233 K-313 K), showing a slight amount of the decomposition product of 1-butene at 5.82 and 4.99 ppm at +30 °C and +40 °C.

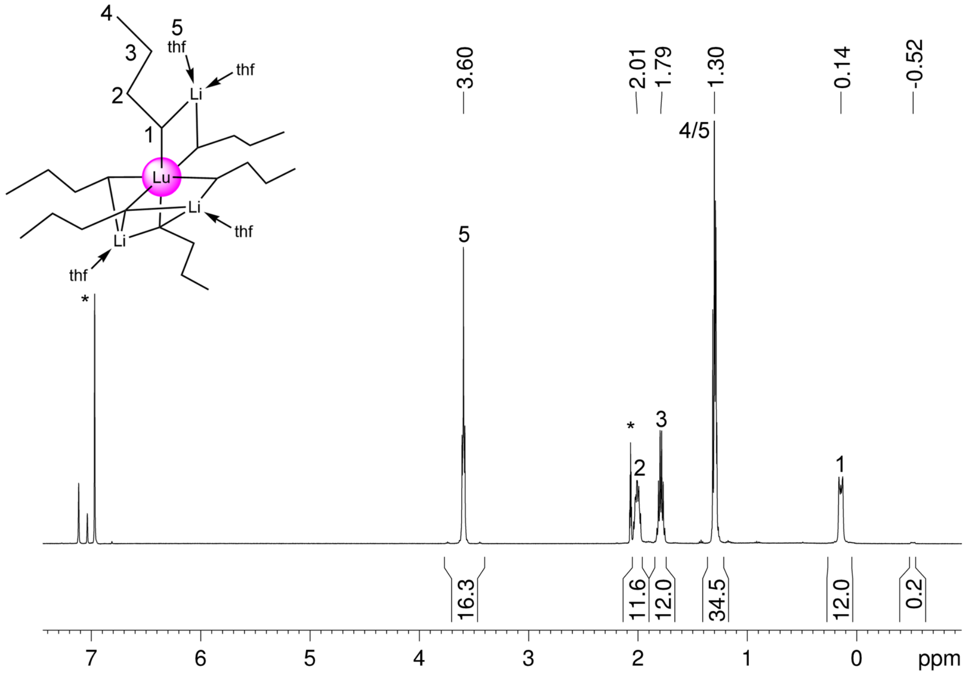

Figure S37. ^{1}H NMR spectrum of $Li_3Lu(n\text{-}Bu)_6(thf)_4$ ($\mathbf{3^{Lu}}$) (500.13 MHz, toluene-d_8, 233 K). Solvent residual signals are marked with *.

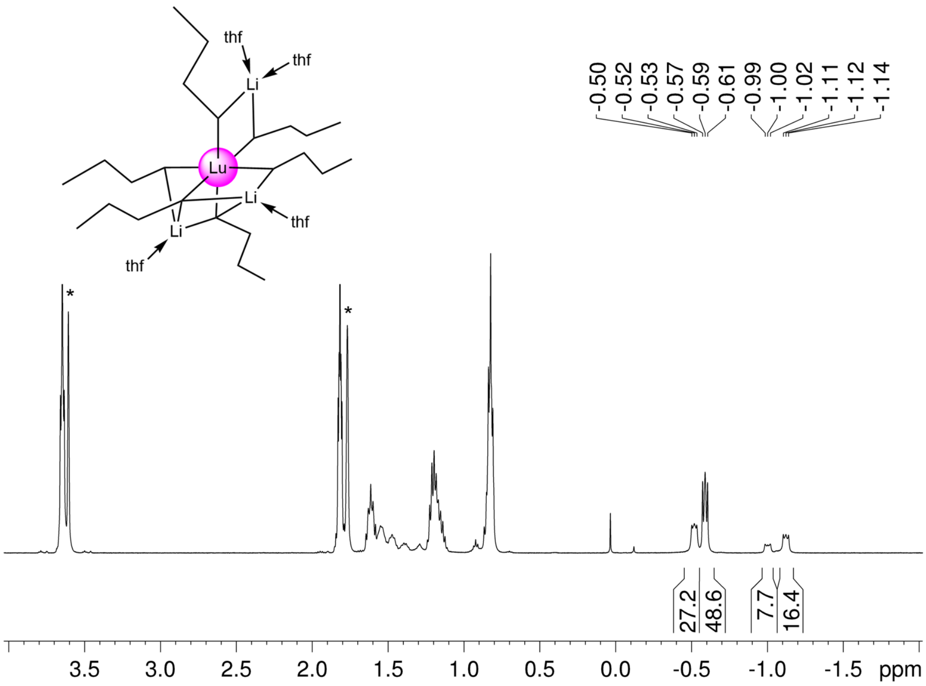

Figure S38. ^{1}H NMR spectrum of $Li_3Lu(n\text{-}Bu)_6(thf)_4$ ($\mathbf{3^{Lu}}$) (500.13 MHz, THF-d_8, 193 K). Solvent residual signals are marked with *. The signal set at –0.52 and –0.59 are from the CH_2 groups bonded to the lutetium and the signals at –1.00 and –1.12 are from dissociated *n*-BuLi(thf).

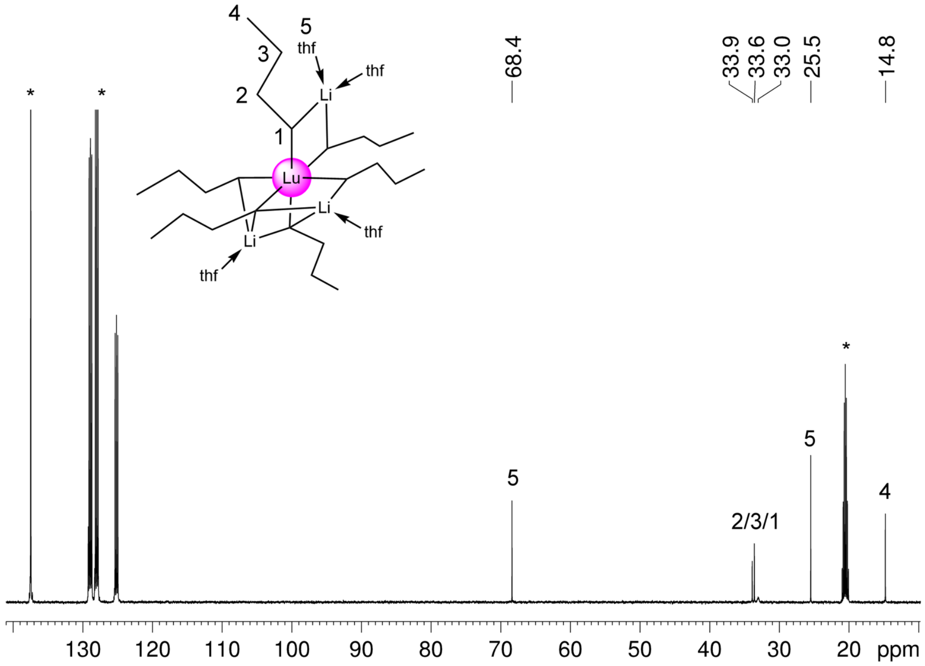

Figure S39. $^{13}C\{^{1}H\}$ NMR spectrum of $Li_3Lu(n\text{-}Bu)_6(thf)_4$ ($\mathbf{3^{Lu}}$) (125.76 MHz, toluene-d_8, 233 K). Solvent residual signals are marked with *.

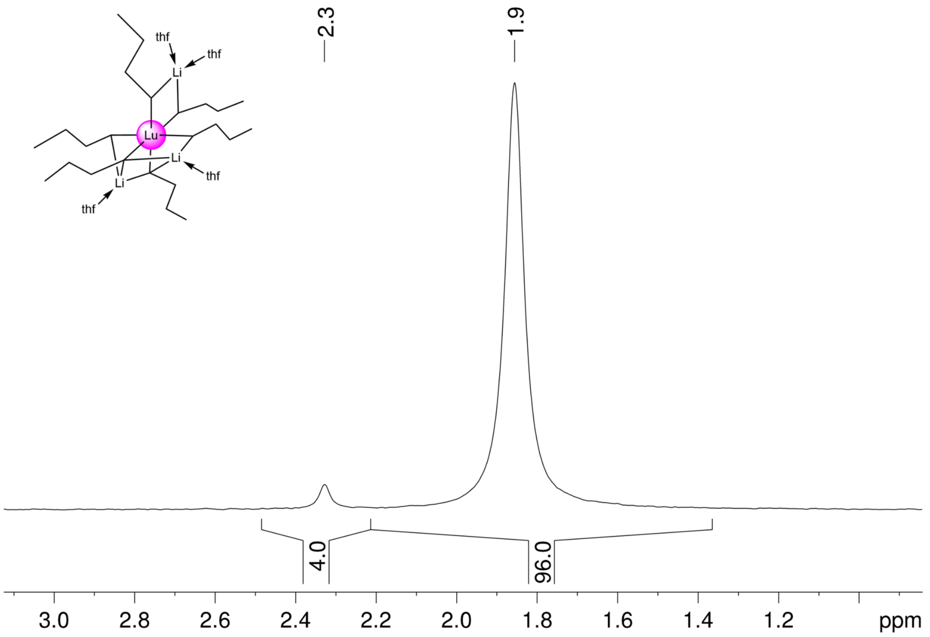

Figure S40. ^{7}Li NMR spectrum of $Li_3Lu(n\text{-}Bu)_6(thf)_4$ ($\mathbf{3^{Lu}}$) (194.37 MHz, toluene-d_8, 233 K), showing free *n*-BuLi(thf) at 2.3 ppm and the mixed lutetium–lithium complex at 1.9 ppm.

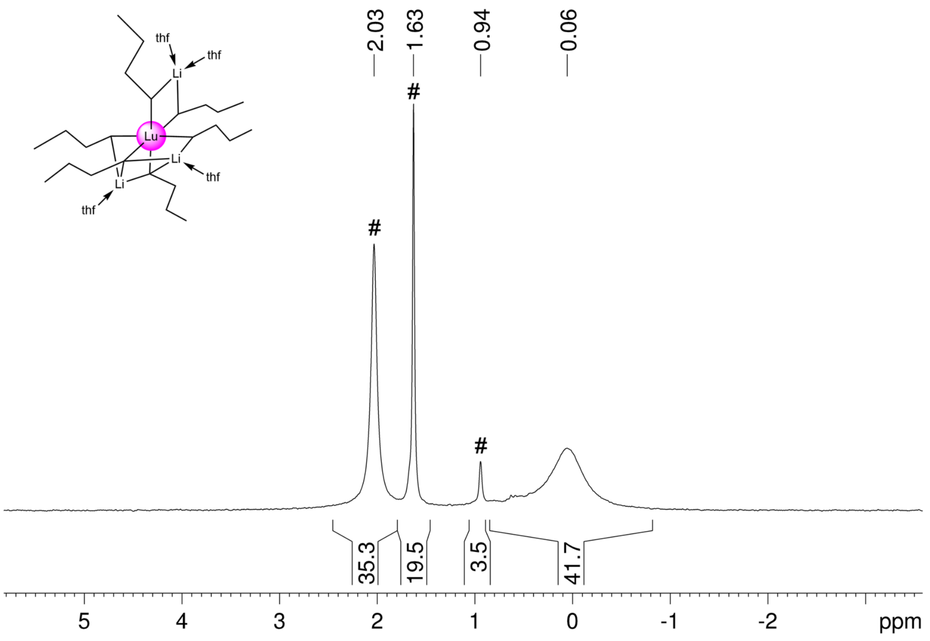

Figure S41. ^{7}Li NMR spectrum of $Li_3Lu(n\text{-}Bu)_6(thf)_4$ ($\mathbf{3^{Lu}}$) (194.37 MHz, THF-d_8, 193 K). Dissociated *n*-BuLi(thf) is marked with #.

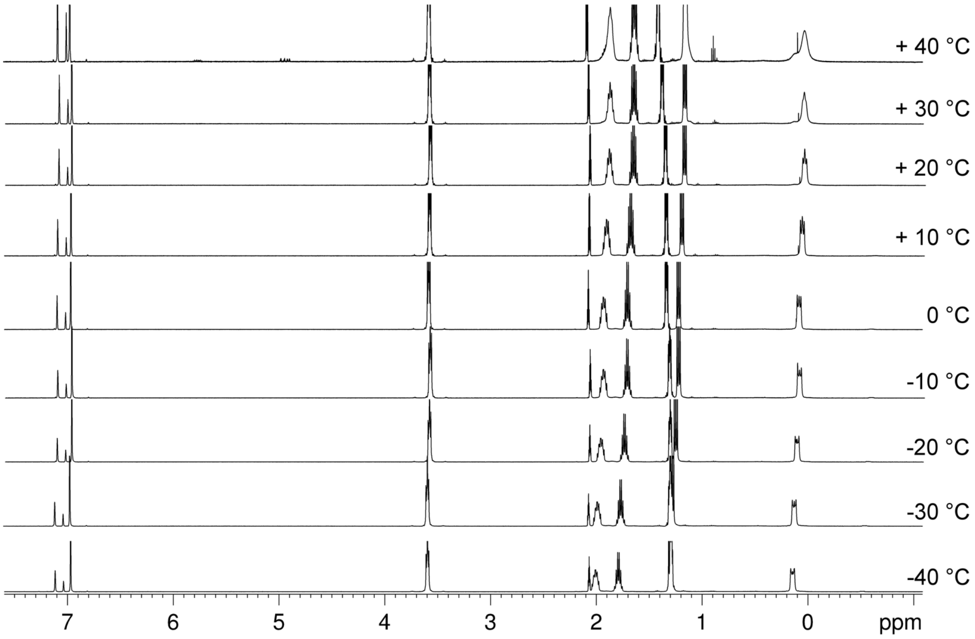

Figure S42. VT ^{1}H NMR spectra of $Li_3Lu(n\text{-}Bu)_6(thf)_4$ (**3^{Lu}**) (500.13 MHz, toluene-d_8, 233 K-313 K), showing a slight amount of the decomposition product 1-butene at 5.82 and 4.99 ppm at +30 °C and +40 °C.

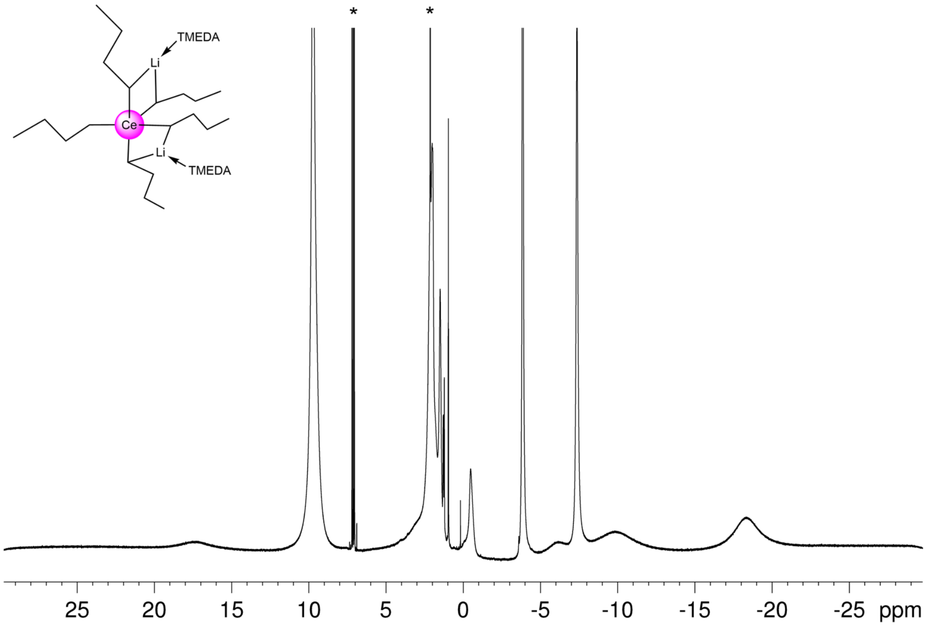

Figure S43. ^{1}H NMR spectrum of $Li_2Ce(n\text{-}Bu)_5(tmeda)_2$ (**4^{Ce}**) (500.13 MHz, toluene-d_8, 233 K). Solvent residual signals are marked with *. Except for the sharp *n*-BuLi(tmeda) signals, resonance assignment is not conclusive.

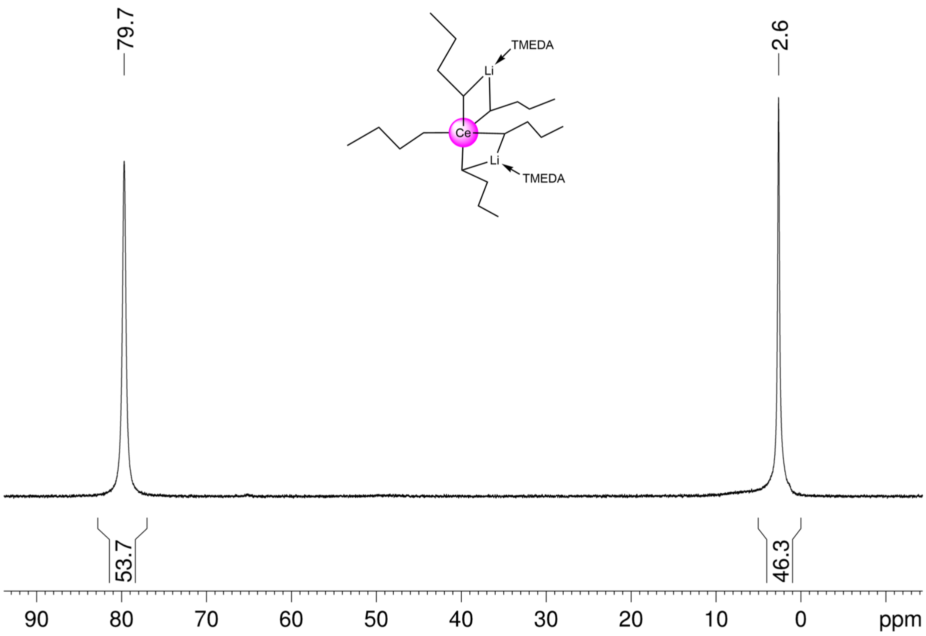

Figure S44. ^{7}Li NMR spectrum of $Li_2Ce(n\text{-}Bu)_5(tmeda)_2$ (**4^{Ce}**) (194.37 MHz, toluene-d_8, 233 K), showing the *n*-BuLi(tmeda) 2.6 ppm and a mixed cerium–lithium species at 79.7 ppm.

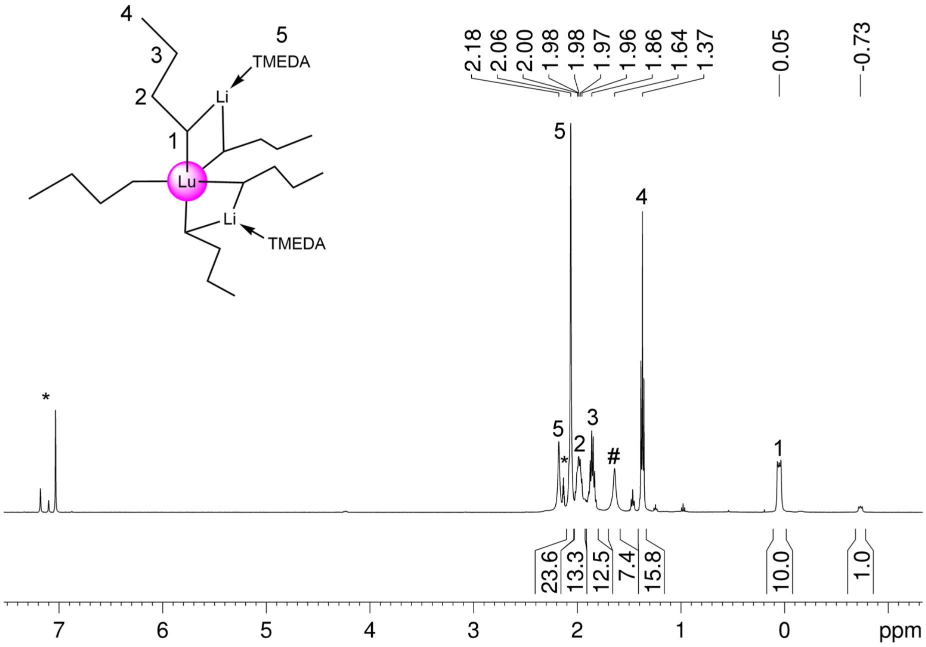

Figure S45. ^{1}H NMR spectrum of $Li_2Lu(n\text{-}Bu)_5(tmeda)_2$ (**4^{Lu}**) (500.13 MHz, toluene-d_8, 233 K). Solvent residual signals are marked with *. An unknown impurity is marked with #.

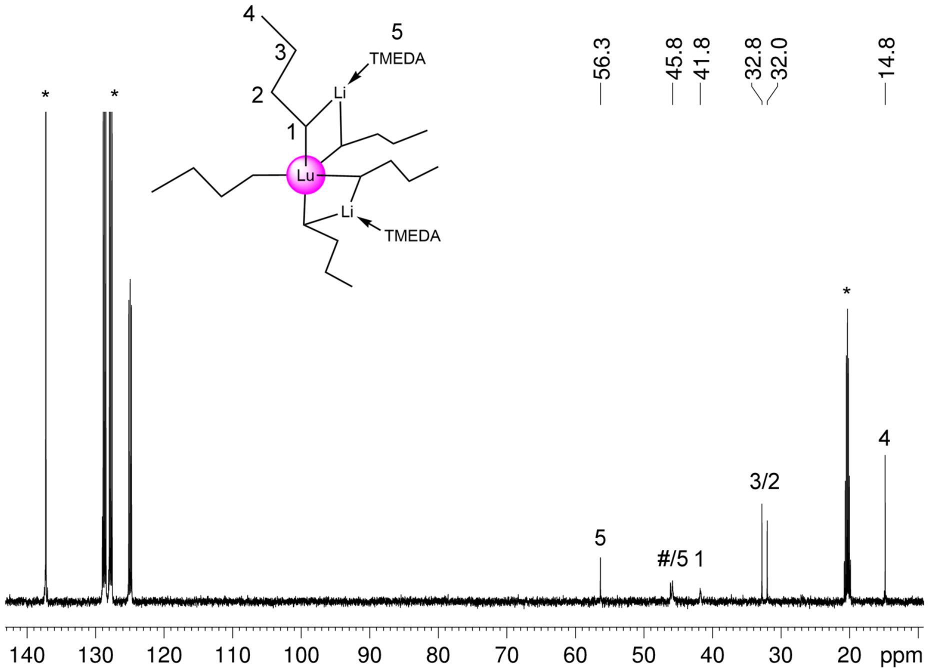

Figure S46. $^{13}C\{^1H\}$ NMR spectrum of $Li_2Lu(n\text{-}Bu)_5(tmeda)_2$ ($\mathbf{4^{Lu}}$) (125.76 MHz, toluene-d_8, 233 K). Solvent residual signals are marked with *. An unknown impurity is marked with #.

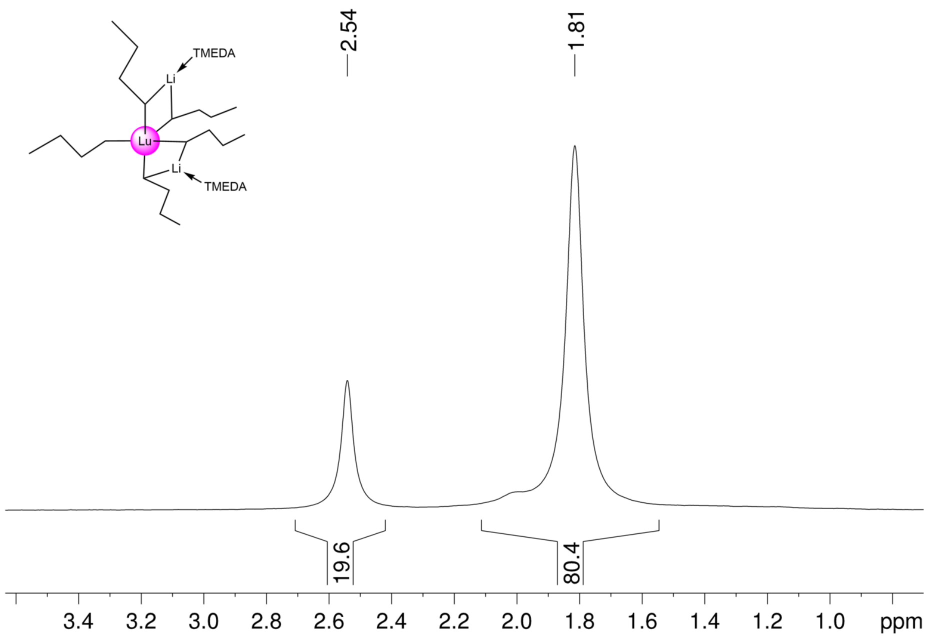

Figure S47. ^7Li NMR spectrum of $Li_2Lu(n\text{-}Bu)_5(tmeda)_2$ ($\mathbf{4^{Lu}}$) (194.37 MHz, toluene-d_8, 233 K), showing the n-BuLi(tmeda) at 2.5 ppm and a mixed lutetium–lithium species at 1.8 ppm.

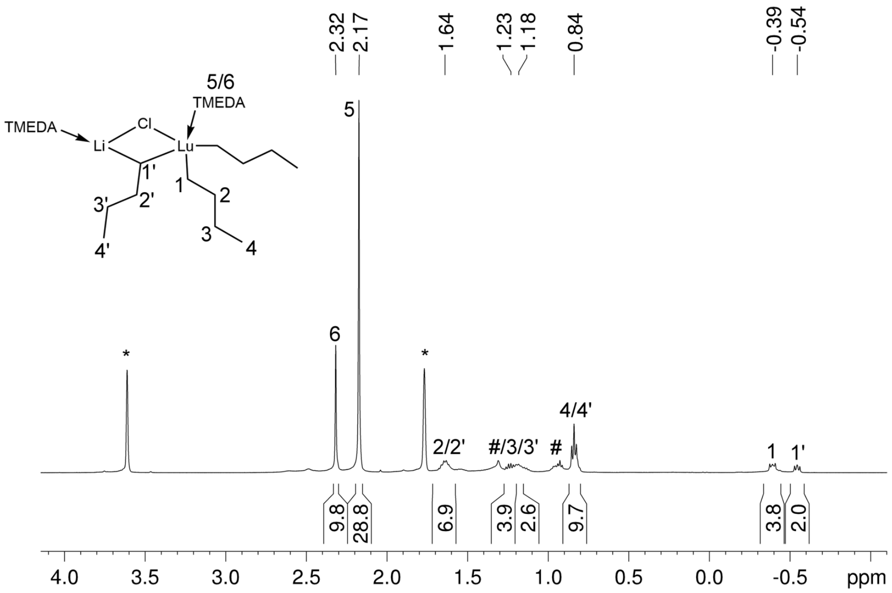

Figure S48. ^{1}H NMR spectrum of $LiLu(n\text{-}Bu)_3Cl(tmeda)_2$ (**5**) (500.13 MHz, THF-d_8, 233 K). Solvent residual signals are marked with *. Unknown impurities are marked with #.

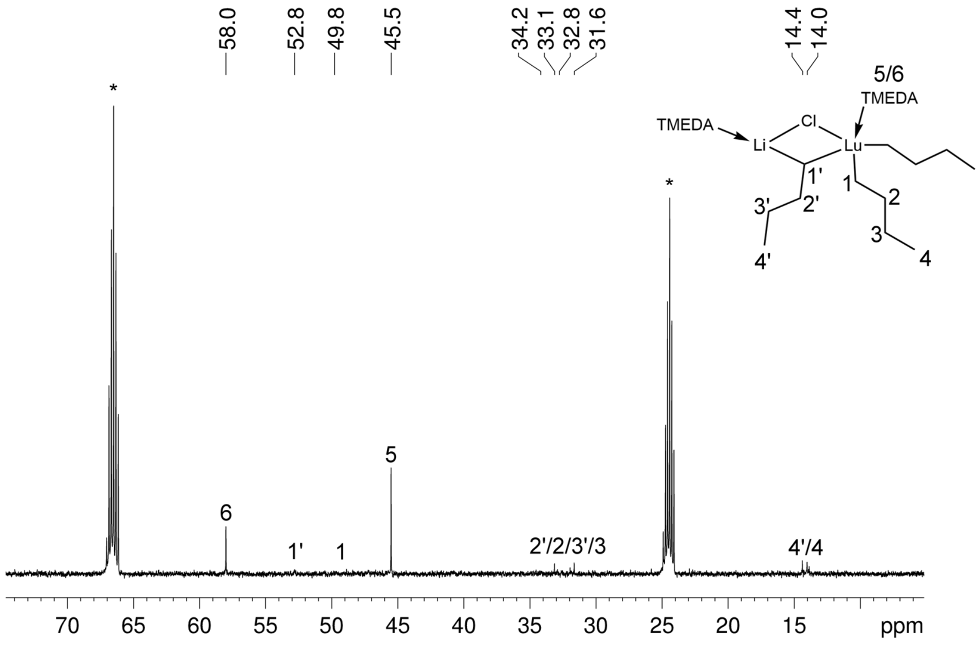

Figure S49. $^{13}C\{^{1}H\}$ NMR spectrum of $LiLu(n\text{-}Bu)_3Cl(tmeda)_2$ (**5**) (125.76 MHz, THF-d_8, 233 K). Solvent residual signals are marked with *.

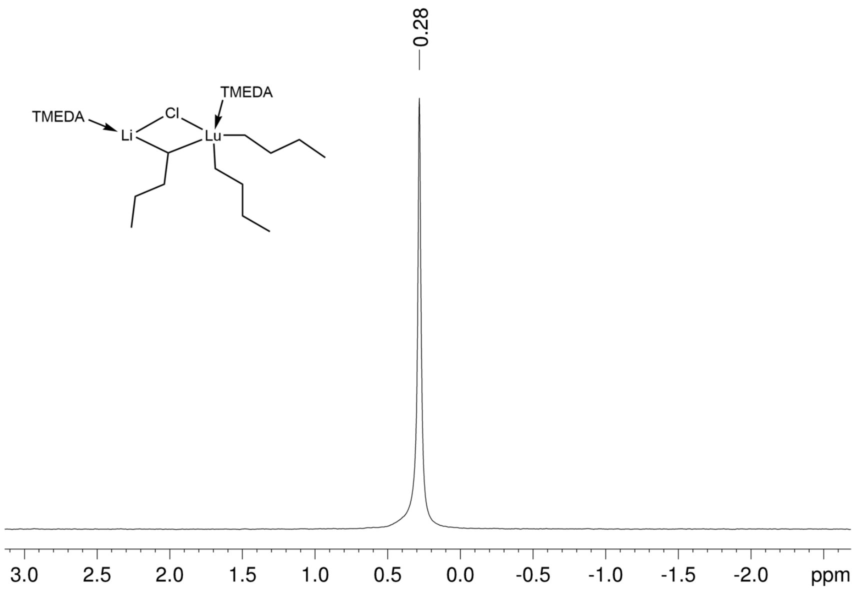

Figure S50. ^{7}Li NMR spectrum of LiLu(n-Bu)$_3$Cl(tmeda)$_2$ **(5)** (194.37 MHz, THF-d_8, 233 K).

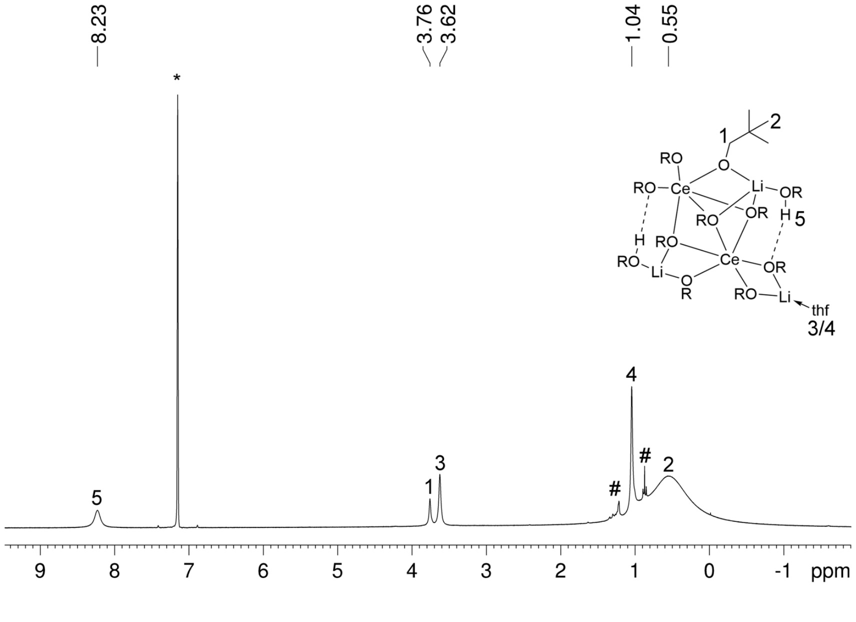

Figure S51. ^{1}H NMR spectrum of $Li_3Ce_2(ONep)_9(HONep)_2$(thf) (**6**) (300.13 MHz, benzene-d_6, 299 K). The solvent residual signal is marked with *. Residual n-hexane is marked with #.

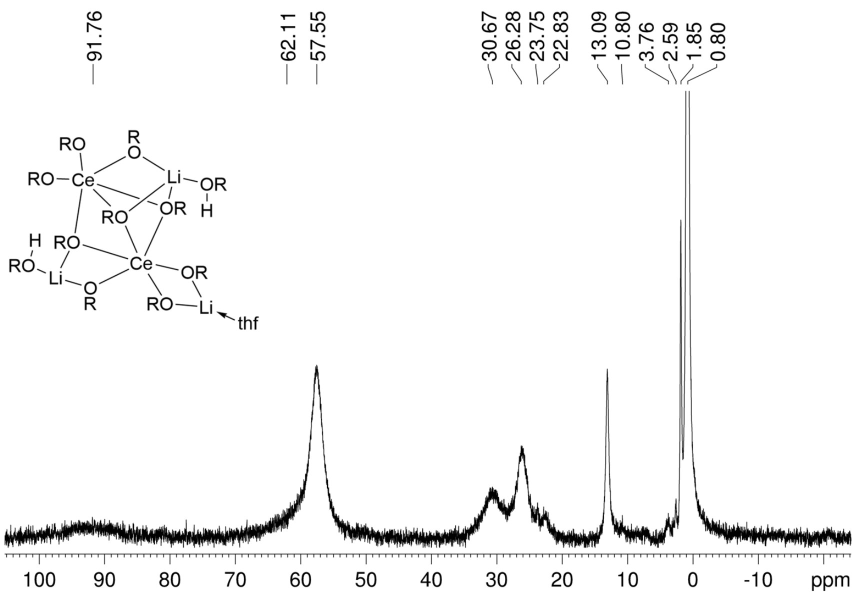

Figure S52. ^{7}Li NMR spectrum of $Li_3Ce_2(ONep)_9(HONep)_2(thf)$ (**6**) (116.64 MHz, benzene-d_6, 299 K).

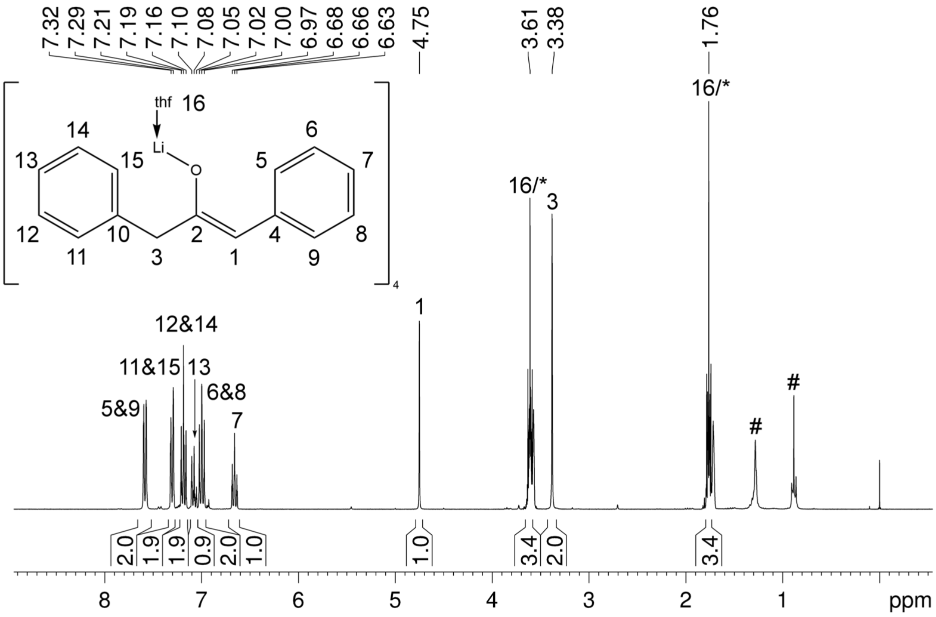

Figure S53. ^{1}H NMR spectrum of $Li_4[OC(=CHPh)(CH_2Ph)]_4(thf)_4$ (**7**) (300.13 MHz, THF-d_8, 299 K). Solvent residual signals are marked with *. Residual *n*-hexane is marked with #.

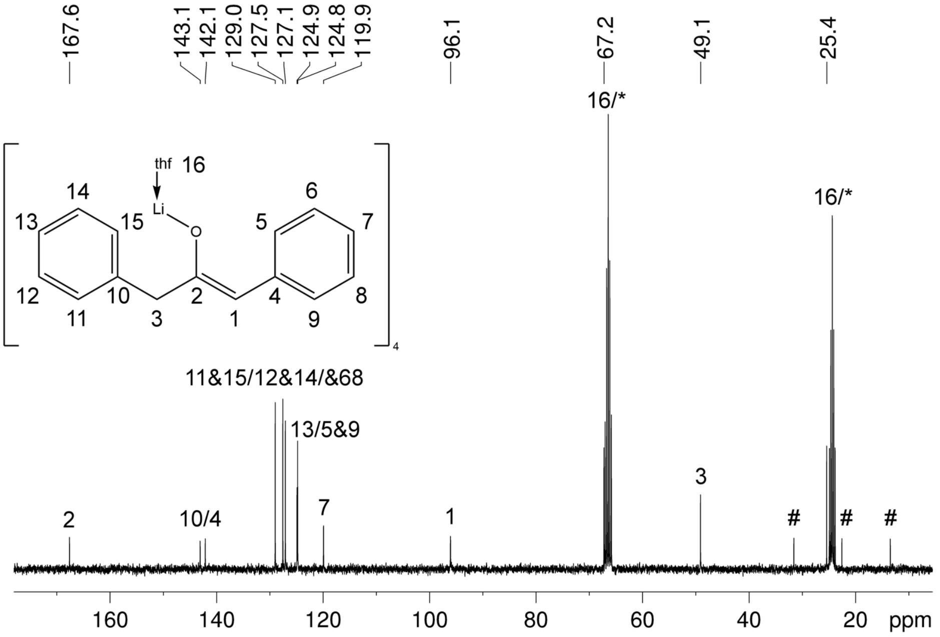

Figure S54. $^{13}C\{^1H\}$ NMR spectrum of $Li_4[OC(=CHPh)(CH_2Ph)]_4(thf)_4$ (**7**) (75.47 MHz, THF-d_8, 299 K). Solvent residual signals are marked with *. Residual *n*-hexane is marked with #.

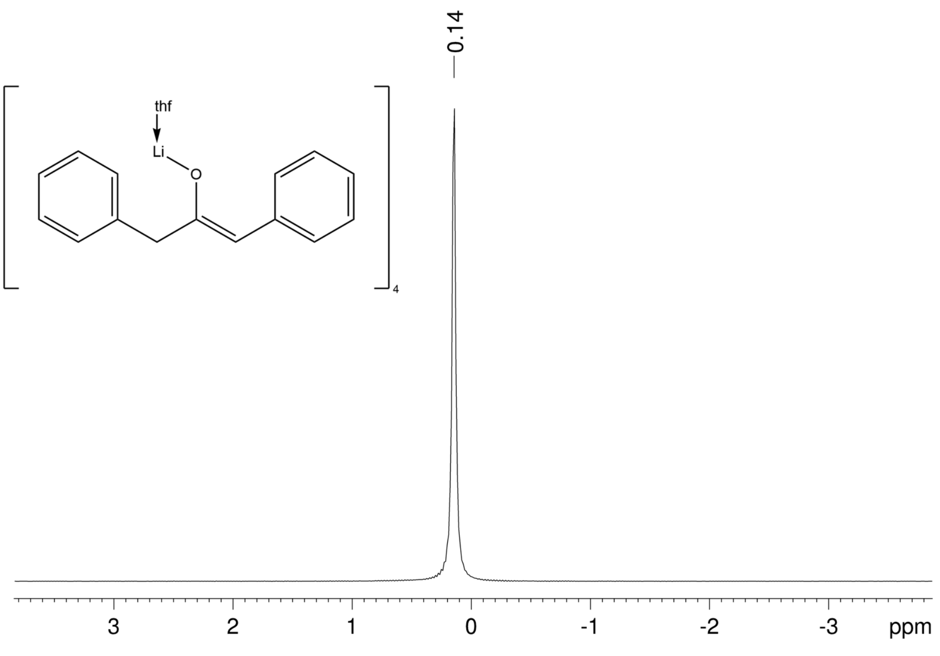

Figure S55. ^7Li NMR spectrum of $Li_4[OC(=CHPh)(CH_2Ph)]_4(thf)_4$ (**7**) (116.64 MHz, THF-d_8, 299 K).

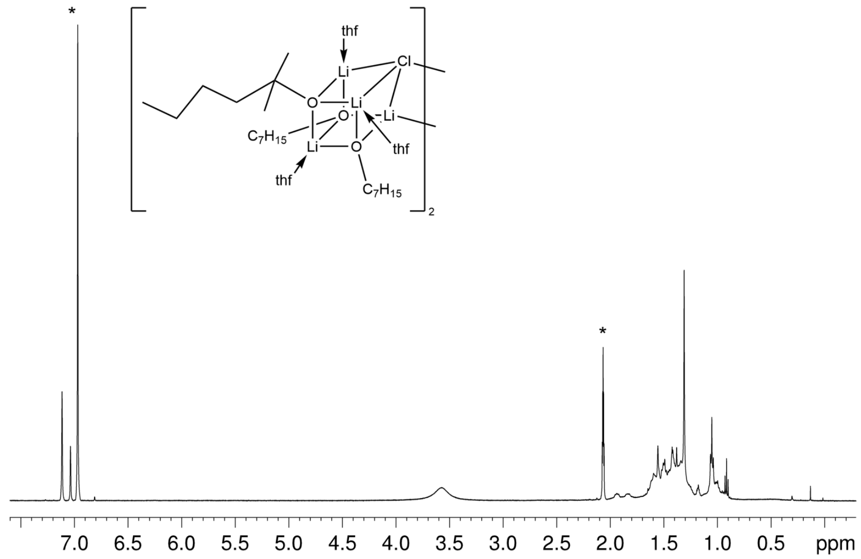

Figure S56. ^{1}H NMR spectrum of $Li_8[OCMe_2(n\text{-Bu})]_6Cl_2(thf)_6$ (**8**) (500.13 MHz, toluene-d_8, 233 K). Solvent residual signals are marked with *.

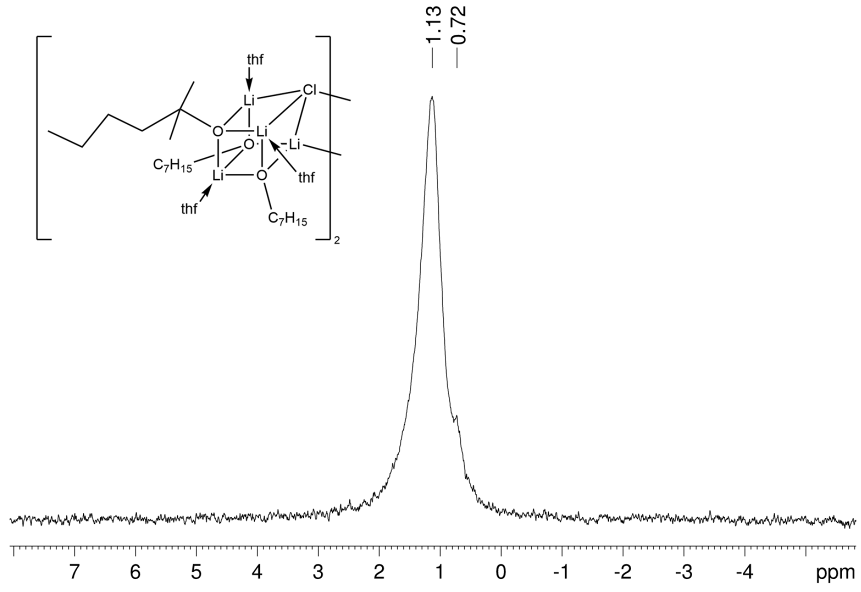

Figure S57. ^{7}Li NMR spectrum of $Li_8[OCMe_2(n\text{-Bu})]_6Cl_2(thf)_6$ (**8**) (194.37 MHz, toluene-d_8, 233 K).

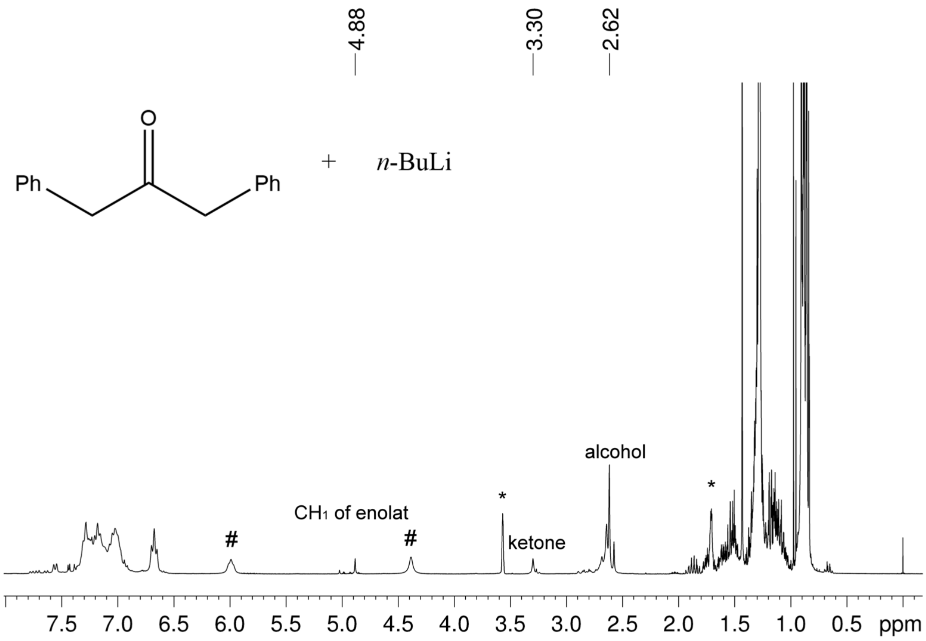

Figure S58. ^{1}H NMR spectrum of the reaction of 1,3-diphenylpropan-2-one and *n*-BuLi (300.13 MHz, THF-d_8, 299 K). Solvent residual signals are marked with *. An unknown impurity is marked with #.

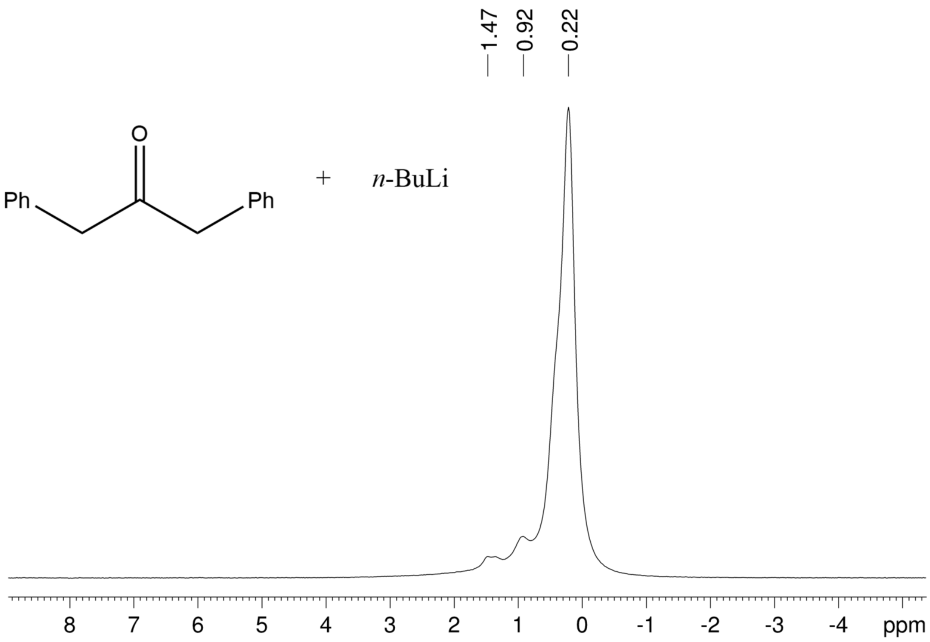

Figure S59. ^{7}Li NMR spectrum of the reaction of 1,3-diphenylpropan-2-one with *n*-BuLi (116.64 MHz, THF-d_8, 299 K).

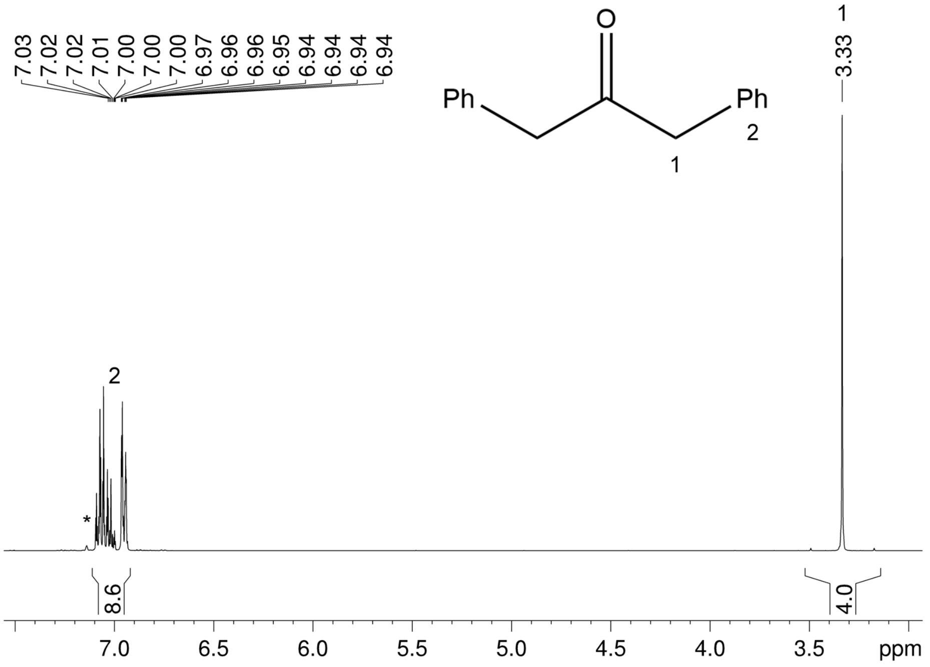

Figure S60. ^{1}H NMR spectrum of 1,3-diphenylpropan-2-one (400.11 MHz, benzene-d_6, 299 K). The solvent residual signal is marked with *.

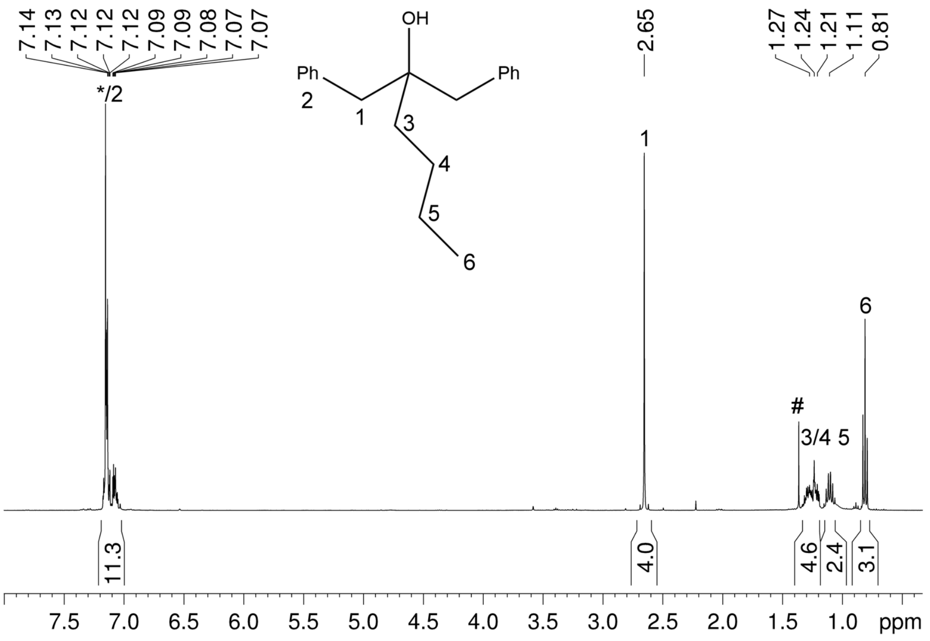

Figure S61. ^{1}H NMR spectrum of 1,3-diphenyl-2-butylpropan-2-ol (400.11 MHz, benzene-d_6, 299 K). The solvent residual signal is marked with *. Residual acetone impurity is marked with #.

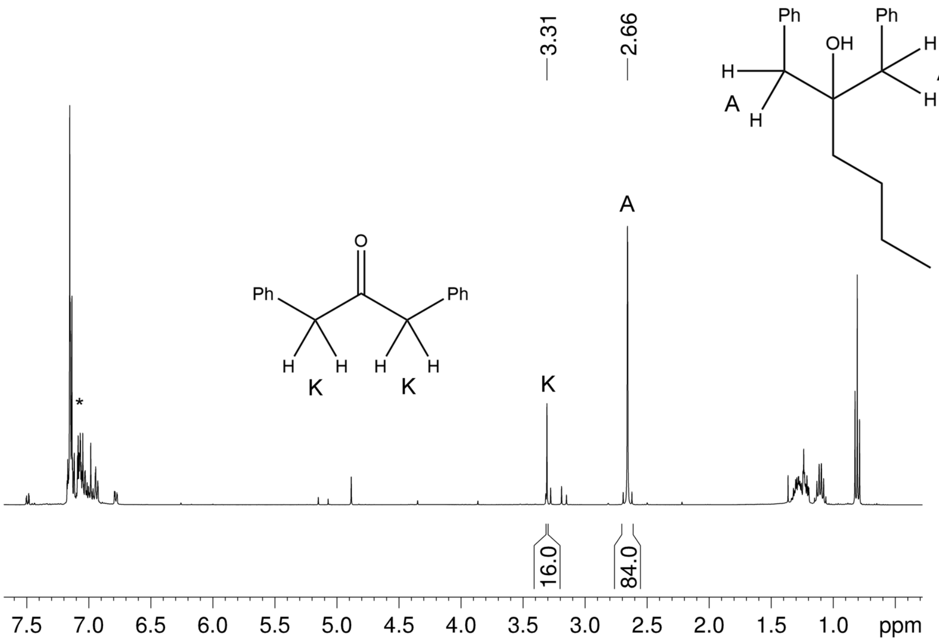

Figure S62. ^{1}H NMR spectrum of a mixture of 1,3-diphenylpropan-2-one and 1,3-diphenyl-2-butylpropan-2-ol from the reaction of $Li_3Ce(n\text{-}Bu)_6(thf)_4$ (**3**^{Ce}) with six equiv. of 1,3-diphenylpropan-2-one (400.11 MHz, benzene-d_6, 299 K). The solvent residual signal is marked with *.

References

1. A. Krasovskiy, F. Kopp, P. Knochel , *Angew. Chem. Int. Ed.* **2006**, *45*, 497-500; *Angew. Chem.* **2006**, *118*, 511-515.
2. G. R. Fulmer, A. J. M. Miller, N. H. Sherden, H. E. Gottlieb, A. Nudelman, B. M. Stoltz, J. E. Bercaw, K. I. Goldberg, *Organometallics* **2010**, *29*, 2176-2179.
3. TopSpin v. 3.6.1, Bruker AXS Inc., Madison, WI. 2018.
4. COSMO v. 1.61, Bruker AXS Inc., Madison, WI,. 2012.
5. APEX 3 v. 2017.3-0, Bruker AXS Inc., Madison, WI. 2017.
6. SAINT v. 8.38A, Bruker AXS Inc., Madison, WI. 2017.
7. L. Krause, R. Herbst-Irmer, G. M. Sheldrick, D. Stalke, *J. Appl. Crystallogr.* **2015**, *48*, 3-10.
8. G. Sheldrick, *Acta Crystallogr. Sect. C.* **2015**, *71*, 3-8.
9. C. B. Hubschle, G. M. Sheldrick, B. Dittrich, *J. Appl. Crystallogr.* **2011**, *44*, 1281-1284.
10. D. Kratzert, J. J. Holstein, I. Krossing, *J. Appl. Crystallogr.* **2015**, *48*, 933-938.
11. C. F. Macrae, P. R. Edgington, P. McCabe, E. Pidcock, G. P. Shields, R. Taylor, M. Towler, J. van de Streek, *J. Appl. Crystallogr.* **2006**, *39*, 453-457.
12. POV-Ray v.3.6, Persistence of Vision Pty. Ltd., POV-Ray Williamstown, Victoria, Australia. http://www.povray.org, 2004.
13. H. Schumann, J. Mueller, N. Bruncks, H. Lauke, J. Pickardt, H. Schwarz, K. Eckart, *Organometallics* **1984**, *3*, 69-74.
